# Quantitative analysis of tRNA abundance and modifications by nanopore RNA sequencing

**DOI:** 10.1038/s41587-023-01743-6

**Published:** 2023-04-06

**Authors:** Morghan C. Lucas, Leszek P. Pryszcz, Rebeca Medina, Ivan Milenkovic, Noelia Camacho, Virginie Marchand, Yuri Motorin, Lluís Ribas de Pouplana, Eva Maria Novoa

**Affiliations:** 1 Centre for Genomic Regulation (CRG), Barcelona Institute of Science and Technology, Barcelona, Spain; 2 Universitat Pompeu Fabra (UPF), Barcelona, Spain; 3 Institute for Research in Biomedicine (IRB), Barcelona, Spain; 4 CNRS-Université de Lorraine, UAR2008 IBSLor/UMR7365 IMoPA, Nancy, France; 5 Catalan Institution for Research and Advanced Studies (ICREA), Barcelona, Spain

**Keywords:** RNA sequencing, Small RNAs

## Abstract

Transfer RNAs (tRNAs) play a central role in protein translation. Studying them has been difficult in part because a simple method to simultaneously quantify their abundance and chemical modifications is lacking. Here we introduce Nano-tRNAseq, a nanopore-based approach to sequence native tRNA populations that provides quantitative estimates of both tRNA abundances and modification dynamics in a single experiment. We show that default nanopore sequencing settings discard the vast majority of tRNA reads, leading to poor sequencing yields and biased representations of tRNA abundances based on their transcript length. Re-processing of raw nanopore current intensity signals leads to a 12-fold increase in the number of recovered tRNA reads and enables recapitulation of accurate tRNA abundances. We then apply Nano-tRNAseq to *Saccharomyces cerevisiae* tRNA populations, revealing crosstalks and interdependencies between different tRNA modification types within the same molecule and changes in tRNA populations in response to oxidative stress.

## Abstract (web summary)

tRNA abundance and chemical modifications are measured simultaneously with nanopores.

## Main

Transfer RNAs (tRNAs) are abundant small non-coding RNAs that play a pivotal role in decoding genetic information^1–3^. Based on their aminoacylation identity, tRNAs are subdivided into 20 accepting groups (alloacceptors), each comprising several tRNAs that translate synonymous codons with the same amino acid (isoacceptors). To fulfill their function as adapter molecules between the RNA and protein codes, tRNAs are extensively modified, containing on average 13 modifications per tRNA molecule^4^. Although some tRNA modifications are thought to be structural and static, others are dynamic and even reversible^5–8^, affecting the fate and function of individual tRNA molecules^2,9–13^. Notably, mutations in multiple tRNA modification enzymes have been associated with a wide variety of human diseases^14–17^, highlighting their importance in proper cellular functioning.

tRNA modifications are present in all domains of life^18,19^ and are synthesized by dedicated tRNA-modifying enzymes that alter specific tRNAs in a site-specific fashion^20^. The chemical nature of these modifications is highly diverse and includes methylations, acetylations, isomerizations, deaminations and conjugation to amino acids, among others^21,22^. Certain tRNA modifications are found only in a single tRNA species, whereas others are found in multiple tRNA species^20,23^. For example, 2-lysidine (k^2^C) tRNA modifications are placed at position 34 of the anticodon of tRNA^Ile^(AUA)^24^, whereas pseudouridine (Ψ) can be placed at diverse positions of the tRNA molecule and in multiple tRNA isoacceptors^25–27^. Regarding their function, tRNA modifications can sometimes act as identity elements recognized by aminoacyl-tRNA synthetases^28–30^, and, without modifications, many tRNAs have poor aminoacylation capability^31^. On the other hand, tRNA modifications can affect the decoding preferences of tRNA molecules, especially those found at position 34 of the anticodon^16,32–34^, restricting or increasing the wobbling capacity of the tRNAs and, consequently, changing the set of ‘preferred’ or ‘optimal’ codons that will be translated^35–44^.

In the last few years, it has been shown that some tRNA modifications are reversible^8,45–47^ and can be dynamically regulated upon environmental exposures^48–50^, across cell cycle stages^51^ and upon tumorigenesis^52–56^. Similarly, tRNA abundances are also dysregulated upon environmental exposures, such as oxidative stress^48,57,58^, as well as in diverse types of cancer^52,59–61^. Modulation of tRNA abundances and/or tRNA modifications is generally regarded as a molecular strategy that allows cells to adapt to distinct physiological states or conditions, leading to increased expression of subsets of proteins that otherwise would remain poorly translated under ‘normal’ tRNA abundances^60,62,63^.

**Fig. 1:**
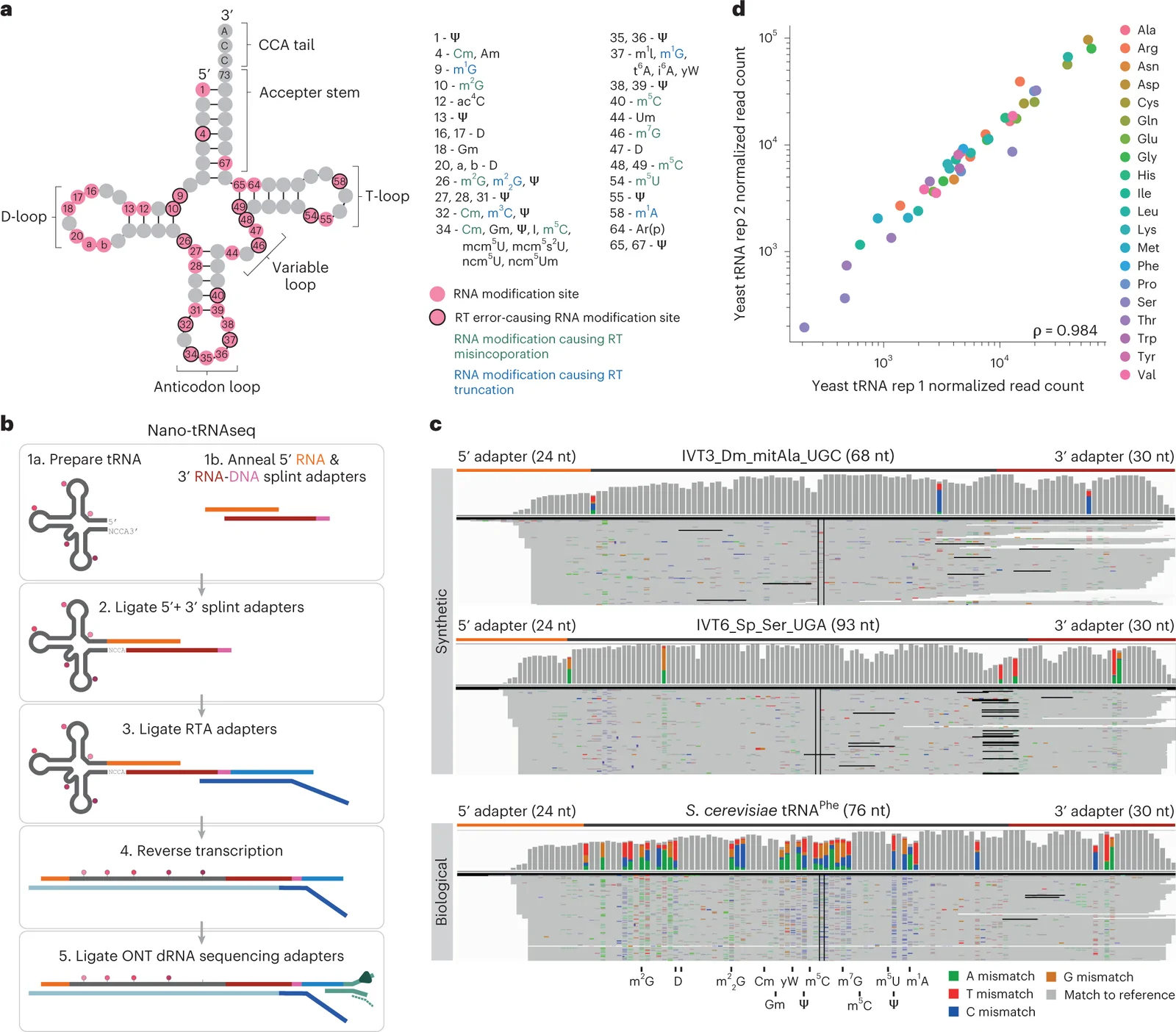
Nano-tRNAseq can efficiently sequence both IVT and native tRNA populations. **a**, Schematic of the modifications found in *S. cerevisia*e cytoplasmic tRNA, shown in its usual secondary structure form with circles representing nucleotides and lines representing base pairs. Gray circles represent unmodified nucleotides; pink circles represent possible modification sites; and those with a black outline indicate modifications that cause errors during reverse transcription. Possible RNA modifications occurring at each position are listed in the surrounding boxes; modifications that cause misincorporation during reverse transcription are in green; and those that cause reverse transcription truncation are in blue. **b**, Schematic overview illustrating the steps required for tRNA library preparation using Nano-tRNAseq (see Extended Data Fig. 1 for more details). **c**, IGV snapshots of Nano-tRNAseq mapped reads from synthetic IVT tRNAs (upper panels) or biological tRNAs (lower panel). Positions with a mismatch frequency greater than 0.2 are colored, whereas those showing mismatch frequencies lower than 0.2 are shown in gray. **d**, Scatter plot of tRNA abundances showing the replicability of Nano-tRNAseq when WT *S. cerevisia*e tRNA biological replicates are sequenced. The correlation strength is indicated by Spearman’s correlation coefficient (ρ). RT, reverse transcription.

Despite the pivotal function that tRNAs play in cellular processes and their involvement in numerous human diseases, we currently lack a simple and cost-effective method to accurately quantify both tRNA abundances and their modifications systematically. On the one hand, tRNA modifications are typically identified and quantified with high accuracy using liquid chromatography coupled to mass spectrometry (LC–MS) methodologies^48,64–69^. In these methods, RNA molecules are fragmented into oligomers or monomers, and their abundance is measured via UV absorption or MS/MS. LC–MS/MS techniques using triple quadrupole-based detection are among the most sensitive, allowing limits of quantification in the low femtomole range^48,64,70–72^, but they typically cannot identify the tRNA isoacceptor that contained each detected modification. On the other hand, tRNA abundances can be determined using tRNA microarrays^36,59,73^ and, more recently, by employing next-generation sequencing (NGS) technologies^74–82^, which require an initial conversion of the tRNA molecules into cDNA. Consequently, NGS-based methods are blind to most tRNA modifications, as these are typically erased during the reverse transcription step. Moreover, tRNA modifications that disrupt the Watson–Crick base pairing, which are abundant in tRNAs, will interfere with the reverse transcriptase enzyme, causing it to drop off, producing truncated reads, in addition to misincorporations^72,83–85^ (Fig. 1a). To overcome these limitations, a wide variety of improved tRNA sequencing protocols have been developed in recent years, which often include the use of highly processive reverse transcriptase enzymes^75,77^ and/or cocktails of demethylases^74,75,79^. However, despite these improvements, NGS-based methods still suffer from the following caveats: (1) they introduce significant biases during the library preparation, caused by incomplete reverse transcriptions^84,86^, incomplete demethylations^87^ and polymerase chain reaction (PCR) amplification^88^, resulting in skewed representations of existing tRNA populations; and (2) they cannot detect most tRNA modifications, as these are typically ‘erased’ during the conversion of RNA to cDNA. Therefore, a simple, robust and efficient tRNA sequencing method is still needed.

A promising alternative to the use of NGS-based technologies to characterize the tRNAome is the direct RNA sequencing (DRS) platform developed by Oxford Nanopore Technologies (ONT). This technology allows direct sequencing of native RNA molecules and, as such, can, in principle, detect and measure both tRNA modifications and tRNA abundances without the need for reverse transcription or PCR. Previous works have demonstrated that nanopores can capture tRNAs using solid-state or biological (ONT) nanopores^89–92^. For example, sequencing of five distinct tRNAs was achieved using solid-state nanopores^89^, and tRNAs were shown to be distinguishable from other short RNAs using the MspA pore^90^. Later studies showed that, by lengthening the tRNA molecules with ligated adapter extensions, tRNAs could be sequenced, basecalled and mapped using biological nanopores^92^. However, in these studies, the proposed approach led to low sequencing yields of tRNA molecules (~20–40× lower than expected for a DRS run) and did not report whether extant in vivo tRNA abundances and/or tRNA modifications were recapitulated using this method.

Here we present Nano-tRNAseq, a nanopore-based approach that allows accurate and direct measurement of native tRNA molecules. The library preparation benefits from the 3′ CCA overhang typically present in the mature tRNAs to incorporate a double ligation of RNA adapters at both the 5′ and 3′ ends of the tRNA molecules, which leads to an improved proportion of basecalled and mapped tRNA molecules. Moreover, we show that MinKNOW, the ONT proprietary software required to run nanopore sequencing experiments, erroneously discards the majority of tRNA reads, misinterpreting them as ‘adapters’, and also causes biases in the estimated tRNA abundances due to preferential capture of longer tRNAs (for example, tRNA^Leu^, tRNA^Arg^ and tRNA^Ser^). To overcome these limitations, here we provide a computational framework that allows us to capture ~10× more tRNA reads and accurately recapitulates tRNA abundances.

Altogether, our work provides a simple, cost-effective, high-throughput and reproducible method to accurately quantify tRNA abundances and capture tRNA modification changes simultaneously using native tRNA nanopore sequencing, providing a framework to study the tRNAome at single-molecule resolution. We envision that Nano-tRNAseq will contribute to the study of the biological function of tRNA modifications in a wide variety of contexts, such as cancer, stress exposures or viral infection, and opens the possibility of exploiting these molecules as biomarkers of human health and disease.

## Results

### Standard nanopore DRS results in low tRNA sequencing yields

Nanopore DRS is a well-established long-read sequencing technology to study RNA molecules, typically polyadenylated mRNAs^93–97^. Although several works have shown that this technology can also be used to study short RNA molecules, such as snoRNAs and snRNAs^96,98^, DRS is inefficient at capturing RNA molecules shorter than 200 nucleotides (nt) and is generally considered unable to capture sequences shorter than ~100 nt^96,97^, limiting its applicability to study short RNA populations, such as tRNAs. In addition, the first ~15 nt at the 5′ end of RNA molecules are typically lost in DRS runs^93,99^, as this portion cannot be adequately basecalled due to the increase in the RNA translocation speed when the 5′ end of the molecule exits the helicase^99^. To overcome these limitations, we reasoned that extension of the 5′ and 3′ ends of the tRNA would lead to improved sequencing of tRNA molecules, as these would now be beyond the ~100-nt threshold, in addition to capturing the sequence and modification information of 5′ tRNA ends.

**Extended Data Fig. 1:**
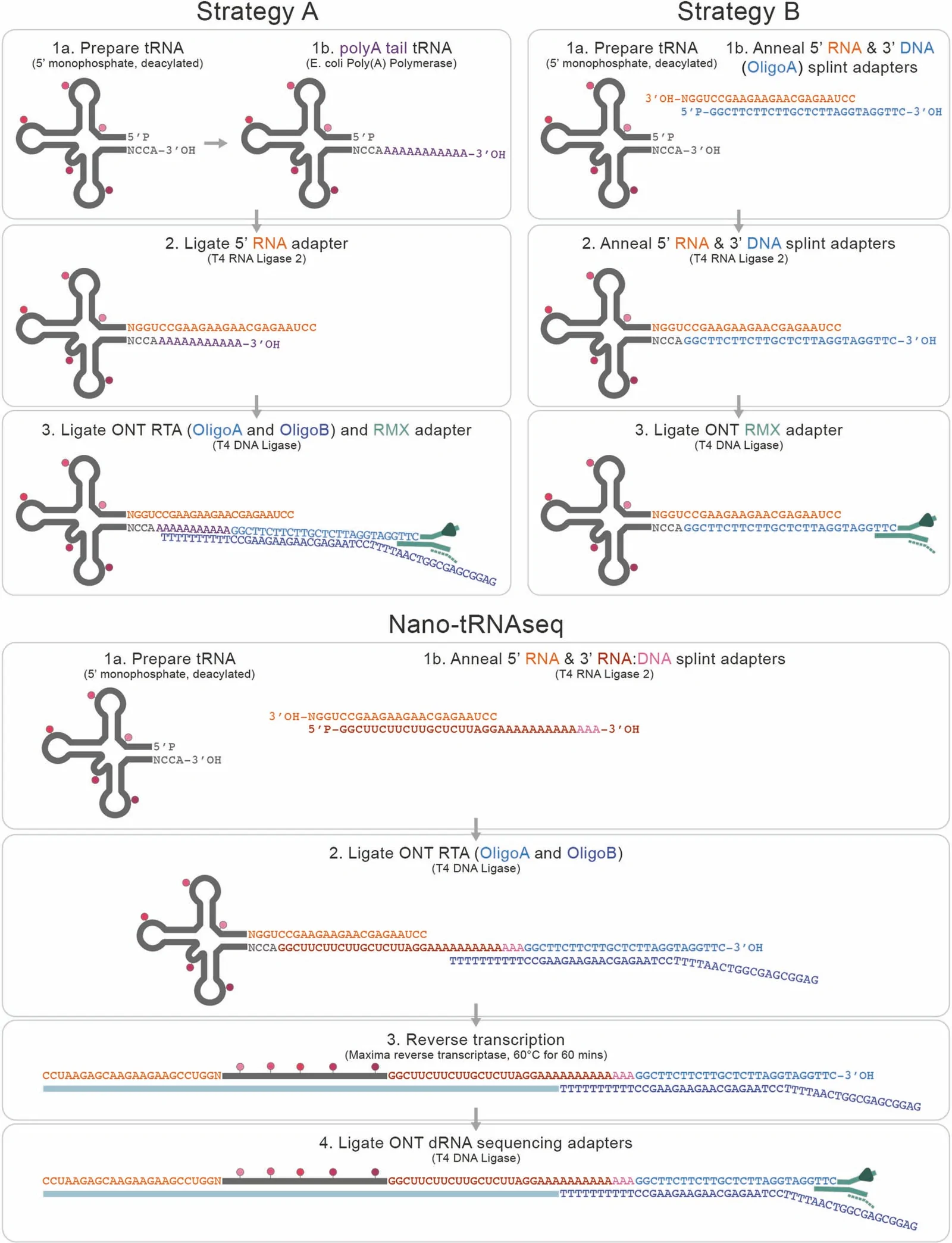
Comparison of the strategies tested to sequence tRNA molecules using nanopore DRS. **(a)** Schematic overview of the three distinct library preparations, Strategy A, Strategy B, and Nano-tRNAseq, tested to sequence tRNA molecules.

We first attempted a modified tRNA DRS approach in which a 5′ RNA adapter, complementary to the 3′ CCA overhang present in mature tRNA molecules, was ligated to the 5′ end of tRNAs that had been previously in vitro polyadenylated (Strategy A; Extended Data Fig. 1 and Methods). A set of nine synthetic in vitro transcribed (IVT) tRNAs of various lengths and sequences (Supplementary Table 1) were sequenced using this strategy. However, this approach produced poor sequencing yields (56,002 reads; Supplementary Table 2) compared to a standard DRS run (~1–2 million reads). Moreover, only 7.5% of reads mapped uniquely to tRNAs using minimap2 (ref. ^100^) with recommended parameters (-ax map -ont -k15) (Supplementary Table 2). Relaxation of the mapping parameters (-ax map-ont -k5), which had previously been shown to improve the mappability of highly modified RNAs^98^, did not significantly increase the number of mapped tRNA reads (Supplementary Table 2).

Next, we altered our library preparation protocol to replace the poly(A) tail with a 3′ DNA adapter complementary to the 5′ RNA adapter (Strategy B), such that the two oligonucleotides could be pre-annealed and ligated to the tRNA (Extended Data Fig. 1). However, this strategy also yielded a low number of sequenced reads (63,502 reads) and a low percentage of uniquely mapped reads (6.5%) (Supplementary Table 2). We speculated that the low number of reads could be due to steric interference of the poly(A) preventing 5′ ligation (Strategy A) or that the 3′ DNA adapter is not basecalled (Strategy B). These scenarios would lead to low coverage of the 5′ or 3′ ends, respectively, decreasing the mappability of reads resulting from these two strategies.

### Extending tRNA ends with RNA adapters improves basecalling

Based on the results of Strategy A and Strategy B, we rationalized that padding the 5′ and 3′ tRNA ends with RNA adapters, which can be accurately basecalled and mapped, would enable us to capture the entirety of the tRNA sequence. This approach, which we termed Nano-tRNAseq (Fig. 1b), was the most successful at sequencing, basecalling and mapping both in vitro and native tRNA molecules using nanopore DRS. We should note that a recent work also proposed a similar solution to facilitate native tRNA nanopore sequencing^92^.

**Extended Data Fig. 2:**
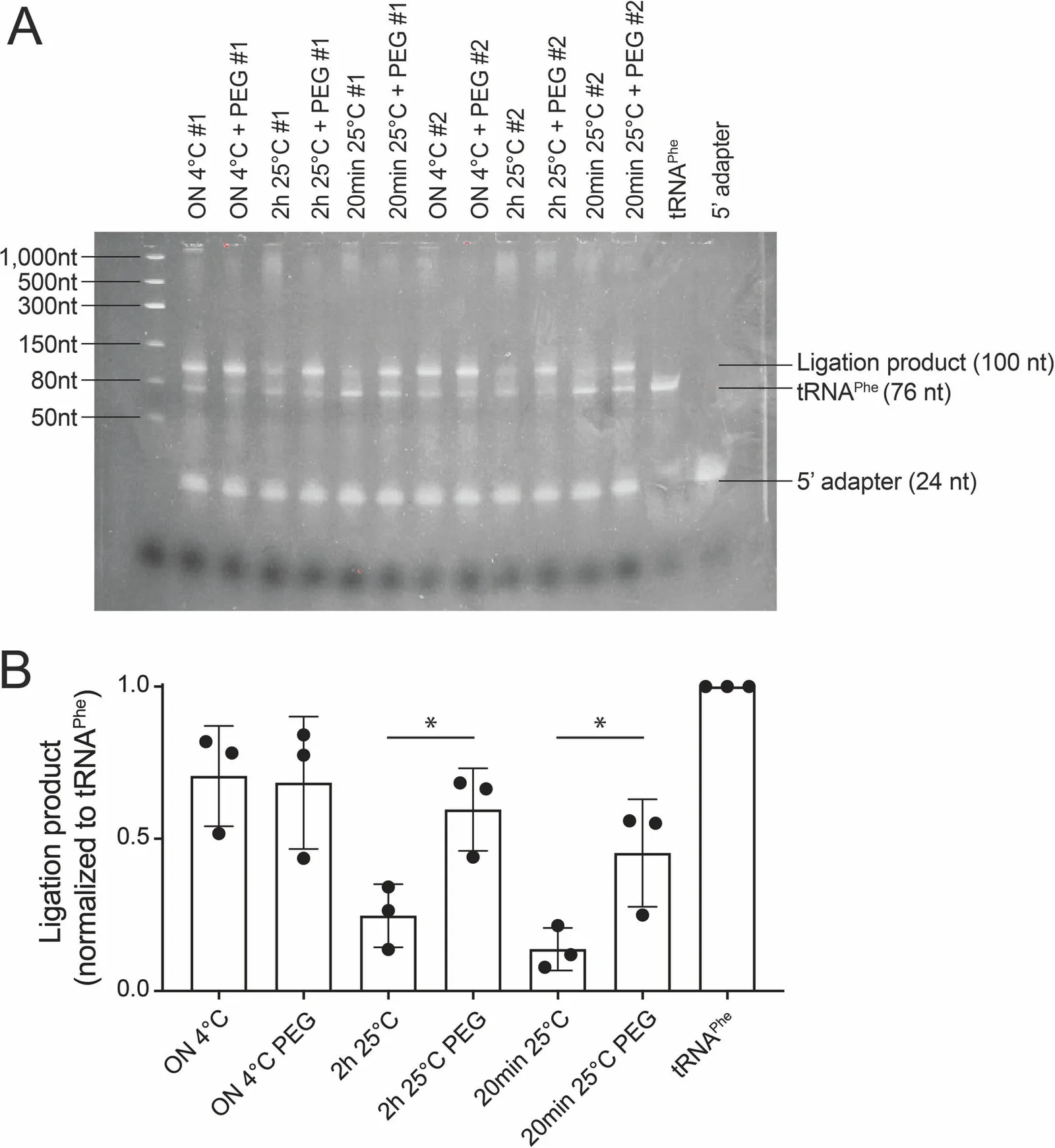
Increased ligation time and addition of PEG8000 improves 5′ RNA adapter ligation efficiency. **(a)** TBE-Urea gels showing the effect of reaction duration and the addition of 20% PEG8000 on ligation efficiency using commercial *S. cerevisiae* tRNA^Phe^ as the ligation template. **(b)** Barplot of the ligation product (tRNA^Phe^ ligated to the 5′ RNA adapter) normalized to an unligated tRNA^Phe^ control. Error bars represent mean ± stdev for *n* = *3* replicates per condition. P values were determined using a two-sided t-test, *P < 0.05, 2 h 25 °C vs 2 h 25 °C p-value = 0.0241, 20 min 25 °C vs 20 min 25 °C p-value = 0.0450. ON = overnight, PEG = PEG8000 (final concentration of 30%).

**Extended Data Fig. 3:**
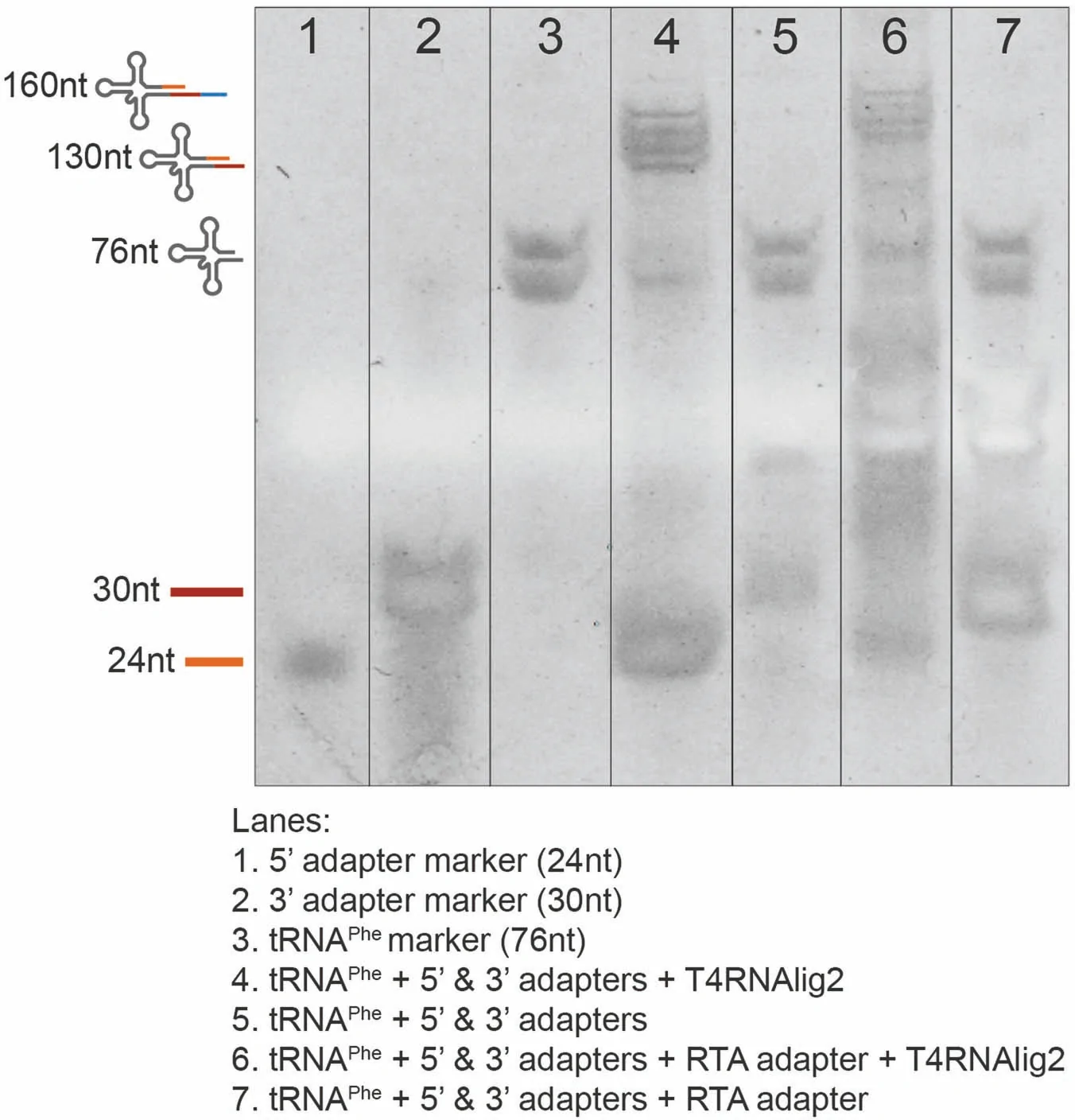
5′ and 3′ RNA oligos can be efficiently ligated to tRNA molecules. TBE-Urea gel of adapter ligation steps used in Nano-tRNAseq, using commercial *S. cerevisiae* tRNA^Phe^ as the ligation template. The lanes are as follows (1) 5′ RNA adapter, (2) 3′ RNA adapter, (3) tRNA^Phe^, (4) tRNA^Phe^ ligated to 5′ and 3′ adapters, (5) tRNA^Phe^ and 5′ and 3′ adapters, without ligase control, (6) tRNA^Phe^ ligated to 5′ and 3′ adapters and RTA adapter, (7) tRNA^Phe^ and 5′ and 3′ adapters and RTA adapter, without ligase control. The experiment was repeated independently twice with similar results.

In the first step, a 5′ RNA adapter (orange) complementary to the CCA overhang of mature tRNAs is pre-annealed to a 3′ RNA adapter (red) containing three DNA bases (pink) at the 3′ end (Fig. 1b). In preliminary Nano-tRNAseq runs, we used an RNA-only 3′ adapter but observed that RNA-only 3′ adapters led to increased self-ligation (Supplementary Table 2), an issue that we mitigated by adding DNA bases to the end of the adapter (Extended Data Fig. 1). Next, the pre-annealed 5′ RNA and 3′ RNA:DNA splint adapters were ligated to deacylated tRNAs. Knowing that an RNA:RNA ligation with an RNA bridge has a low efficiency^101^, various ligation times and the addition of a molecular crowding agent were tested to ensure that conditions that maximized ligation efficiency were chosen (Extended Data Fig. 2a,b). Subsequently, ONT RTA oligoA and oligoB were pre-annealed and ligated to the tRNA molecule using T4 DNA Ligase (see Extended Data Fig. 3 for validation of each ligation step). This approach resulted in >200,000 basecalled reads (Supplementary Table 2), thus significantly increasing sequencing output by up to fourfold relative to the previous strategies, and also with improved 5′ and 3′ coverage of both synthetic and biological tRNAs (Fig. 1c). Additionally, we found Nano-tRNAseq to be highly replicable when sequencing native tRNAs (ρ = 0.984) (Fig. 1d).

### Mapping parameters significantly affect tRNA read mappability

**Fig. 2:**
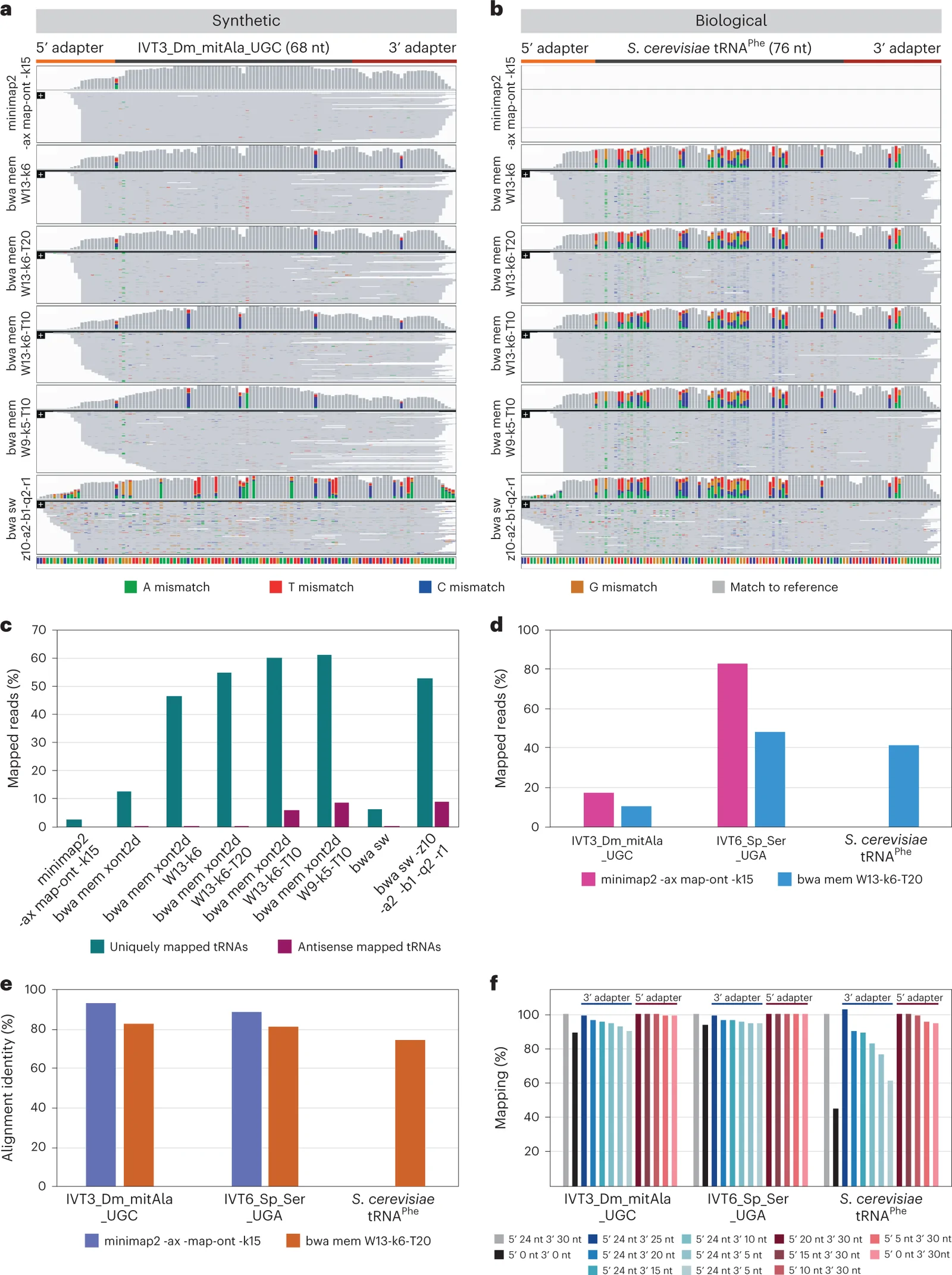
The choice of mapping software and parameters markedly affects the number of mapped tRNA reads. **a**,**b**, IGV snapshots of reads mapped to IVT *D. melanogaster* mitochondrial tRNA^Ala(UGC)^ (**a**) and *S. cerevisiae* tRNA^Phe^ (**b**), mapped using different mapping algorithms (minimap2, BWA-MEM and BWA-SW) and parameters. The 5′ and 3′ RNA adapter regions, ligated to the ends of the tRNA molecule, were included in the mapping references and are represented by an orange bar and a red bar, respectively. Positions with a mismatch frequency greater than 0.2 are colored, whereas those showing mismatch frequencies lower than 0.2 are shown in gray. **c**, Bar plot depicting the effect of algorithm and parameter choice on the relative proportion of uniquely mapped reads (green) and mismapped reads (purple; reads mapping to antisense strands were used as a proxy to assess mismapping) (Supplementary Table 4). The proportion of mapped reads (**d**) and alignment identity (**e**) for each template from the bar plot in **c**, using either minimap2 or bwa mem -W13 -k6- T20. We should note that minimap2 alignment identity in *S. cerevisiae* tRNA^Phe^ was not computed because no reads were mapped to this tRNA using minimap2 with -ax map-ont -k15 parameters (Supplementary Table 5). **f**, Bar plot showing the effect of trimming the length of the 5′ RNA adapter (reds) and 3′ RNA adapter (blues) on tRNA read mappability (Supplementary Table 6). The conditions used by Nano-tRNAseq are gray, whereas the effect of not using RNA adapters is black.

The alignment of native tRNA reads is challenging due to their short and highly modified nature. Indeed, native tRNAs contain a large proportion of mismatched bases, often originating from inaccurate basecalling of modified bases in DRS datasets^94,98,102^. As a consequence of these miscalled bases, the commonly used long-read mapper minimap2 with recommended settings (-ax map-ont -k15) aligned only a fraction (2.56%) of the reads (Fig. 2a–c and Supplementary Table 2). We further tested a range of minimap2 parameters and observed only incremental improvements in mapping and an increase in false alignments (antisense mapped reads served as a proxy of mismapping) (Supplementary Table 3).

To improve the mappability of Nano-tRNAseq reads, we next tested the mapping algorithm BWA, a short-read mapping algorithm commonly used to map Illumina reads^103,104^. Using sequencing data from a Nano-tRNAseq run that contained three different tRNA constructs (IVT *Drosophila melanogaster* mitochondrial tRNA^Ala(UGC)^, IVT *Streptococcus pneumoniae* tRNA^Ser(UGA)^ and native *S. cerevisiae* tRNA^Phe^; see Supplementary Table 2 for a summary of the sequencing runs in this work), we found that the BWA-MEM aligner with recommended parameters outperformed minimap2 in terms of proportion of mapped reads (Fig. 2c), in agreement with recent works^92^. Although more relaxed configurations of BWA-MEM aligned more reads, this also came at the expense of increased false alignments (Fig. 2c and Supplementary Table 4). An optimal balance between increased mapped reads and false alignments was found when using bwa mem with parameters -W13 -k6 -xont2d -T20, which mapped 54.63% of the reads, with very few false alignments (0.19%). When comparing the performance of the mapping algorithms in native tRNA molecules, the contrast was even more stark; although minimap2 mapped IVT tRNAs, it failed to map a single biological tRNA read (Fig. 2d and Supplementary Table 5). The alignment identity was similar to minimap2 for reads that mapped to IVT tRNAs but was slightly lower than the typical identity obtained in nanopore DRS runs, suggesting that short reads, even without modifications, cause a drop in the basecalling accuracy (Fig. 2e). Notably, the alignment identity of *S. cerevisiae* tRNA^Phe^ was lower (~74.5%) than in synthetic tRNAs (81.8%), presumably due to the presence of base modifications present on endogenously modified *S. cerevisiae* tRNA^Phe^ (Fig. 2e and Supplementary Table 5).

We then assessed whether the mappability of Nano-tRNAseq reads might be affected by the length of the 5′ and 3′ RNA adapters. To simulate different RNA adapter lengths, we trimmed one or both adapters from the reference sequences. We found that the absence of both RNA adapters had only a modest effect on the mappability of reads originating from IVT tRNAs (decrease of 6–11%) (Fig. 2f and Supplementary Table 6), whereas their absence had a major effect in the mappability of native *S. cerevisiae* tRNA^Phe^ (55% loss). Thus, we concluded that short and unmodified sequences can be aligned efficiently even without RNA adapters in the reference sequences, whereas short and modified reads greatly benefit from the extension with adapters, demonstrating that extending molecules with RNA adapters is essential for guiding the correct alignment of short reads enriched in ‘mismatches’, such as those derived from native tRNAs. For native *S. cerevisiae* tRNA^Phe^, the read mappability plateaued at a 3′ adapter length of 25 nt (Fig. 2f), suggesting that the 30-nt RNA portion of the 3′ RNA:DNA oligo used in Nano-tRNAseq is more than sufficient to achieve optimal read mappability.

### Custom MinKNOW improves yield and tRNA abundance estimates

A surprising feature of our initial tRNA sequencing runs was the low amount of sequenced reads. Although pore clogging caused by tRNA structure might partially explain the low sequencing yield^92^, we also noticed that the MinKNOW software classified a high proportion of reads as ‘adapter-only’ reads in real time. Hence, we hypothesized that a considerable fraction of tRNA reads might be discarded by the MinKNOW software due to their short signal lengths, as they resemble ‘adapter-only’ reads.

**Fig. 3:**
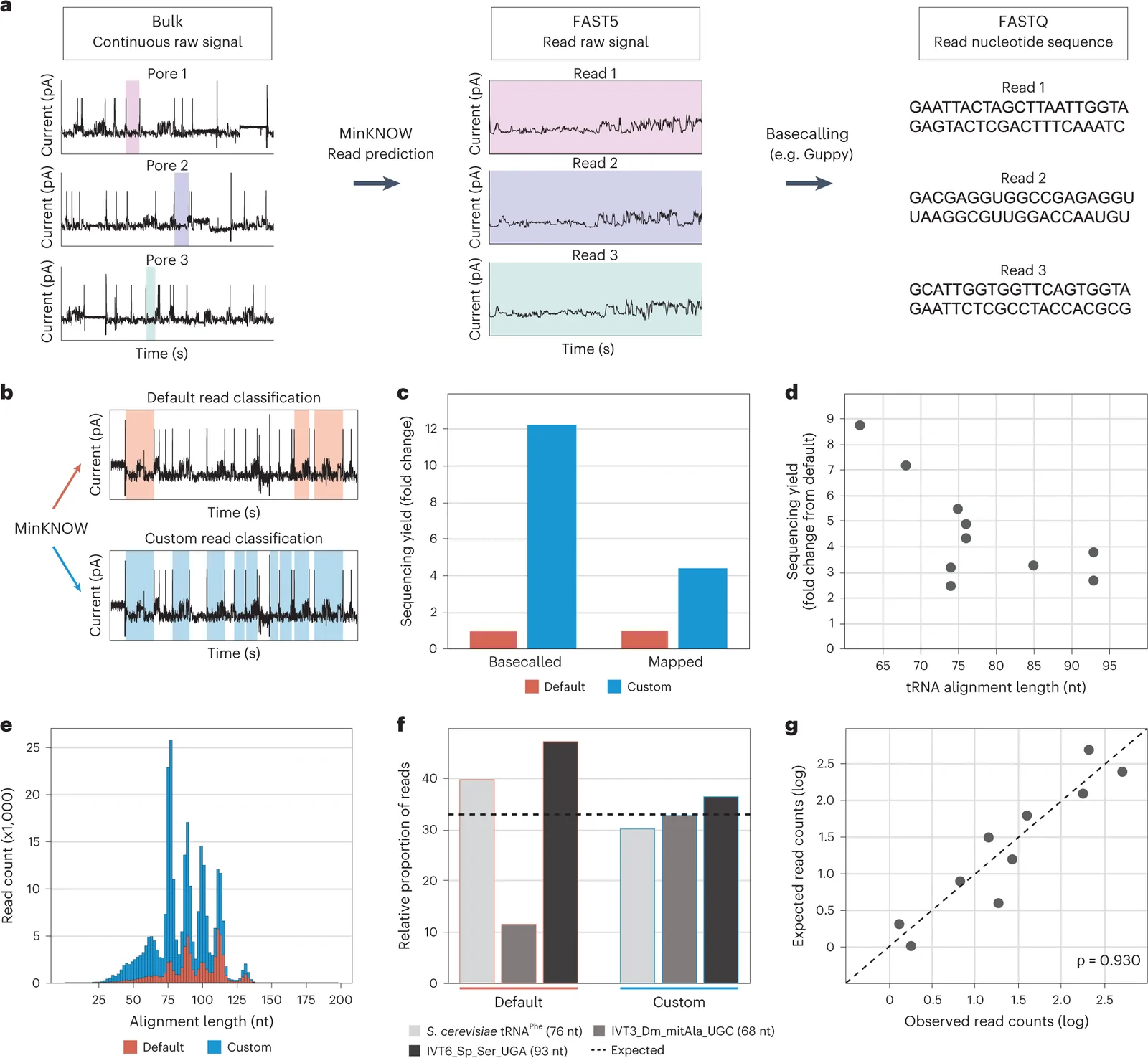
Adjustment of MinKNOW parameters increases the number of sequenced and mapped tRNA reads. **a**, MinKNOW software classifies continuous current passing through pores as open pore, adapter or strand (actual reads) and outputs fragments classified as strand to a FAST5 file, which are then basecalled to generate a FASTQ file. **b**, Diagram showing the conceptual difference between default and custom MinKNOW read classification (Extended Data Fig. 4). **c**, Bar plot of sequencing yield in terms of basecalled and uniquely mapped reads obtained with default and custom configurations (Supplementary Table 7). **d**, Scatter plot of the relative fold change of uniquely mapped reads with respect to tRNA length (Supplementary Table 8). **e**, Histogram of read count and alignment length of IVT tRNA reads captured with default and custom configurations. **f**, Bar plot of the relative proportion of IVT tRNA molecules *D. melanogaste*r mitochondrial tRNA^Ala(UGC)^ and *S. pneumoniae* tRNA^Ser(UGA)^ and native *S. cerevisiae* tRNA^Phe^ reads recovered with default and custom settings (Supplementary Table 9), where the dotted line indicates the expected proportion. **g**, Expected versus observed log read counts of nine IVT and one native tRNA molecules captured using the custom MinKNOW configuration (Supplementary Table 10). Spearman correlation (ρ) is shown.

**Extended Data Fig. 4:**
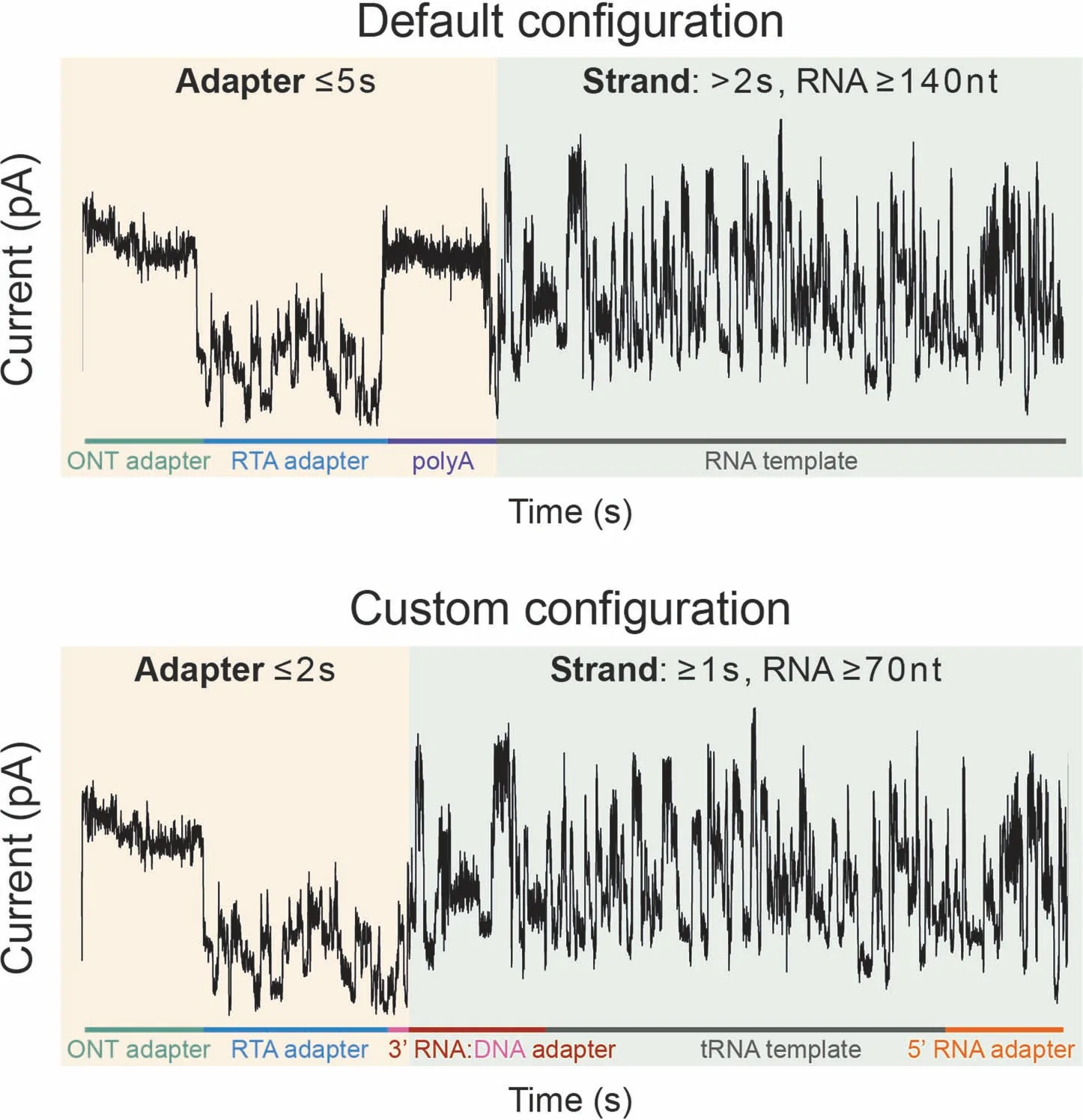
Schematic of default and custom MinKNOW read classification. Under default settings, sequenced templates are classified as reads and if the *Adapter* portion, which contains the ONT adapter, RTA adapter, and polyA tail, is 5 seconds or less, and the *Strand* portion, which contains the RNA template, is more than 2 seconds, which corresponds to an RNA molecule of roughly 140 nt. For Nano-tRNAseq, we use a custom configuration in which the *Adapter* portion, which contains the ONT adapter, RTA adapter, and the DNA portion of the 3′ RNA:DNA adapter, is 2 seconds or less, and the *Strand* portion, which contains the RNA portion of the 3′ RNA:DNA adapter, the tRNA template, and the 5’ RNA adapter, is 1 second or more, which corresponds to an RNA molecule of roughly 70 nt.

The MinKNOW software is responsible for analyzing the continuous electrical current (signal intensity) measured at each pore, reporting the signal regions that correspond to ‘reads’ into FAST5 files, which are then basecalled to generate a FASTQ file (Fig. 3a). We noted that MinKNOW, by default, reports reads that last at least 2 seconds, roughly corresponding to RNA molecules of 140 nt (assuming constant helicase processivity of 70 nt per second in DRS) (Extended Data Fig. 4). Considering that canonical tRNA molecules are ~73 nt, this would imply that even after double RNA adapter ligation (where 24 and 30 RNA nucleotides are added to the 5′ and 3′ ends of the tRNA molecule, respectively), the size of the ligated tRNA molecule would still be below the threshold, possibly leading to misassignment of tRNA reads as ‘adapter-only’ reads. To alleviate this issue, we tested whether alternative MinKNOW configurations would improve the classification of tRNA reads and boost sequencing yields. To this end, the bulk ‘raw’ dump files were saved during the sequencing and were reprocessed using alternative MinKNOW configurations (Supplementary Table 7).

By lowering the MinKNOW strand minimum duration to 1 second and the adapter maximum duration to 2 seconds (see Extended Data Fig. 4 for schematic), a configuration that we refer to as *custom*, we captured ~12-fold more basecalled and ~4.5-fold more uniquely mapped tRNA reads compared to the default MinKNOW configuration (Fig. 3b,c and Supplementary Table 7). Notably, we found that the default MinKNOW configuration not only led to low sequencing outputs but also caused significant biases in the relative abundances of tRNA molecules. Specifically, we found a greater representation of shorter tRNAs in our custom configuration (Fig. 3d,e and Supplementary Table 8), suggesting that the default MinKNOW configuration is discarding shorter tRNA molecules and preferentially capturing longer ones, such as tRNA molecules with variable arms (for example, tRNA^Leu^, tRNA^Arg^ and tRNA^Ser^). Moreover, the relative proportion of tRNA reads was better recapitulated using the custom configuration than default settings (Fig. 3f), and the reported tRNA abundances using custom settings correlated well to the expected values (ρ = 0.93) (Fig. 3g and Supplementary Tables 9 and 10).

### Reverse transcription of tRNAs increases sequencing yield

**Fig. 4:**
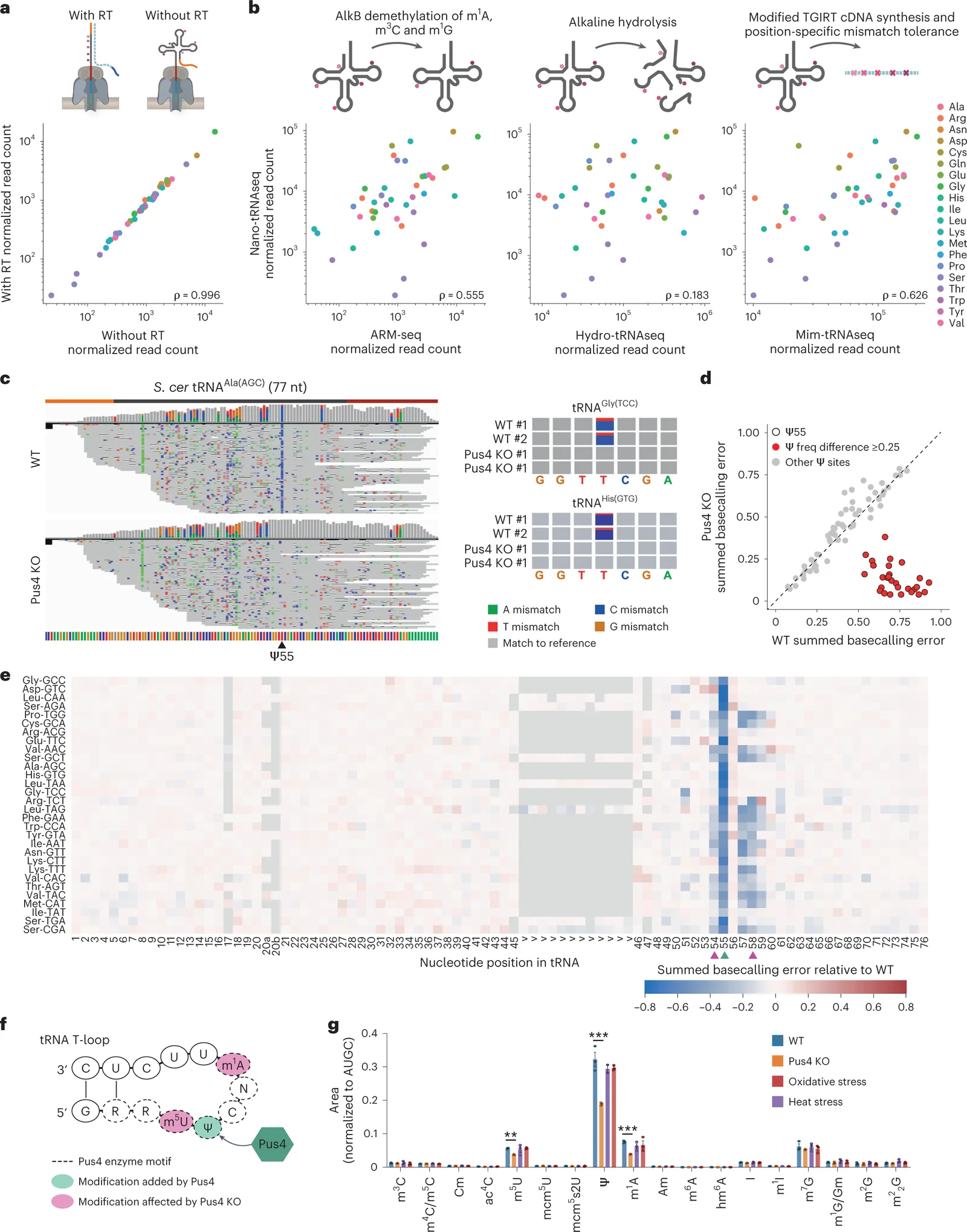
Nano-tRNAseq can quantify tRNA abundance and RNA modifications as well as capture modification interdependencies. **a**,**b**, Scatter plots of WT *S. cerevisiae* tRNA abundances sequenced with Nano-tRNAseq with and without the reverse transcription step (**a**) and compared to orthogonal Illumina-based tRNA sequencing methods (**b**). Each point represents a tRNA alloacceptor and is colored by alloacceptor type; the key is shown in **b**. The correlation strength is indicated by Spearman’s correlation coefficient (ρ). **c**, IGV tracks of tRNA^Ala(AGC)^ from WT and Pus4 KO *S. cerevisiae* strains (*n* = *2* biological replicates). Ψ55 is indicated with a black arrowhead. Adjacent are zoomed IGV snapshots of the Ψ55 region. Positions with a mismatch frequency greater than 0.2 are colored, whereas those lower than 0.2 are shown in gray. **d**, Scatter plot showing the mismatch frequencies for Ψ sites in *S. cerevisiae* WT versus Pus4 KO tRNA molecules. Each data point represents a known tRNA Ψ site; a black outline indicates Ψ55 sites; and a red fill indicates sites with a summed basecalling error of ≥0.25 compared to WT. **e**, Heat map of the summed basecalling error of Pus4 KO relative to WT, for each nucleotide (*x* axis) and for each tRNA isoacceptor (*y* axis, ordered from most to least abundant in descending order)(Supplementary Table 17). The positions of known tRNA modifications found in each tRNA gene are listed in Supplementary Table 18. The Pus4 target Ψ55 is indicated with a green arrowhead and m^5^U54 and m^1^A58 with pink arrowheads. See also Extended Data Fig. 9a for biological replicate 2. **f**, Schematic of the tRNA T-loop targeted by the Pus4 enzyme. Nucleotides with a dotted outline represent the Pus4 binding motif (RRUUCNA); Ψ55 is highlighted in green; and m^5^U54 and m^1^A58 are highlighted in pink. **g**, LC–MS/MS validation of *S. cerevisiae* tRNA modification levels. See also Supplementary Tables 19 and 20. Bars represent mean ± s.e.m. for *n* = 3 biological replicates per condition. *P* values were determined using a one-way ANOVA with Tukey correction for multiple comparisons, and significance was assessed by comparison to WT. *P < 0.05, **P < 0.01, ***P < 0.001, *P*(m^5^U) = 0.0015, *P*(Ψ) and *P*(m^1^A) < 0.0001. RT, reverse transcription.

**Extended Data Fig. 5:**
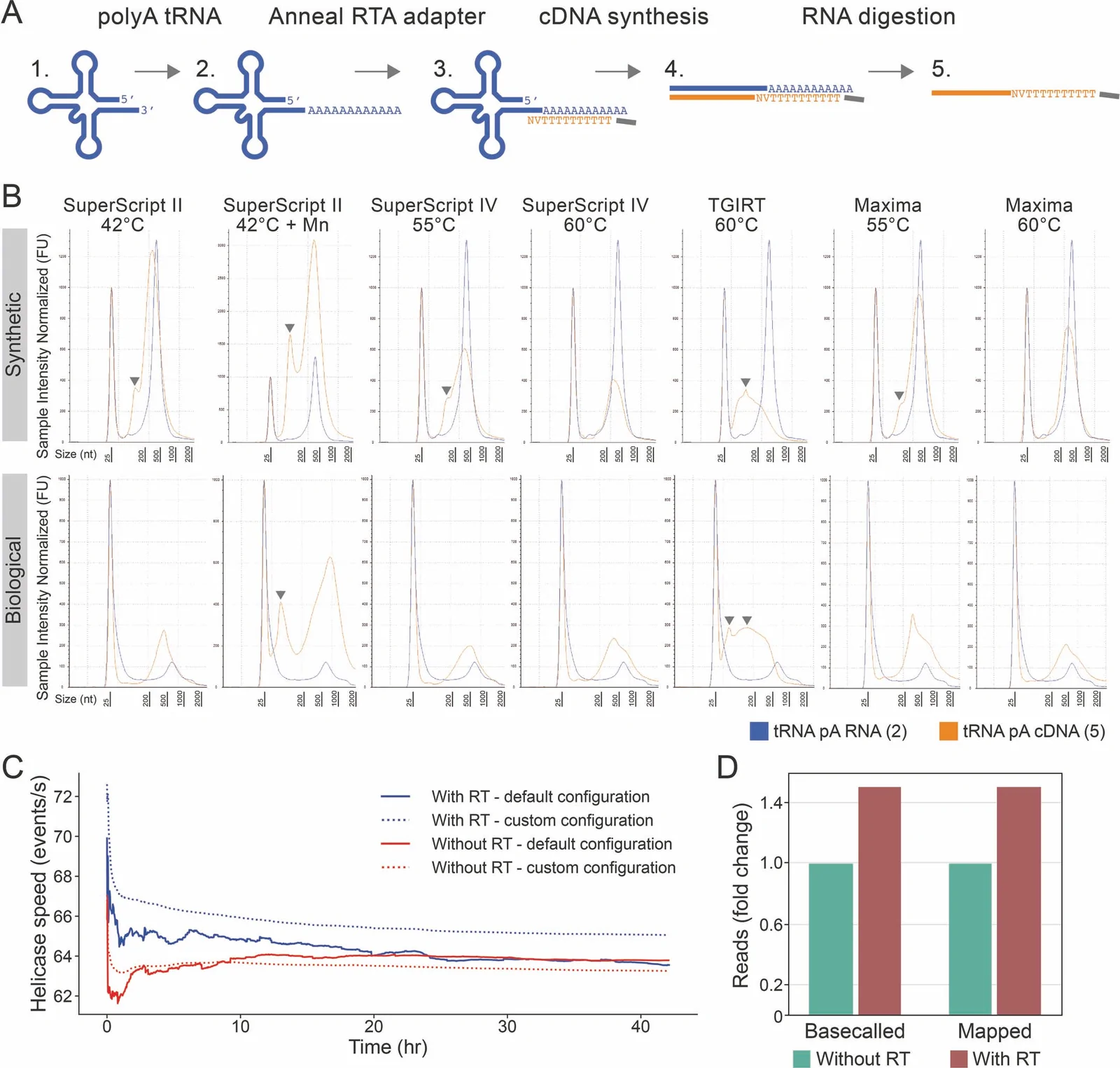
Comparison of the activity of diverse reverse transcriptase enzymes for tRNA linearization. (**a**) The strategy used to test the reverse transcription activity of different enzymes. Starting from either in vitro transcribed (IVT) or native tRNA (1), the tRNA was polyadenylated (2) and annealed with an oligodT adapter (3), which was used to initiate the cDNA synthesis using different RT enzymes and conditions. The RNA strand of the linearized product (4) was digested, leaving the cDNA strand (5), which was checked via TapeStation. **(b)** TapeStation profiles depicting the original polyA (pA) tRNA product (blue) and the cDNA product (orange) that is produced by reverse transcription of the template using diverse reverse transcriptases and incubation conditions. Truncated cDNA products are shown with a gray triangle. The 25 nt peak that is present in all samples corresponds to the loading size marker. The upper panel is IVT tRNA, and the lower panel is commercial *S. cerevisiae* tRNA^Phe^. **(c)** Helicase speed (events/s roughly corresponds to nt/s sequenced) over time of wild-type (WT) *S. cerevisiae* total tRNA sequenced with or without reverse transcription (RT) and classified using the default or custom MinKNOW configuration. **(d)** Barplot showing the fold change of basecalled and uniquely mapped reads when WT *S. cerevisiae* total tRNA is linearized with reverse transcription, compared to without reverse transcription.

We next questioned whether removing the tRNA structure, which can be achieved via linearization by reverse transcription, would further improve our sequencing yield. We should highlight that, in the case of DRS, the native RNA molecule is sequenced, whereas the cDNA strand is not (see Fig. 4a schematic). A linear tRNA molecule may (1) reduce the clogging of pores, allowing more reads to be sequenced, and maintain the integrity of the flowcell longer and/or (2) stabilize the tRNA translocation speed through the pore, improving the accuracy of basecalling algorithms. Notably, tRNAs are notoriously difficult to fully and accurately reverse transcribe due to their compact secondary and tertiary structures as well as their abundance of modifications that disrupt the Watson–Crick base pairing^74,80,86^ (Fig. 1a). To examine whether tRNA linearization might improve sequencing yield, we tested a range of commercial reverse transcriptases and incubation conditions on both IVT and native tRNAs and examined their cDNA outputs using TapeStation (Extended Data Fig. 5a,b and Methods). We found that both Maxima and SuperScript IV at 60 °C offered the best performance in the production of full-length cDNA products, and we opted to use Maxima at 60 °C in our subsequent tRNA sequencing experiments (Extended Data Fig. 5b).

Next, we examined whether linearization of the tRNAs would increase our sequencing yields. To this end, total tRNA from *S. cerevisiae* was sequenced using Nano-tRNAseq with and without the reverse transcription step. The default MinKNOW configuration *without reverse transcription* condition resulted in more reads compared to the *with reverse transcription* condition (Supplementary Table 2). We found that non-linearized tRNAs, which are more structured than linearized ones, caused the helicase enzyme to process these molecules more slowly (Extended Data Fig. 5c), possibly increasing the likelihood that they are classified as a ‘read’ by the default MinKNOW configuration (see Extended Data Fig. 4 for a schematic of read classification). Using the custom MinKNOW configuration (Fig. 3b–e), the number of basecalled reads *with reverse transcription* was 1.5-fold higher compared to *without reverse transcription* (Extended Data Fig. 5d and Supplementary Table 11). Likewise, the number of reads uniquely mapped to tRNAs increased by 1.5-fold with reverse transcription, and the relative abundance of tRNA isoacceptors was not affected by the linearization step (Fig. 4a; ρ = 0.996). Overall, linearization of tRNA molecules improved the sequencing yields by increasing the helicase translocation rate, and, therefore, the reverse transcription step was included in all subsequent Nano-tRNAseq library preparations.

### Nano-tRNAseq correlates with Illumina-based methods

**Extended Data Fig. 6:**
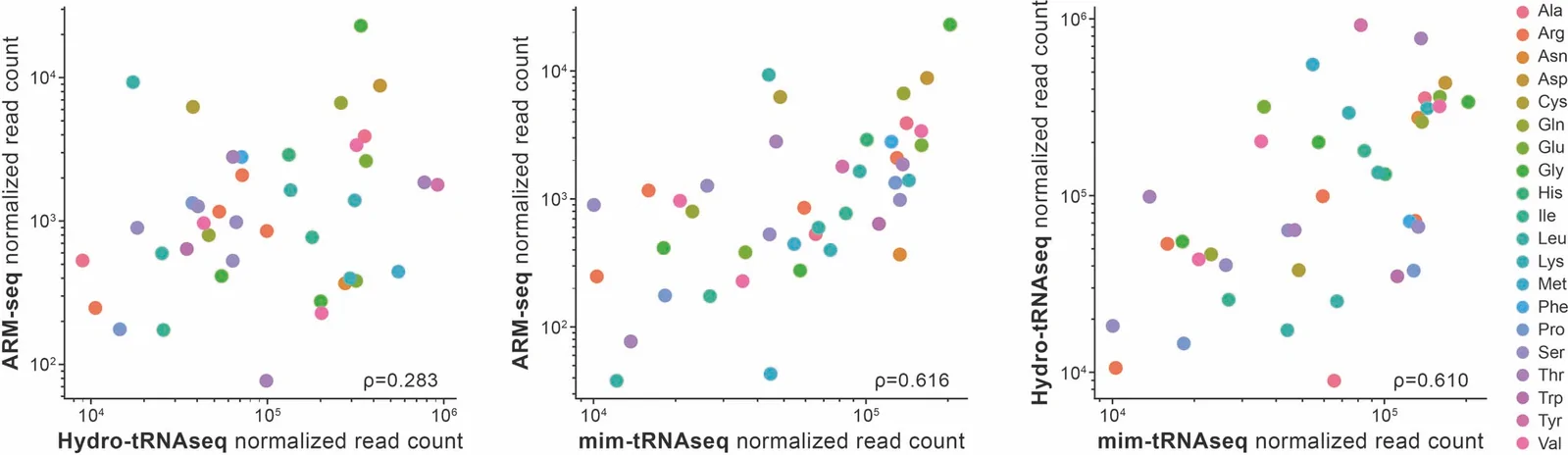
Comparison of the Illumina-based methods to each other. Scatterplots comparing Illumina-based tRNA sequencing methods ARM-seq, Hydro-tRNAseq, and mim-tRNAseq, to each other when sequencing wild-type *S. cerevisiae* total tRNA. Each point represents a tRNA alloacceptor and is colored based on alloacceptor type. The correlation strength is indicated by Spearman’s correlation coefficient (ρ).

Our results show that Nano-tRNAseq, when used with optimized mapping settings and custom MinKNOW configuration, resulted in observed tRNA abundances highly similar to the expected values (Fig. 3g; ρ = 0.93). We, therefore, wondered whether tRNA abundances predicted using Nano-tRNAseq would correlate well with Illumina-based approaches. To this end, we compared Nano-tRNAseq *S. cerevisiae* tRNA abundances to those reported using three different Illumina-based methods: (1) ARM-seq^74^, (2) Hydro-tRNAseq^80^ and (3) mim-tRNAseq^77^. In ARM-seq, tRNAs are pre-treated with demethylating enzyme *Escherichia coli* AlkB, which removes m^1^A, m^3^C and a fraction of m^1^G modifications. Hydro-tRNAseq relies on partial alkaline RNA hydrolysis that generates fragments amenable for sequencing. In the case of mim-tRNAseq, the authors improved the efficiency of cDNA synthesis by optimizing TGIRT reverse transcription conditions and allowing for position-specific mismatch tolerance during read alignment. Nano-tRNAseq correlated best with the Illumina-based methods that address the presence of reverse-transcription-truncating modifications, namely ARM-seq (ρ = 0.555) and mim-tRNAseq (ρ = 0.626), and worst with Hydro-tRNAseq (ρ = 0.182) (Fig. 4b). The low correlation with Hydro-tRNAseq is probably due to the fact that (1) fragments that harbor such modifications are especially short and less likely to be PCR amplified and (2) mapping fragmented samples is challenging and can lead to spurious tRNA counts. Overall, the generally low correlation of Illumina-based methods with Nano-tRNAseq is unsurprising given the substantial differences in library preparation and analysis as well as potential differences in yeast culturing conditions between laboratories. We should note that Illumina-based tRNA sequencing methods showed only modest correlations with each other (ρ = 0.283–0.616) (Extended Data Fig. 6).

### Nano-tRNAseq can quantify tRNA modification differences

**Extended Data Fig. 7:**
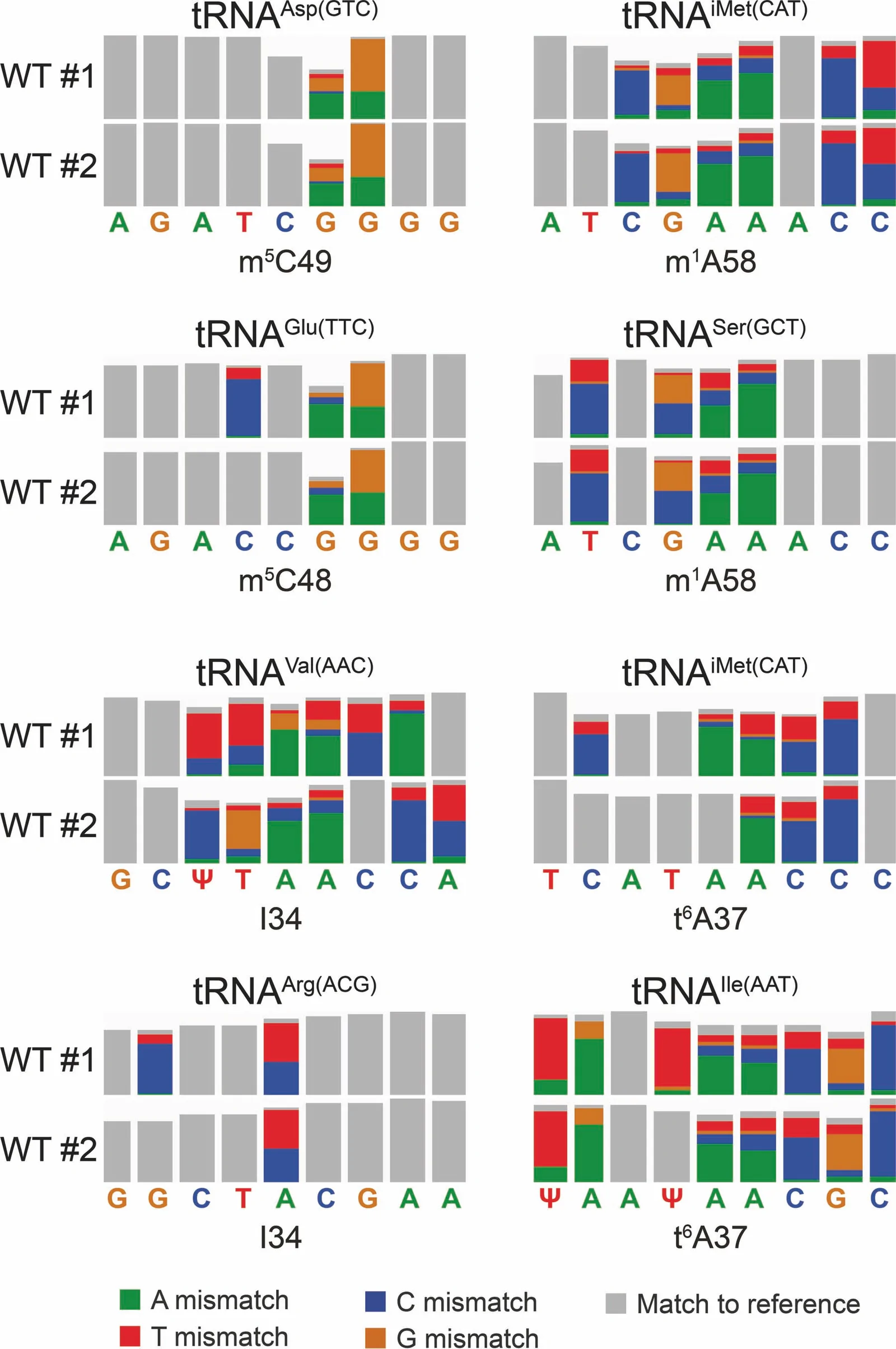
RNA modification signatures observed in Nano-tRNAseq datasets span multiple bases and are sequence-dependent. Zoomed snapshots of WT *S. cerevisiae* Nano-tRNAseq runs, highlighting the signatures at m^5^C, m^1^A, I, and t^6^A modified sites. The upper row corresponds to biological replicate 2, and the lower row corresponds to biological replicate 2. Positions with a mismatch frequency greater than 0.2 are colored, whereas those showing mismatch frequencies lower than 0.2 are shown in gray.

Previous works have shown that basecalling errors, or mismatches to the reference, can be used to detect RNA modifications^94,98,102,105–109^. In agreement with these observations, biological *S. cerevisiae* tRNA^Phe^ showed considerably more mismatch errors than those seen in synthetic IVT tRNAs (Fig. 1d). On closer inspection, the position of many of these mismatches largely coincided with known RNA modifications, some of which affect the basecalled features with single-base resolution, such as Ψ, whereas others influence the signal of neighboring bases, such as m^1^A (Extended Data Fig. 7), in agreement with previous observations^98^.

**Extended Data Fig. 8:**
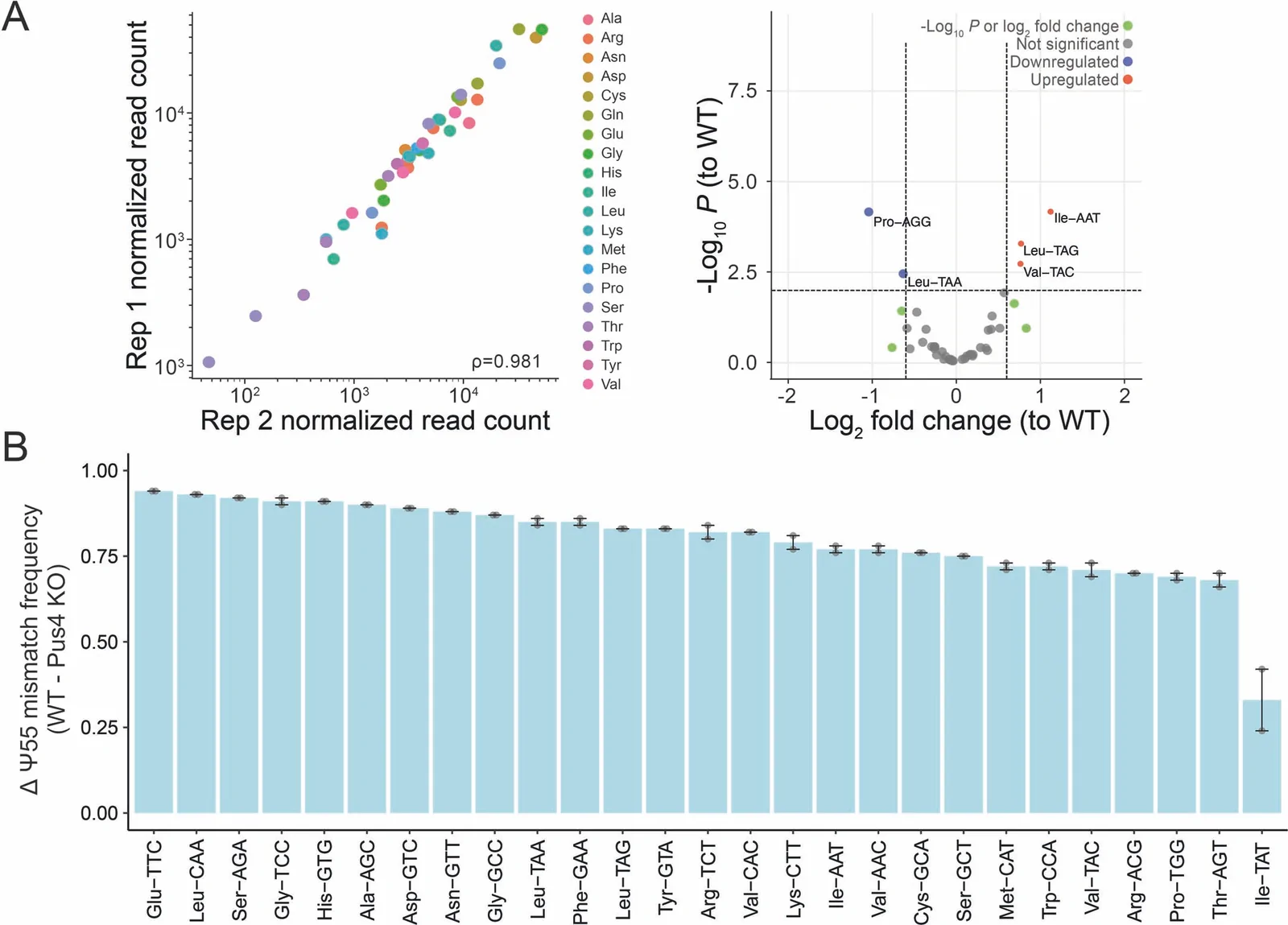
tRNA abundance and changes in RNA modification stoichiometry can be quantified using Nano-tRNAseq. **(a)** Scatterplots showing tRNA abundances of *S. cerevisiae* Pus4 knockout (KO) across biological replicates. See also Supplementary Table 21. Each point represents a tRNA alloacceptor and is colored based on alloacceptor type. The correlation strength is indicated by Spearman’s correlation coefficient (ρ Differential expression volcano plot of pus4KO versus WT (see also Supplementary Table 13). Differentially expressed tRNAs were defined as having an adjusted -log_10_ P-value of <0.01 and an absolute log_2_ fold change greater than 0.6. **(b)** Change in Ψ55 mismatch frequency upon knockout of Pus4, relative to WT, for each isoacceptor, as calculated by NanoRMS. Data are presented as mean ± SEM for *n* = 2 biological replicates.

To confirm whether the basecalling ‘errors’ observed in native tRNAs were indeed the result of RNA modifications, we sequenced tRNAs from wild-type (WT) and a Pus4-deficient *S. cerevisiae* strain. Pus4 is an enzyme responsible for synthesizing Ψ55 from U55 in the T-loop of tRNAs^110^. Upon knockout of Pus4, we observed a striking loss of the characteristic U-to-C mismatch of Ψ^98,111,112^ at position 55 in all tRNAs, whereas other known Ψ sites, which are not reported to be catalyzed by Pus4, were unaffected (Fig. 4c,d and Supplementary Table 12). Despite the loss of Ψ55 in Pus4-deficient *S. cerevisiae*, we observed only modest changes in tRNA isoacceptor levels (Extended Data Fig. 8a and Supplementary Table 13). Using NanoRMS, a tool that we previously developed for quantifying RNA modification stoichiometry in ONT DRS data and validated for Ψ modifications^98^, we calculated the change in Ψ55 stoichiometry. As expected, upon knockout of Pus4, we observed a change in stoichiometry between 68% and 93%, with the exception of Ile-TAT (33%), potentially due to low coverage (Extended Data Fig. 8b and Supplementary Table 14).

**Extended Data Fig. 9:**
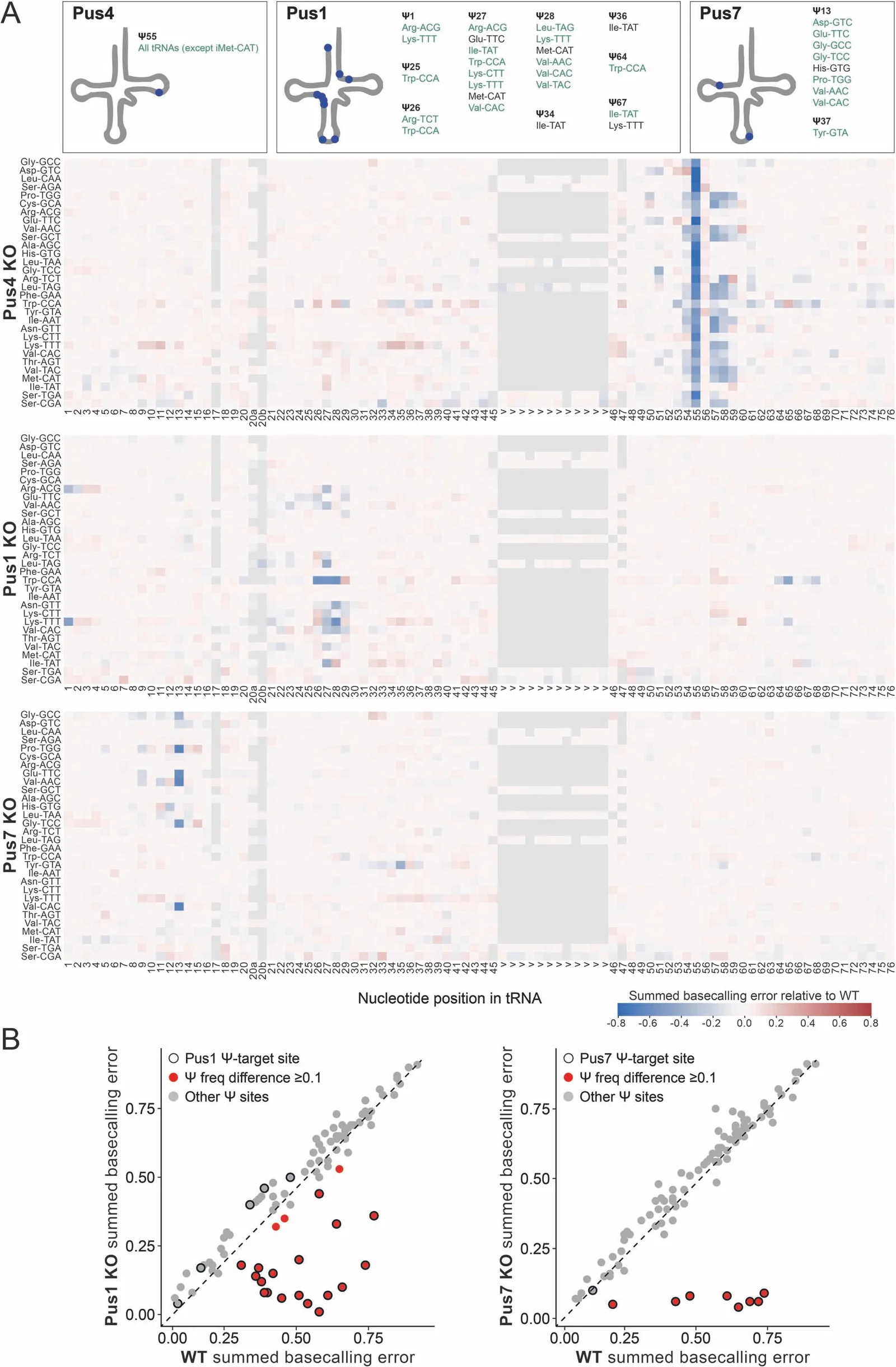
Nano-tRNAseq can capture RNA modifications changes upon knockout of pseudouridine synthase enzymes. **(a)** Heatmap of summed basecalling error frequency of Pus4 KO biological replicate 2, Pus1 KO, and Pus7 KO (see also Supplementary Table 16 and Supplementary Table 17). The known positions of specific RNA modifications in each tRNA, as listed in MODOMICS, are shown in schematic above, as well as listed in Supplementary Table 18. Ψ positions observed in Nano-tRNAseq are highlighted in green (greater or equal to 0.1, see Supplementary Table 15). Nucleotides with a higher summed basecalling error frequency relative to WT are in red tones, and those with a lower summed basecalling error frequency are in blue tones. **(b)** Comparison of mismatch frequencies for known Ψ sites in S. cerevisiae WT vs. Pus1 and Pus7 KO tRNA molecules. Each data point represents a known tRNA Ψ site; a black outline indicates Ψ55 sites targeted by the enzyme in question, and a red fill indicates sites with a summed basecalling error of ≥0.1 for Pus 1 KO and Pus 7 KO compared to WT, which serves as a proxy for Ψ modification frequency.

Similarly, we also sequenced tRNAs from Pus1-deficient and Pus7-deficient *S. cerevisiae* strains. Pus1 is a multi-site Ψ synthase that modifies tRNA at positions 1, 26–28, 34, 36, 65 and 67 (refs. ^113–115^), whereas Pus7 catalyzes pseudouridylation at position 13 in a subset of tRNAs^116^ (Supplementary Table 15). In both cases, we observed a loss of Ψ in most annotated Ψ sites upon Pus1 or Pus7 depletion (Extended Data Fig. 9a,b and Supplementary Tables 15 and 16) using Nano-tRNAseq. We should note that, in the case of Glu-TTC, the Ψ27 position appears to be shifted by −1 nt, as is Ψ28 in Leu-TAG (Extended Data Fig. 9a). In this work, we used annotated modified positions listed in MODOMICS as our reference list (Supplementary Table 18), which we manually curated using previously published literature^3,25,117^. We think that these positions are shifted by −1 nt because it occurs at canonical position 26 and 27 in the Glu-TTC and Leu-TAG, respectively, rather than a shift in the basecalling error of Ψ, which typically produces a basecalling error at the expected base (the modified site)^98,111^.

### Nano-tRNAseq identifies tRNA modification interdependencies

**Extended Data Fig. 10:**
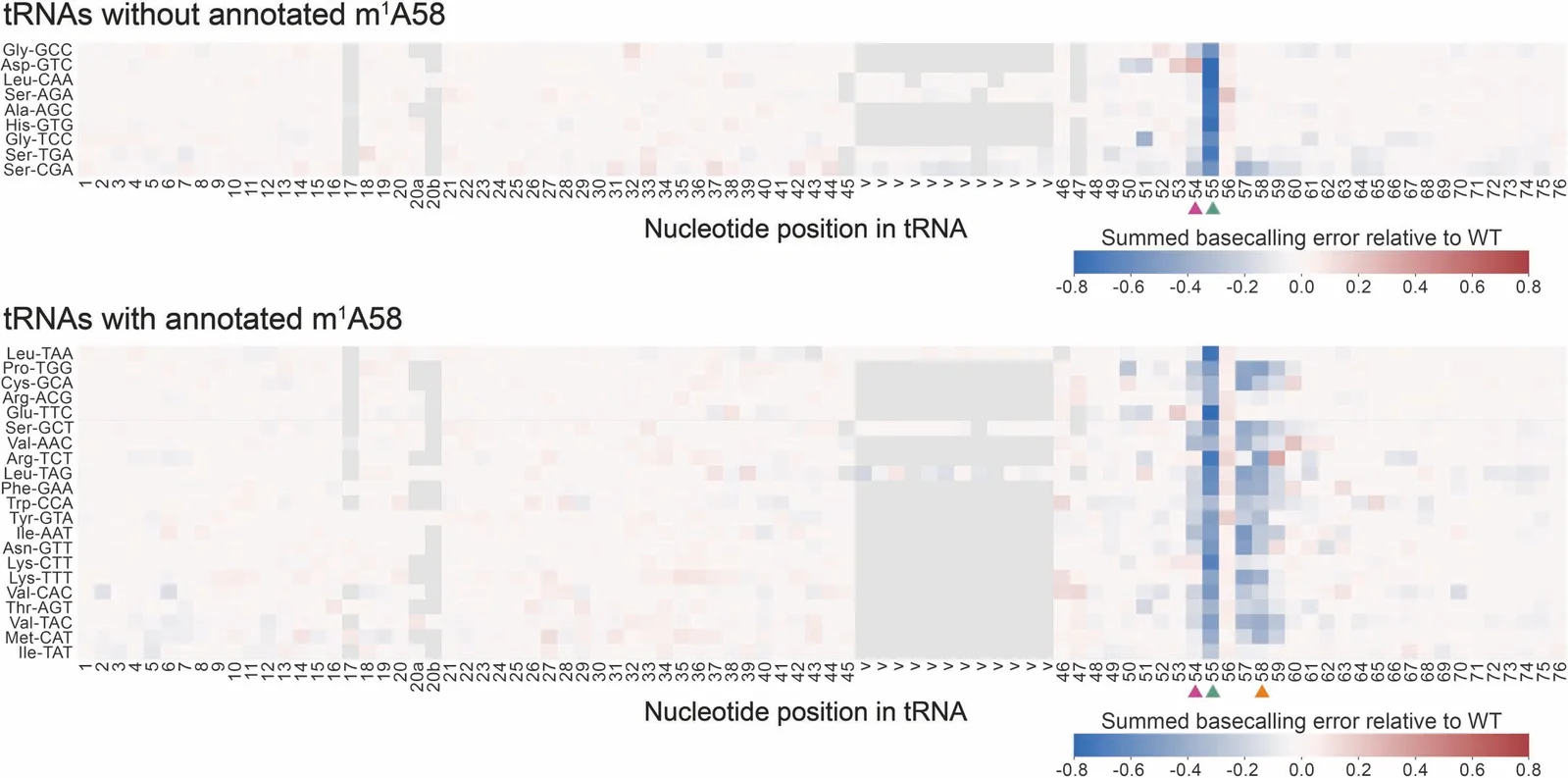
Ψ55-dependent basecalling error is restricted to position 55 independent of m^1^A58 presence. Heatmap of summed basecalling error frequency of Pus4 KO biological replicate 1 (**as in** Fig. 4e) categorized by tRNA isoacceptors without an annotated m^1^A58 (upper panel) or those with an annotated m^1^A58 (lower panel). The positions of specific RNA modifications in each tRNA are listed in Supplementary Table 18. Nucleotides with higher summed basecalling error frequency relative to WT are in red tones, and those with a lower basecalling error frequency are in blue tones. Ψ55 is indicated by a green arrowhead, m^5^U54 by a pink arrowhead, and m^1^A58 by an orange arrowhead.

tRNA modifications are introduced in a defined sequential order, and the chronology is controlled by the crosstalk between modification events and RNA-modifying enzymes^118,119^. Using time-resolved nuclear magnetic resonance (NMR) monitoring of tRNA maturation, Barraud et al.^120^ reported a robust modification hierarchy in the T-loop of *S. cerevisiae* tRNA^Phe^, with Ψ55 positively influencing the introduction of both m^5^U54 and m^1^A58, and m^5^U54 positively influencing the introduction of m^1^A58. To explore whether our method could capture the effect of Ψ55 loss on other modifications, we examined the summed basecalling error (base mismatch, insertion and deletion) for each nucleotide position and tRNA molecule reference, comparing the tRNA modification profiles of each tRNA isoacceptor in Pus4 knockout (KO) strains relative to WT (Fig. 4e, Extended Data Fig. 9a and Supplementary Table 17; see Supplementary Table 18 for a summary of all *S. cerevisiae* annotated RNA modifications and their positions). In addition to the decrease in basecalling error at position 55 (corresponding to the loss of the modification), we observed a decrease also at positions 54 and 57–59 (Fig. 4e,f), depending on the tRNA isoacceptor. LC–MS/MS of the same samples confirmed that there was a reduction in m^5^U and m^1^A modification levels, further supporting that the incorporation of m^1^A58 and m^5^U54 depends on the presence of Ψ55 (Fig. 4g, Extended Data Fig. 10 and Supplementary Tables 19 and 20).

As an orthogonal validation, we analyzed HydraPsiSeq data from *S. cerevisiae* WT and Pus4 mutant strains generated in a previously published study^121^. HydraPsiSeq is an NGS-based quantitative Ψ mapping technique relying on specific protection from hydrazine/aniline cleavage, where U residues are sensitive to hydrazine and, thus, efficiently cleaved. In contrast, Ψ residues are resistant and provide only background signals (Supplementary Fig. 1a). In the resulting Integrative Genomics Viewer (IGV) tracks, loss of Ψ is represented by a dropoff, and we observe that the m^1^A58 mismatch error is significantly reduced in the Pus4 KO condition relative to WT (Supplementary Fig. 1b,d) in some of the isoacceptors. We should note that the loss of m^5^U cannot be quantified using this method, as reverse-transcription-based methods are blind to tRNA modifications that do not affect the Watson–Crick base pairing^86^. Altogether, we found that Nano-tRNAseq can reveal RNA modification interdependencies at distinct tRNA sites within the same isoacceptor and quantify site-specific modification changes across tRNA isoacceptors in a high-throughput manner, with the concurrent benefit of measuring tRNA abundances.

### Nano-tRNAseq reveals 3′ deadenylation upon oxidative stress

Previous works have shown that both tRNA abundances^57^ and modifications can be re-programmed under stress conditions, such as elevated temperature^122^ and oxidative stress^48^. Studies that quantified changes in tRNA abundances upon stress used NGS-based methods, which do not capture RNA modification information (with some exceptions in which the RNA modifications affect the reverse transcription signature). On the other hand, studies that quantified tRNA modification changes employed LC–MS/MS-based methods, which do not provide information about which tRNA isoacceptor the modification detected originates from.

**Fig. 5:**
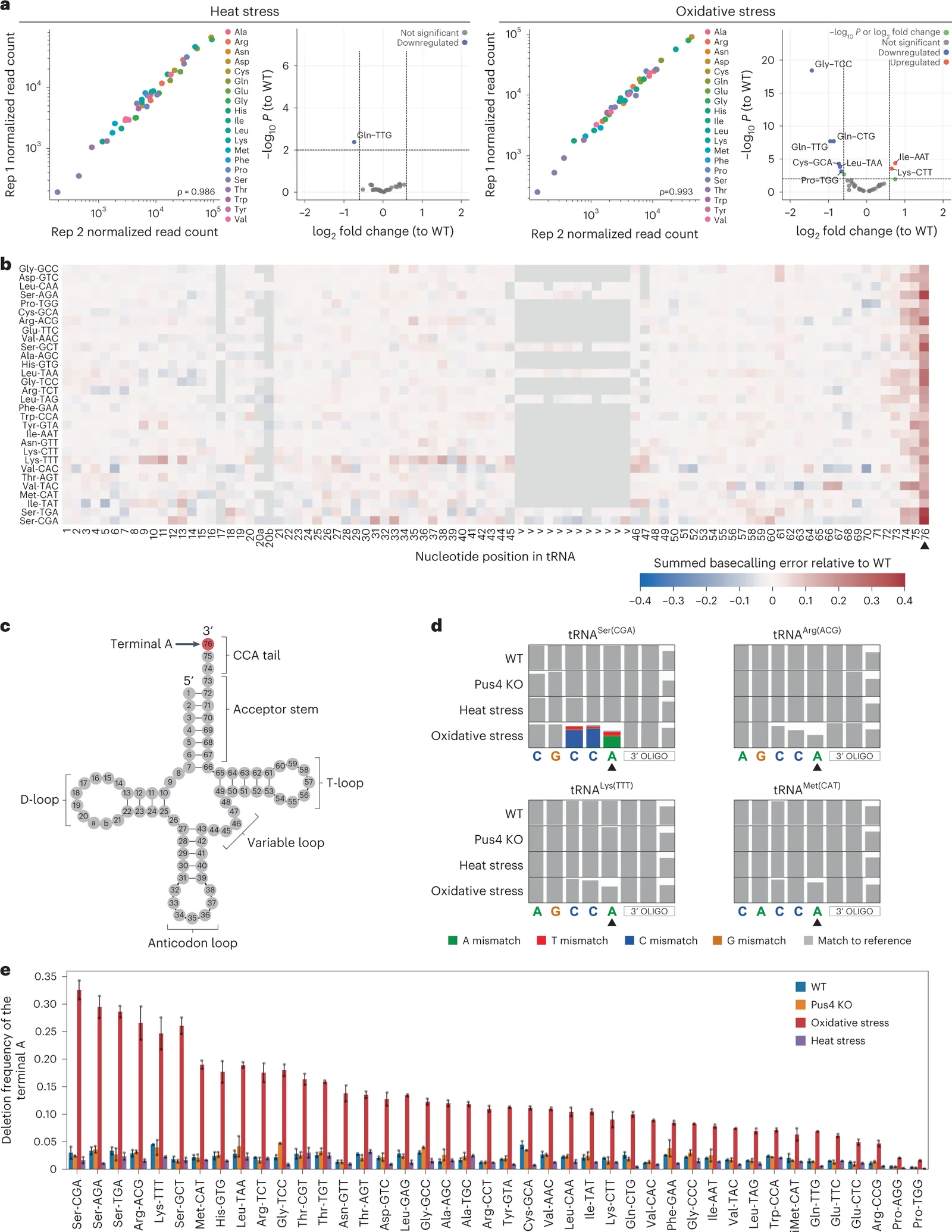
Characterization of tRNA abundance and modification dynamics upon exposure to stress reveals that the CCA tail is deadenylated in oxidative stress. **a**, Scatter plots of tRNA abundances of *S. cerevisiae* heat stress (45 °C for 1 hour) and oxidative stress (2 mM H_2_0_2_ for 1 hour) across biological replicates. Each point represents a tRNA alloacceptor and is colored based on alloacceptor type. The correlation strength is indicated by Spearman’s correlation coefficient (ρ). See also Supplementary Table 21. Volcano plots depicting differentially expressed tRNAs (relative to the untreated condition) are also shown for each stress type. See also Supplementary Tables 22 and 23. Differentially expressed tRNAs were defined as having an adjusted log_10_ *P* < 0.01 and an absolute log_2_ fold change greater than 0.6. **b**, Heat map of summed basecalling error of oxidative stress relative to WT, for each nucleotide (*x* axis) and for each tRNA (*y* axis, ordered from most to least abundant in descending order). See also Supplementary Table 17. The positions of specific RNA modifications in each tRNA are listed in Supplementary Table 18. Nucleotides with a lower summed basecalling error frequency relative to WT are in blue tones, and those with a higher summed basecalling error frequency are in red tones, as seen with the terminal A at position 76 (black arrowhead). Heat maps corresponding to other biological replicates can be found in Supplementary Fig. 2b. **c**, Schematic of a generic *S. cerevisiae* cytoplasmic tRNA in its usual secondary structure with the terminal A nucleotide of the CCA tail highlighted in red. **d**, Zoomed snapshots of IGV tracks featuring the terminal A (black arrowhead). Positions with a mismatch frequency greater than 0.2 are colored, whereas those showing mismatch frequencies lower than 0.2 are shown in gray. **e**, Bar plot of the deletion frequency of the terminal A base for each *S. cerevisiae* tRNA isoacceptor under oxidative stress (red), Pus4 KO (orange) or heat stress (purple) or in WT conditions (blue) (Supplementary Table 24). Error bars represent mean ± s.d. for *n* = 2 biological replicates per condition.

To examine how stress exposures affect tRNA abundances and modification profiles and in which tRNA isoacceptors, we sequenced tRNAs from *S. cerevisiae* cells exposed to either heat or oxidative stress using Nano-tRNAseq. We found that stress exposures caused only mild effects in terms of tRNA abundances, compared to WT (Fig. 5a and Supplementary Tables 21–23), with significant changes in the abundance of one tRNA isoacceptor (tRNA^Gln(UUG)^) upon heat stress and seven tRNA isoacceptors upon oxidative stress (corresponding to 12% of tRNA isoacceptors mapped). To our surprise, we found only very modest differences in tRNA modification profiles upon stress exposures (Fig. 5b, Supplementary Fig. 2a,b and Supplementary Table 17), in contrast to previous reports^48^. To confirm our findings, we then performed LC–MS/MS on the same samples used for Nano-tRNAseq, which corroborated our observations that RNA modifications are not significantly dysregulated upon either of the two stress exposures tested (Fig. 4g, Supplementary Tables 19 and 20 and Methods).

On the other hand, upon oxidative stress, but not heat stress, we observed a substantial increase in basecalling error frequency of the last nucleotide, position 76 (Fig. 5b and Supplementary Fig. 2b), which corresponds to the terminal A of the CCA overhang (Fig. 5c). Examination of IGV^123–125^ tracks showed that the terminal A had reduced coverage relative to its neighboring bases (Fig. 5d), which is indicative of a deletion (see Supplementary Figs. 3–23 for the IGV tracks of all tRNA isoacceptors across *S. cerevisiae* runs). We then calculated the deletion frequency of the terminal A for each tRNA isoacceptor and found that the deletion frequency in tRNAs subjected to oxidative stress was significantly higher compared to WT, Pus4 KO and heat stress (Fig. 5e and Supplementary Table 24), in agreement with a previous study that reported rapid loss of terminal A of the 3′ CCA tail during oxidative stress^126^.

## Discussion

For many years, tRNAs and their modifications have been primarily viewed as static contributors to gene expression and tRNA structure^127–130^. However, multiple studies have shown that tRNA abundances and modification profiles are, in fact, dynamic and can differ in distinct cellular environments and diseases^52,59,131,132^. Measuring both tRNA abundances and their modifications with single-transcript resolution has not been feasible due to a lack of available methods that can simultaneously capture both features. This has been a substantial limitation in moving forward with studying the biological function and dynamics of tRNA populations and their modifications and, consequently, their involvement in human diseases, among other aspects.

Our method, Nano-tRNAseq, enables the accurate and direct measurement of native tRNA molecule abundance and their modification status using nanopore DRS (Fig. 1b–d). During the library preparation protocol, the 5′ and 3′ ends of mature tRNAs are extended with RNA adapters, improving basecalling and mappability of the tRNA molecules. Notably, we found that double ligation of RNA adapters alone is insufficient to recapitulate known tRNA abundances and that the default MinKNOW configuration leads to biases in estimated tRNA abundances by preferentially capturing longer tRNA molecules. To overcome this limitation, we demonstrate that our customized MinKNOW configurations capture tRNA reads more efficiently, regardless of the tRNA length, and abrogate length-dependent biases (Fig. 3d–g). Moreover, by using this configuration, we demonstrate that the sequencing yield of tRNA runs increases up to 12-fold (Fig. 3c).

Recent works have also shown that ONT DRS can be used to quantify the expression of tRNAs, employing a double ligation of RNA:DNA adapters similar to the one described here^92^. However, we found that this approach alone is insufficient to recapitulate the abundance of tRNAs accurately (Fig. 3f) and leads to significantly lower sequencing yield (17× fewer sequenced reads and 15× fewer mapped reads compared to Nano-tRNAseq). Moreover, we eliminate the need for gel-mediated tRNA selection, which is not only cumbersome but also known to contribute to tRNA fragmentation^133^ and cause significant loss of material. In addition, the previously reported method is incompatible with the linearization of the tRNA molecule^92^; by linearizing tRNAs with our optimized reverse transcription protocol, we further increased sequencing yield by 50% (Extended Data Fig. 5d). Moreover, we demonstrate that Nano-tRNAseq can detect tRNA modifications (Fig. 4c–e and Extended Data Fig. 9) and quantify changes in their stoichiometry (Fig. 4d,e and Extended Data Fig. 8). On the other hand, compared to NGS-based approaches, Nano-tRNAseq directly sequences the native RNA molecule, thus circumventing the need to remove modifications that perturb reverse transcription^74,75^. Furthermore, it does not require PCR amplification, which is known to introduce unwanted variation in the sequencing results.

A notable feature that sets tRNAs apart from other RNA biotypes is the abundance and diversity of the modified bases in their structures^4,134,135^. Previous works have shown that the addition of a certain modification often depends on a pre-existing modification at another site^118–120,136,137^. Traditional methods for detecting the sequential addition of tRNA modifications, such as two-dimensional thin-layer chromatography (2D-TLC)^138^ and primer extension^139^, have contributed a wealth of knowledge to this area but are restricted by modification type and do not provide sequence or tRNA isoacceptor context. Similarly, high-performance liquid chromatography (HPLC)-based methods cannot provide sequence context, and, although HPLC–MS may be able to deduce sequence context through enzymolysis^67,140^, it is a targeted approach. Newer methods, namely NAIL-MS^141^ and NMR^120^, can dissect RNA modification circuits but are labor intensive and, in the latter case, are limited to studying specific tRNAs in isolation. In contrast, Nano-tRNAseq enables quantification of RNA modifications across the entire length of the transcript, in all tRNA isoacceptors, in a high-throughput and cost-effective manner, with the combined benefit of measuring tRNA abundances. To demonstrate this, we sequenced WT and Pus4-deficient *S. cerevisiae* and confirmed that Nano-tRNAseq could recapitulate the known relationship between loss of Ψ55, which Pus4 catalyzes, and the subsequent loss of m^1^A58 and m^5^U54 (Fig. 4e,f), in agreement with previous reports^120^. With the generation of yeast KO strains of every tRNA-modifying enzyme nearly complete (available from the Yeast Knockout Collection as part of the Saccharomyces Genome Deletion Project^142,143^), Nano-tRNAseq presents an excellent opportunity to describe tRNA modification circuits in a holistic manner, providing invaluable insights into how these processes are regulated and impact health and disease.

Previous studies have reported that some tRNA modifications are significantly altered under stress conditions^48^, likely conferring adaptation to environmental exposures^49,51^. Changes in tRNA modification levels may be attributed to the induction of new RNA modification enzymes, upregulated or attenuated expression of existing RNA modification enzymes or selective degradation of tRNAs. However, we did not observe significant changes in *S. cerevisiae* tRNA modification levels under oxidative stress or heat stress, neither using Nano-tRNAseq (Supplementary Fig. 2) nor using LC–MS/MS (Fig. 4g). The disparity in the results of our study compared to previous studies could likely be attributed to the difference in sample preparation; in Chan et al.^48^, LC–MS/MS was performed on ‘tRNA-containing small RNA species’, specifically small RNAs of 100 nt and fewer, and not just tRNAs. Therefore, the RNA fraction analyzed by Chan et al. could, in principle, contain tRNA-derived fragments (tRFs)^144^, fragments from other RNA biotypes (for example, mRNAs and rRNAs) as well as other small RNA species, such as miRNAs, snoRNAs and snRNAs, which also harbor RNA modifications, potentially contributing in the estimation of tRNA modification levels. By contrast, Nano-tRNAseq captures mature full-length tRNAs, and our LC–MS/MS experiments were conducted on gel-purified samples (70–110 nt; Supplementary Fig. 24c,d), which correspond to full-length tRNAs. Therefore, the differences in the results obtained between our study and previous works^48^ might be explained by differences in the input RNA pools (that is, mature tRNAs versus <100-nt RNAs that include tRNAs) that were used for sequencing and/or LC–MS/MS experiments.

All mature tRNAs contain a single-stranded CCA sequence at the 3′ terminus, which is generated and maintained by the CCA-adding enzyme ATP(CTP):tRNA nucleotidyltransferase, and is necessary for tRNA aminoacylation. Strikingly, we observed that the terminal A of the tRNA CCA tail was deadenylated under oxidative stress but not heat stress (Fig. 5b–e). Indeed, it has been previously shown that, under oxidative stress induced by sodium arsenite, the terminal A of the 3′ CCA sequence can be removed by endonuclease angiogenin^126^. Consistent with published results, although in this study oxidative stress is induced by H_2_O_2_, all tRNA isoacceptors exhibit 3′ CCA deadenylation. Regulation at the translation level through deadenylation of tRNA ends, thereby blocking their use in translation, could provide the plasticity for immediate changes in cellular activities and protein levels. Additionally, after removal of the stressor, the terminal A deadenylation is reversible and quickly repairable by the CCA-adding enzyme, thus making the tRNAs chargeable again, representing a rapid mechanism of suppressing and reactivating translation at a low metabolic cost. Using Nano-tRNAseq, we demonstrated this fast and dynamic translation repression by quantifying the terminal A deadenylation with tRNA isoacceptor resolution.

Although this study primarily uses tRNAs from *S. cerevisiae*, the natural next step would be to apply Nano-tRNAseq to a broader range of organisms and cell types. The modification profiles of lower eukaryotic species, such as *S. cerevisiae*, are mostly complete, while the modification profiles of only 18 out of 200 human cytosolic tRNAs are characterized in detail^134^. In this regard, Nano-tRNAseq can provide a means to catalog the RNA modification profiles for the tRNAs that lack this information. On the other hand, several studies have shown that tRNA dysregulation is associated with cancer progression and malignancy^16–18^, and that specific tRNAs are significantly upregulated as they gain metastatic activity^52,132,145^. However, tRNA abundances and modifications are currently not being used as screening, diagnostic or prognostic markers for cancer detection or progression, as the lack of cost-effective and reliable methodologies to detect and quantify tRNAs accurately has hindered their potential use as biomarkers. Nano-tRNAseq might offer an optimal solution to extract the maximal information from these molecules with minimal library preparation steps and use them as biomarkers for cancer screening and monitoring.

We should note that estimations of tRNA abundances obtained with Nano-tRNAseq will be limited to those tRNAs included in the reference FASTA set used in the mapping step. In this work, we chose to build a non-redundant set of *S. cerevisiae* tRNAs (Methods) that differed in at least 5% of its sequence (~2 nt), to avoid multi-mapping artifacts that would otherwise lead to biases in the tRNA abundance estimates^146^. Such reduction or clustering is commonly used in NGS-based studies^77,81,146^, as the relaxed mapping parameters used—to allow for mismatches caused by tRNA modifications—would otherwise lead to multi-mapping reads and, consequently, inaccurate tRNA abundance estimates. That being said, we should note that the tRNA reference used in this work contains at least one representative tRNA gene per tRNA isoacceptor, thus ensuring that Nano-tRNAseq can be used to investigate and identify modulations in tRNA isoacceptor abundances.

Collectively, Nano-tRNAseq is a sensitive and accurate method for the quantification of tRNA abundance and modification profiles with single-transcript resolution. The robust and straightforward library preparation workflow can be completed within a day and sequencing within a second day. We anticipate that our method will help shed new light on the dynamics of tRNA biology and may be used in the near future for diagnostics and prognostics of human disease.

## Methods

### Preparation of IVT transcribed tRNAs

A total of nine unmodified IVT tRNAs with diverse lengths and sequences (Supplementary Table 1) were prepared as previously described^147^. In brief, each tRNA was assembled using six DNA oligonucleotides that were first annealed and then ligated between HindIII and BamHI restriction sites of the plasmid pUC19. BstNI-linearized plasmids were used to perform the IVT with T7 RNA polymerase, according to standard protocols^148^. Transcripts were separated by 8 M urea/10% polyacrylamide gel electrophoresis. The tRNA was identified by UV shadowing, electroeluted and ethanol precipitated, and the tRNA pellet was resuspended in RNAse-free water. The integrity of the IVT tRNA products was confirmed (Supplementary Fig. 25) by running 200 ng of each sample on a 7 M urea/15% polyacrylamide gel (Life Technologies, EC6885BOX) in 1× TBE Buffer, using the Low Range ssRNA as a ladder (New England Biolabs (NEB), N0364S). Then, 2× RNA Loading Dye (Thermo Fisher Scientific, R0641) was added to each sample and ladder to a final volume of 1×, and the samples and ladder were heated at 95 °C for 3 minutes and cooled on ice before running. The gel was incubated in 1× TBE Buffer with 1× SYBR Gold Nucleic Acid Dye for 10–15 minutes with gentle agitation and visualized using a Bio-Rad Molecular Imager FX (ex: 495 nm, em: 537 nm).

### Removal of 5′ triphosphate of IVT tRNAs

The 5′ triphosphate was converted to 5′ monophosphate by incubating 1 µl of RppH enzyme (NEB, M0356S) per 100 ng of input IVT tRNAs, with 1× ThermoPol Buffer (NEB, B9004S), in a total reaction volume of 30 µl at 37 °C for 2 hours. The reaction was inactivated by adding 0.6 µl of 500 mM EDTA and incubating at 65 °C for 5 minutes, followed by cleanup using a Zymo RNA Clean and Concentrator-5 kit (Zymo Research, R1016), following the manufacturer’s instructions to retain RNAs ≥17 nt.

### Yeast strains and culturing

*S. cerevisiae* parental strain (BY4741), Pus1 KO strain (BY4741 MATa pus1::KAN), Pus4 KO strain (BY4741 MATa pus4::KAN) and Pus7 KO strain (BY4741 MATa pus7::KAN) were obtained from the Yeast Knockout Collection (Dharmacon) and grown under standard conditions overnight in 4 ml of YPD medium (1% yeast extract, 2% Bacto Peptone and 2% dextrose) at 30 °C. The next day, cultures were diluted to 0.0001 OD_600_ in 200 ml of YPD and grown overnight at 30 °C with shaking (250 r.p.m.). When cultures reached the mid-exponential growth phase (between OD_600_ 0.5), the WT culture was divided into 3 × 50 ml subcultures, which were then incubated for 1 hour at 30 °C (control), 45 °C (heat stress) or in 2 mM H_2_0_2_ (oxidative stress). The Pus4 culture was divided into 1 × 50 ml culture and incubated at 30 °C. After incubation, cultures were quickly transferred into a pre-chilled 50-ml Falcon tube and centrifuged at 3,000*g* for 5 minutes at 4 °C, followed by two washes with water, and then pellets were snap-frozen at −80 °C. Biological replicates were performed on consecutive days.

### RNA extraction from yeast cultures

Snap-frozen yeast pellets were resuspended in 660 µl of TRIzol Reagent (Thermo Fisher Scientific, 15596018) with 340 µl of acid-washed and autoclaved 425–600-µm glass beads (Sigma-Aldrich, G8772). The cells were disrupted by vortexing on top speed for seven cycles of 15 seconds and chilling the samples on ice for 30 seconds between cycles. The samples were then incubated at room temperature for 5 minutes, and 200 µl of chloroform was added. After briefly vortexing the suspension, the samples were incubated for 5 minutes at room temperature and centrifuged at 14,000*g* for 15 minutes at 4 °C. The upper aqueous phase was transferred to a new tube. To precipitate RNA, 1× volume of molecular-grade isopropanol and 1 µl of GlycoBlue co-precipitant (Thermo Fisher Scientific, AM9515) were added and mixed by inverting and incubated for 10 minutes at room temperature. The samples were centrifuged at 14,000*g* for 15 minutes at 4 °C, and the pellet was then washed with ice-cold 70% ethanol. The pellet was resuspended in nuclease-free water after air drying for 5 minutes on the benchtop, and the RNA purity was measured using a NanoDrop 1000 spectrophotometer. The samples were treated with Turbo DNase (Thermo Fisher Scientific, AM2238) and subsequently cleaned up using a Zymo RNA Clean and Concentrator-5 kit (Zymo Research, R1016) following the manufacturers’ instructions to retain RNAs ≤200 nt. In brief, 1× volume of RNA Binding Buffer was combined with 1× volume of 100% ethanol. Then, 2× volume of the RNA Binding Buffer and ethanol solution was added to the reaction, transferred to a Zymo-IC column and spun at ≥12,000*g* at room temperature for 1 minute. Next, 1× volume of 100% ethanol was added to the flow-through, which contains the 17–200-nt fraction, and this was transferred to a new Zymo-IC column and spun at ≥12,000*g* at room temperature for 1 minute. Then, 400 µl of RNA Prep Buffer was added to the column and spun at ≥12,000*g* at room temperature for 1 minutes, and then 800 µl of RNA Wash Buffer was added, and the column was spun at >12,000*g* at room temperature for 2 minutes, transferred to a fresh collection tube and spun for 1 minute. The RNA was eluted in nuclease-free water. RNA concentration was determined using Qubit Fluorometric Quantitation; RNA purity was measured with a NanoDrop 1000 spectrophotometer; and the RNA electropherogram was obtained using Agilent 4200 TapeStation RNA HS ScreenTape Assay (Supplementary Fig. 24a).

### tRNA deacylation

Commercial *S. cerevisiae* tRNA^Phe^ (Sigma-Aldrich, R4018), commercial *S. cerevisiae* total tRNA (Sigma-Aldrich, AM7119) and tRNAs purified from *S. cerevisiae* BY4741 WT and Pus4 KO cultures were resuspended in 10 µl of nuclease-free water and deacylated in 95 µl of 100 mM Tris-HCl (pH 9.0) at 37 °C for 30 minutes. Deacylated tRNAs were recovered using Zymo RNA Clean and Concentrator-5 kit (Zymo Research, R1016), following the manufacturer’s instructions to retain RNAs ≥17 nt but increasing the ethanol concentration to 1.3× after the RNA Prep Buffer step. The tRNA profiles were confirmed using Agilent 4200 TapeStation RNA HS ScreenTape Assay (Supplementary Fig. 24b).

### Nanopore tRNA sequencing library preparation (Nano-tRNAseq)

tRNA libraries were prepared using the SQK-RNA002 kit (ONT) with some protocol alterations as described here. All oligonucleotides used in this study were obtained from Integrated DNA Technologies (IDT) (Supplementary Table 25). The 5′ RNA splint adapter (/5/rCrCrUrArArGrArGrCrArArGrArArGrArArGrCrCrUrGrGrN) was designed to be complementary to the 3′ NCCA overhang of mature tRNAs, and the 3′ splint RNA:DNA adapter (/5Phos/rGrGrCrUrUrCrUrUrCrUrUrGrCrUrCrUrUrArGrGrArArArArArArArArArAAAA) was designed to be complementary to the rest of the 5′ RNA splint adapter, with a short poly(A) segment for the RTA adapter to anneal to (Fig. 1b and Extended Data Fig. 1). The 5′ and 3′ splint adapters were prepared at a 1:1 molar ratio in a solution of 10 mM Tris-HCl (pH 7.5), 50 mM NaCl and 1 µl of RNasin Ribonuclease Inhibitor (Promega, N251A), with a final concentration of 50 ng µl^−1^ and heated to 75 °C for 15 seconds and cooled to 25 °C at a rate of 0.1 °C s^−1^ to hybridize the adapters. DNA oligos with the same sequence as ONT RTA adapters were ordered from IDT and annealed in the same manner as the 5′ and 3′ splint adapters. Deacylated tRNAs were ligated to the pre-annealed 5′ and 3′ splint adapters at a molar ratio of 1.2:1 (assuming an average tRNA length of 90 nt). The ligation was carried out at room temperature for 2 hours in a total reaction volume of 50 µl with 20% PEG 8000 (NEB, B10048), 1× T4 RNA Ligase 2 Buffer (NEB, B0239S), 4 µl of 6 mg ml^−1^ recombinant *E. coli* T4 RNA 2 Ligase (made in-house; see below) and 1 µl of RNasin Ribonuclease Inhibitor (Promega, N251A). A 2× volume of room-temperature-equilibrated AMPure RNAClean XP beads (Beckman Coulter, A63987) was then added to the reaction, pipetting gently up and down, and incubated for 15 minutes at room temperature on a Hula Mixer. The beads were washed with freshly prepared 70% ethanol and left to air dry. The samples were eluted by resuspending the beads in nuclease-free water and incubating them for 10 minutes at room temperature on a Hula Mixer. The RNA concentration was determined using RNA HS Qubit Fluorometric Quantification. Then, 200 ng of 5′ and 3′ ligated tRNAs were ligated to the pre-annealed RTA adapters at a molar ratio of 1:2 (roughly 4.3 pmol tRNAs to 8.6 pmol of RTA adapter). The ligation was carried out at room temperature for 30 minutes in a total reaction volume of 15 µl with 1× Quick Ligation Reaction buffer (NEB, B6058S), 1.5 μl of T4 DNA Ligase (NEB, M0202M, 2,000,000 units per milliliter) and 0.5 µl of RNasin Ribonuclease Inhibitor (Promega, N251A). After ligation, a reverse transcription master mix of 13 µl of nuclease-free water, 2 µl of 10 mM dNTPs (NEB, N0447S), 8 µl of 5× Maxima H Minus Reverse Transcriptase Buffer and 2 µl of Maxima H Minus Reverse Transcriptase (Life Technologies, EP0751) were added directly to the reaction, mixed well by pipetting and incubated at 60 °C for 1 hour, 85 °C for 5 minutes and then brought to 4 °C. The linearized tRNAs were cleaned up using 2× AMPure RNAClean XP beads as described for the ligation reaction. Finally, the ONT RMX sequencing adapters were ligated at room temperature for 30 minutes in a total reaction volume of 40 µl with 1× Quick Ligation Reaction buffer (NEB, B6058S), 3 μl of T4 DNA Ligase (NEB, M0202M, 2,000,000 units per milliliter) and 6 µl of RMX adapters. A 2× volume of AMPure RNAClean XP beads was then added and mixed into the reaction by pipetting gently up and down and incubated for 10 minutes at room temperature on a Hula Mixer. The sample was washed twice with 150 μl of WSB (Wash Buffer), in which the pellet was resuspended by flicking the tube. The sample was eluted in 20 μl of ELB (Elution Buffer) and incubated for 10 minutes at room temperature on a Hula Mixer. The final library was prepared by adding 17.5 μl of nuclease-free water and 37.5 μl of vortexed RRB and kept on ice until loading. The MinION flow cell (FLO-MIN-106) was quality controlled, primed and loaded as per the standard ONT SQK-RNA002 protocol.

### Alternative nanopore tRNA sequencing strategies tested

Below we describe the initial strategies tested to build nanopore tRNA DRS libraries (Strategy A and Strategy B), which are not recommended. However, details to build them are included below to ensure that all results included in this work can be reproduced if desired.

#### Strategy A

tRNA DRS libraries were prepared using the SQK-RNA002 kit (ONT) with some protocol alterations as described here for the following library preparation protocol strategies (Extended Data Fig. 1). Deacylated tRNAs were polyadenylated using *E. coli* poly(A) polymerase (NEB, M0276S) at 37 °C for 30 minutes following the manufacturer’s instructions. The 5′ RNA splint adapter, as used in Nano-tRNAseq and all library preparation strategies described, was ligated to poly(A)-tailed tRNAs at a molar ratio of 2:1. The reaction was carried out overnight at 4 °C with 20% PEG 8000, 1× T4 RNA Ligase 2 Buffer, 4 µl of 6 mg ml^−1^ recombinant *E. coli* T4 RNA 2 Ligase and 1 µl of RNaseOUT (Invitrogen, 18080051), in a total reaction volume of 50 µl. A 1.8× volume of AMPure RNAClean XP beads was then added and mixed into the reaction by pipetting gently up and down and incubated for 15 minutes at room temperature on a Hula Mixer. The beads were washed with freshly prepared 70% ethanol and left to air dry. To elute, the beads were resuspended in nuclease-free water and incubated for 10 minutes at room temperature on a Hula Mixer. RNA concentration was determined using Qubit Fluorometric Quantification. The ligation of RTA and RMX adapters, final library preparation steps and flowcell quality control and loading are as described in Nano-tRNAseq.

#### Strategy B

tRNA DRS libraries were prepared using the SQK-RNA002 kit (ONT) with some protocol alterations as described here for the following library preparation protocol strategies (Extended Data Fig. 1). The 5′ splint RNA adapter (/5/rCrCrUrArArGrArGrCrArArGrArArGrArArGrCrCrU rGrGrN) and ONT RTA adapter oligo A were annealed in a molar ratio of 1:1 as described above. The annealed 5′ splint RNA adapter and 3′ splint DNA adapter were ligated to 5′ monophosphate, deacylated tRNAs and cleaned up using the same protocol as in Strategy A. The ligation of RMX adapters, final library preparation steps and flowcell quality control and loading are as described in Nano-tRNAseq.

### Recombinant protein expression of *E. coli* T4 RNA Ligase 2

The codon-optimized sequence of *E. coli* T4 RNA Ligase 2 (T4RNL2) ORF DNA was ordered from IDT and cloned into the expression plasmid pETM14 in frame, with a coding sequence of a hexa-histidine tag followed by a 3C PreScission cleavage recognition sequence. The protein expression and purification were performed in the Protein Technologies Unit at the Center for Genomic Regulation (CRG), following previously described procedures^101^. For long-term storage at −80 °C, glycerol was added to a final concentration of 10%. For assays, 6 mg ml^−1^ recombinant *E. coli* T4 RNA 2 Ligase was kept in 10 mM Tris-HCl, 50 mM KCl, 35 mM (NH_4_)_2_SO_4_, 0.1 mM DTT, 0.1 mM EDTA and 50% glycerol at −20 °C.

### Gel purification of tRNAs and LC–MS/MS

Gel-purified tRNAs were only used for LC–MS/MS. First, 5 µg of the 17–200-nt fraction of each sample, and commercial *S. cerevisiae* tRNA^Phe^ and total tRNA, which served as size markers, were prepared in 2× RNA loading dye (NEB, B0363A) and heat denatured at 94 °C for 5 minutes. Running samples were loaded into 15% 7 M TBE-urea gels (Life Technologies, EC6885BOX) with a lane left free between each sample to avoid cross-contamination and run in 1× TBE at 100 V until the bromophenol blue marker was at three-quarters of the way down the gel. The gel was post-stained in the dark in 1× TBE with 1× SYBR Gold (Invitrogen, S11494) for 5 minutes. Gels were transferred to copier transparency film (Niceday, 607510), and, using UV underlighting, the gel region corresponding to tRNAs (Supplementary Fig. 24c) was excised using a sterile scalpel and transferred into a Zymo-Spin IV Column from the ZR small-RNA PAGE Recovery Kit (Zymo Research, R1070). tRNAs were extracted from the gel as per manufacturer instructions, and the extracted tRNA profiles were confirmed using Agilent 4200 TapeStation RNA HS ScreenTape Assay (Supplementary Fig. 24d). Then, 500 ng of gel-purified tRNAs were digested at 37 °C for 1 hour using Nucleoside Digestion Mix (NEB, M0649), following manufacturer instructions. The nucleoside digestion solution was then desalted using HyperSep SpinTip Column (Thermo Fisher Scientific, 60109-404). First, the column was washed with 40 μl of 60% acetonitrile by centrifuging at 100*g* for 10 minutes and then washed with 40 μl of 0.1% formic acid by centrifuging at 100*g* for 5 minutes. The digested sample was combined with 30 μl of formic acid, added to the column and collected in a fresh collection tube by centrifuging at 100*g* for 10 minutes. The flow-through was re-applied to the column and centrifuged at 100*g* for 10 minutes. Now bound to the column, the sample was washed with 40 μl of 0.1% formic acid by centrifuging at 100*g* for 5 minutes. Next, 40 μl of 60% acetonitrile was added to the column, and the sample was eluted by centrifuging at 100*g* for 5 minutes. The CRG/UPF Proteomics Facility conducted LC–MS/MS of *S. cerevisiae* tRNA modifications. In brief, 125 ng of each digested and desalted sample was analyzed by LC–MS/MS using a 40-minute gradient on an Orbitrap XL. As a quality control, ribonucleoside standards were run between samples to prevent carryover and to assess the instrument performance (see Supplementary Table 19 for raw data and Supplementary Table 20 for normalized data). Heat stress replicate 2 had an altered chromatographic profile with significantly less Ψ than all other samples and was, therefore, discarded from the analysis.

### tRNA reverse transcription optimization

IVT tRNAs and commercial *S. cerevisiae* tRNA^Phe^ were poly(A) tailed (as described in Strategy A) and used for reverse transcription tests. For the SuperScript II, 100 ng of poly(A)-tailed RNA, 1 µl of 100 µM 3′ reverse transcription test adapter (see Supplementary Table 25 for oligonucleotides) and 1 µl of 10 mM dNTP (Promega, M750B) were combined in a total reaction volume of 12 µl, incubated at 65 °C for 5 minutes and then chilled on ice. Then, 4 µl of either 5× first-strand (FS) buffer (Thermo Fisher Scientific, 18064014) or 5× FS buffer supplemented with 65 mM MnCl_2_, 1 µl of 0.1 M DTT, 1 µl of RNaseOUT and 1 µl of SuperScript II reverse transcriptase (Thermo Fisher Scientific, 18064014) were added, and the reaction was incubated at 42 °C for 1 hour and inactivated by heating at 70 °C for 15 minutes, followed by RNAse digestion. For SuperScript IV, 100 ng of poly(A)-tailed RNA, 1 µl of 100 µM 3′ reverse transcription test adapter and 1 µl of 10 mM dNTP were combined in a total reaction volume of 12 µl, incubated at 65 °C for 5 minutes and then chilled on ice. Then, 4 µl of 5× SuperScript IV reverse transcription buffer (Thermo Fisher Scientific, 18090010), 1 µl of 0.1 M DTT, 1 µl of RNaseOUT and 1 µl of SuperScript IV reverse transcriptase (Thermo Fisher Scientific, 18090010) were added, and the reaction was incubated at 55 °C or 60 °C for 1 hour and inactivated by heating at 85 °C for 5 minutes, followed by RNAse digestion. For TGIRT, 100 ng of poly(A)-tailed RNA, 1 µl of 100 µM 3′ reverse transcription test adapter, 4 µl of 5× TGIRT reverse transcription buffer, 1 µl of 0.1 M DTT, 1 µl of TGIRT-III (InGex, TGIRT50) and 1 µl of RNaseOUT were combined in a total reaction volume of 19 µl and incubated at room temperature for 30 minutes. Then, 1 µl of 10 mM dNTPs was added, and the reaction was incubated at 60 °C for 1 hour and inactivated by heating at 75 °C for 15 minutes, followed by RNAse digestion. For Maxima, 100 ng of poly(A)-tailed RNA, 1 µl of 100 µM 3′ reverse transcription test adapter and 1 µl of 10 mM dNTP were combined in a total reaction volume of 12 µl, incubated at 65 °C for 5 minutes and then chilled on ice. Then, 4 µl of 5× Maxima reverse transcription buffer, 1 µl of RNaseOUT and 1 µl of Maxima H Minus reverse transcriptase (Thermo Fisher Scientific, EP0751) were added, and the reaction was incubated at 55 °C or 60 °C for 1 hour and inactivated by heating at 85 °C for 5 minutes, followed by RNAse digestion. After reverse transcription, the RNA was digested by adding 1.5 µl of RNase Cocktail Enzyme Mix (Thermo Fisher Scientific, AM2286) to the reaction and incubating at 37 °C for 10 minutes. The reactions were cleaned up using 1.5× AMPure XP beads as described, and the tRNA cDNA and input poly(A) tRNA was run on TapeStation using the RNA HS assay.

### *S. cerevisiae* tRNA reference set

Reference sequences for mature *S. cerevisiae* tRNAs were retrieved from GtRNAdb2 (ref. ^149^). GtRNAdb2 reports 275 tRNA sequences annotated in the *S. cerevisiae* genome. Most tRNA isoacceptors (that is, with the same anticodon) have multiple copies; for example, Asp-GTC and Gly-GCC have 16 copies each, and most of these copies are identical—only 54 unique, mature tRNA sequences exist. From these, 12 sequences are highly similar to other tRNA genes, having 95–99% identity (Supplementary Table 26) with another tRNA gene; for example, Asp-GTC-1 and Asp-GTC-2 have an identity of 96.9%. To facilitate reliable alignment and accurate tRNA quantification, we kept the 42 sequences that were at least 5% divergent at nucleotide level (including ligated 5′ and 3′ oligos), which kept one reference tRNA gene per tRNA isoacceptor. The final reference file used in this work is available in the GitHub repository (https://github.com/novoalab/Nano-tRNAseq). Modifications for *S. cerevisiae* tRNAs were obtained from MODOMICS^22^, and the canonical position was manually curated using published literature (Supplementary Table 18)^3,25,117,150^.

### Basecalling and mapping tRNA reads

Reads were basecalled using Guppy basecaller version 3.6.1 in high-accuracy (hac) mode. All Us were converted to Ts before mapping. Basecalled reads were mapped using minimap2 version 2.17-r941 with recommended parameters (-ax map-on -k15) or sensitive parameters (-ax map-ont -k5) or BWA version 0.7.17-r1188. For BWA, two modes (MEM and SW) were tested, and several sets of parameters were invoked as follows (ordered from the most stringent to the least stringent settings): (1) bwa mem -W13 -k6 -xont2d; (2) bwa mem -W13 -k6 -xont2d -T20; (3) bwa mem -W13 -k6 -xont2d -T10; (4) bwa mem -W9 -k5 -xont2d -T10; and (5) bwa sw -z10 -a2 -b1 -q2 -r1 (Supplementary Table 3). Reads mapping to the reverse strand (antisense) were assigned as ‘wrong alignments’. We selected the best-performing algorithm and parameters (bwa mem -W13 -k6 -xont2d -T20) by comparing the number of uniquely aligned reads and the number of wrong alignments (Supplementary Table 4). We should note that the sequence of 5′ and 3′ RNA adapters were included in the respective references when mapping the tRNA reads. The effect of 5′ and 3′ RNA adapters length on the mappability was tested by shortening the respective adapter sequence from the alignment reference with a step of 5 nt (Supplementary Table 6). All reference files used in this work are available in the GitHub repository (https://github.com/novoalab/Nano-tRNAseq).

### Analysis of tRNA abundances

tRNA abundances were quantified using the get_counts.py script (available on GitHub: https://github.com/novoalab/Nano-tRNAseq). Unique (mapping quality above 0) primary alignments were considered. Differentially expressed tRNAs were inferred using DESeq2 (ref. ^151^). Volcano plots were generated using the EnhancedVolcano package^152^. Differentially expressed tRNAs were defined as those having adjusted *P* < 0.01 and absolute log_2_ expression fold change greater than 0.6.

### Analysis of differential tRNA modifications

Differential tRNA modifications were measured using differential basecalling errors (mismatch, insertion and deletion) for each tRNA nucleotide. The sum of basecalling errors was calculated by subtracting the frequency of the reference base from 1. The frequency of the reference base equals the number of reads with a basecalled equivalent to the reference base, divided by the depth of coverage for that position. Only uniquely aligned reads (primary alignment with mapping quality above 0) were considered. To ease the above calculations, we developed a script (get_sum_err.py), which reports the sum of basecalling errors and frequencies of A, C, G, T, deletions and insertions for every position of tRNA reference as well as plotting heat maps that order tRNA isoacceptors from highest to lowest expressed (plot_heatmap.py). The script is available at https://github.com/novoalab/Nano-tRNAseq. For heat maps, only tRNAs whose sequences were consistent between the tRNAdb2 and MODOMICS databases could be used, with the exception of His-GTG, whose sequence varied between tRNAdb2 and MODOMICS databases. The disparity was manually resolved by replacing the first base in the MODOMICS alignment with a gap. The difference in Ψ55 modification stoichiometry between WT and Pus4 KO was quantified using NanoRMS^98^, using a supervised *k*-nearest neighbor (KNN) classification algorithm, incorporating signal intensity and trace features. Ψ55 sites with coverage lower than 5 reads in either the WT or Pus4 KO condition were excluded from the NanoRMS analysis. The script is available at https://github.com/novoalab/nanoRMS.

### Adjusting MinKNOW parameters to capture small RNAs

Sequencing runs were conducted without live basecalling, and the bulk dump raw file was recorded for a subset (channels 1–50 for runID 4_NanotRNAseq_IVT + tRNAphe) or all 512 channels (all remaining runs). MinKNOW version 21.06.0 was used for sequencing and running the simulations with distinct MinKNOW parameter settings from raw data dumps. The sequencing simulations were performed with default and custom MinKNOW configurations. By default, MinKNOW defines adapter duration as up to 5 seconds and the strand (an actual read) as at least 2 seconds. Thus, the RNA molecule has to spend up to 7 seconds in the pore to be classified and reported as an actual read. The motor protein (RNA helicase) used in DRS experiments has an average speed of 70 nt per second; thus, 7 seconds corresponds to roughly 490 nt. Such a definition makes sense for long-molecule sequencing, as it filters out the adaptor-only reads. However, for short RNA sequencing, it would be reasonable to shorten both the adapter and strand definitions. We evaluated several configurations, shortening the duration of the adapter to 1 second and the strand to 1, 2, 3 or 4 seconds. Subsequently, the number of reported, basecalled, aligned and uniquely aligned reads generated by default and custom MinKNOW configurations were compared. We concluded that using the 1 second definition for the adapter and 2 seconds for the strand (Extended Data Fig. 4) resulted in the highest number of aligned and uniquely aligned reads (Supplementary Table 7). Therefore, those settings are used across this study unless stated otherwise. Alternative MinKNOW configuration files are deposited and described in detail in the GitHub repository: https://github.com/novoalab/Nano-tRNAseq.

### Comparisons with published datasets

Nano-tRNAseq *S. cerevisiae* tRNA expression estimations were compared to estimates reported by orthogonal Illumina-based tRNA sequencing methods ARM-seq^74^, Hydro-tRNAseq^80^ and mim-tRNAseq^77^. The published estimates were reported per tRNA isoacceptor–anticodon pair and included the same references as the ones used in this work, with the exception of Hydro-tRNAseq, which missed two references (Leu-GAG and iMet-CAT) and reported an additional five references (Leu-AAG, Leu-CAG, Ala-CGC, Pro-CGG and Arg-TCG). These references were excluded from pairwise comparisons with Hydro-tRNAseq. HydraPsiSeq data were obtained from the authors^121^, and reads were mapped to *S. cerevisiae* tRNAs as described above (without the Nano-tRNAseq adapters included in the reference) but using adjusted bwa mem parameters -W13 -k6 -L0 -T15 to capture HydraPsiSeq reads, which are shorter. The summed mismatch error for each nucleotide in each Pus4 KO tRNA isoacceptor was calculated relative to WT, as described above.

### Reporting Summary

Further information on research design is available in the Nature Portfolio Reporting Summary linked to this article.

## Online content

Any methods, additional references, Nature Portfolio reporting summaries, source data, extended data, supplementary information, acknowledgements, peer review information; details of author contributions and competing interests; and statements of data and code availability are available at https://doi.org/10.1038/s41587-023-01743-6.

## Supplementary information


Supplementary InformationSupplementary Figs. 1–25.
Reporting Summary
Supplementary TableSupplementary Tables 1–27.


## Data Availability

Basecalled FAST5 nanopore data have been deposited in the European National Archive (ENA) under accession PRJEB55684. From these data, both basecalled FAST5 and/or FASTQ files can be acquired^153^. FASTQ from HydraPsiSeq data^121^ has also been deposited in ENA under accession PRJEB55684 (ref. ^153^). A description of all the runs used in this work is included in Supplementary Table 7 and Supplementary Table 27. The list of tRNA modifications present in *S. cerevisiae* tRNAs was obtained from MODOMICS (https://iimcb.genesilico.pl/modomics/sequences/) and was retrieved on 21 September 2021. tRNA expression estimates from Illumina-based *S. cerevisiae* tRNA sequencing were obtained from following sources: mim-tRNAseq (Gene Expression Omnibus: GSE152621), tRNA-HydroSeq (Supplementary Material of the publication) and ARM-seq (Supplementary Table 2 of the publication).

## Code availability

The reference FASTA, alignment and modification BED files, custom MinKNOW configurations and all code used to analyze the runs are publicly available on GitHub (https://github.com/novoalab/Nano-tRNAseq)^154^. The NanoRMS script is available at https://github.com/novoalab/nanoRMS (ref. ^98^).
