# Supplementary material for: Quantitative analysis of tRNA abundance and modifications by nanopore RNA sequencing
Source: Nat Biotechnol. 2023 Apr 6;42(1):72–86. doi: 10.1038/s41587-023-01743-6 (PMC10791586; doi:10.1038/s41587-023-01743-6)

nature biotechnology

Article

https://doi.org/10.1038/s41587-023-01743-6

Quantitative analysis of tRNA abundance and modifications by nanopore RNA sequencing

In the format provided by the authors and unedited

Table of Contents

Supplementary Figure 1. Orthogonal validation using HydraPsiSeq shows that m1A58 levels are decreased upon Pus4-dependent loss of Ψ55.

Supplementary Figure 2. tRNA modification changes upon exposure to environmental stress are highly replicable.

Supplementary Figure 3. IGV snapshots of S. cerevisiae tRNAAla(AGC) and tRNAAla(TGC).

Supplementary Figure 4. IGV snapshots of S. cerevisiae tRNAArg(ACG) and tRNAArg(CCG).

Supplementary Figure 5. IGV snapshots of S. cerevisiae tRNAArg(CCT) and tRNAArg(TCT).

Supplementary Figure 6. IGV snapshots of S. cerevisiae tRNAAsn(GTT) and tRNAAsp(GTC).

Supplementary Figure 7. IGV snapshots of S. cerevisiae tRNACys(GCA) and tRNAGln(GTC).

Supplementary Figure 8. IGV snapshots of S. cerevisiae tRNAGln(TTG) and tRNAGlu(CTC).

Supplementary Figure 9. IGV snapshots of S. cerevisiae tRNAGlu(TTC) and tRNAGly(CCC).

Supplementary Figure 10. IGV snapshots of S. cerevisiae tRNAGly(GCC) and tRNAGly(TCC).

Supplementary Figure 11. IGV snapshots of S. cerevisiae tRNAHis(GTG) and tRNAIle(AAT).

Supplementary Figure 12. IGV snapshots of S. cerevisiae tRNAIle(TAT) and tRNAiMet(CAT).

Supplementary Figure 13. IGV snapshots of S. cerevisiae tRNALeu(CAA) and tRNALeu(GAG).

Supplementary Figure 14. IGV snapshots of S. cerevisiae tRNALeu(TAA) and tRNALeu(TAG).

Supplementary Figure 15. IGV snapshots of S. cerevisiae tRNALys(CTT) and tRNALys(TTT).

Supplementary Figure 16. IGV snapshots of S. cerevisiae tRNAMet(CAT) and tRNAPhe(GAA).

Supplementary Figure 17. IGV snapshots of S. cerevisiae tRNAPro(AGG) and tRNAPro(TGG).

Supplementary Figure 18. IGV snapshots of S. cerevisiae tRNASer(AGA) and tRNASer(CGA).

Supplementary Figure 19. IGV snapshots of S. cerevisiae tRNASer(GCT) and tRNASer(TGA).

Supplementary Figure 20. IGV snapshots of S. cerevisiae tRNAThr(AGT) and tRNAThr(CGT).

Supplementary Figure 21. IGV snapshots of S. cerevisiae tRNAThr(TGT) and tRNAThr(CCA).

Supplementary Figure 22. IGV snapshots of S. cerevisiae tRNATyr(GTA) and tRNAVal(AAC).

Supplementary Figure 23. IGV snapshots of S. cerevisiae tRNAVal(CAC) and tRNAVal(TAC).

Supplementary Figure 24. Quality and purity of yeast RNA samples used in this study.

Supplementary Figure 25. Purity of in vitro transcribed tRNAs used in this study.

Supplementary Figure 1. Orthogonal validation using HydraPsiSeq shows that m¹A58 levels are decreased upon Pus4-dependent loss of Ψ55. (A) General overview of the HydraPsiSeq protocol. RNA is treated by hydrazine and subjected to aniline cleavage. 3′-phosphates are removed by T4 PNK treatment, and adapters are ligated to 3′- and 5′-ends of RNA fragments. After sequencing and alignment to a reference sequence, 5′-ends of all fragments are counted to generate U cleavage profiles. U residues are sensitive to hydrazine and thus efficiently cleaved, while ψ residues are resistant and provide only background signals. **(B)** IGV snapshots of wild-type (WT) and Pus4 knockout (KO) *S. cerevisiae* tRNA. The Ψ55 position is indicated by a red arrowhead, and in the Pus4 KO condition, the coverage drops off +1 nucleotide from U55. The m¹A58 position is indicated by a green arrowhead and, in the WT condition, manifests as a mismatch. **(C)** Scatterplot of the summed error of m¹A sites of WT versus Pus4 KO S. cerevisiae tRNAs sequenced with HydraPsiSeq. Each point represents a tRNA alloacceptor and is colored based on alloacceptor type. **(D)** Heatmap of summed basecalling error of Pus4 KO relative to WT, for each nucleotide (x-axis), for each tRNA isoacceptor, from HydraPsiSeq (y-axis, ordered from most to least abundant in descending order). The positions of specific RNA modifications in each tRNA are listed in **Table S18**. Nucleotides with a higher mismatch frequency relative to WT are in red tones, and those with a lower mismatch frequency are in blue tones, as seen with m1A58 (green arrowhead), indicating that it is down-regulated upon loss of Pus4.

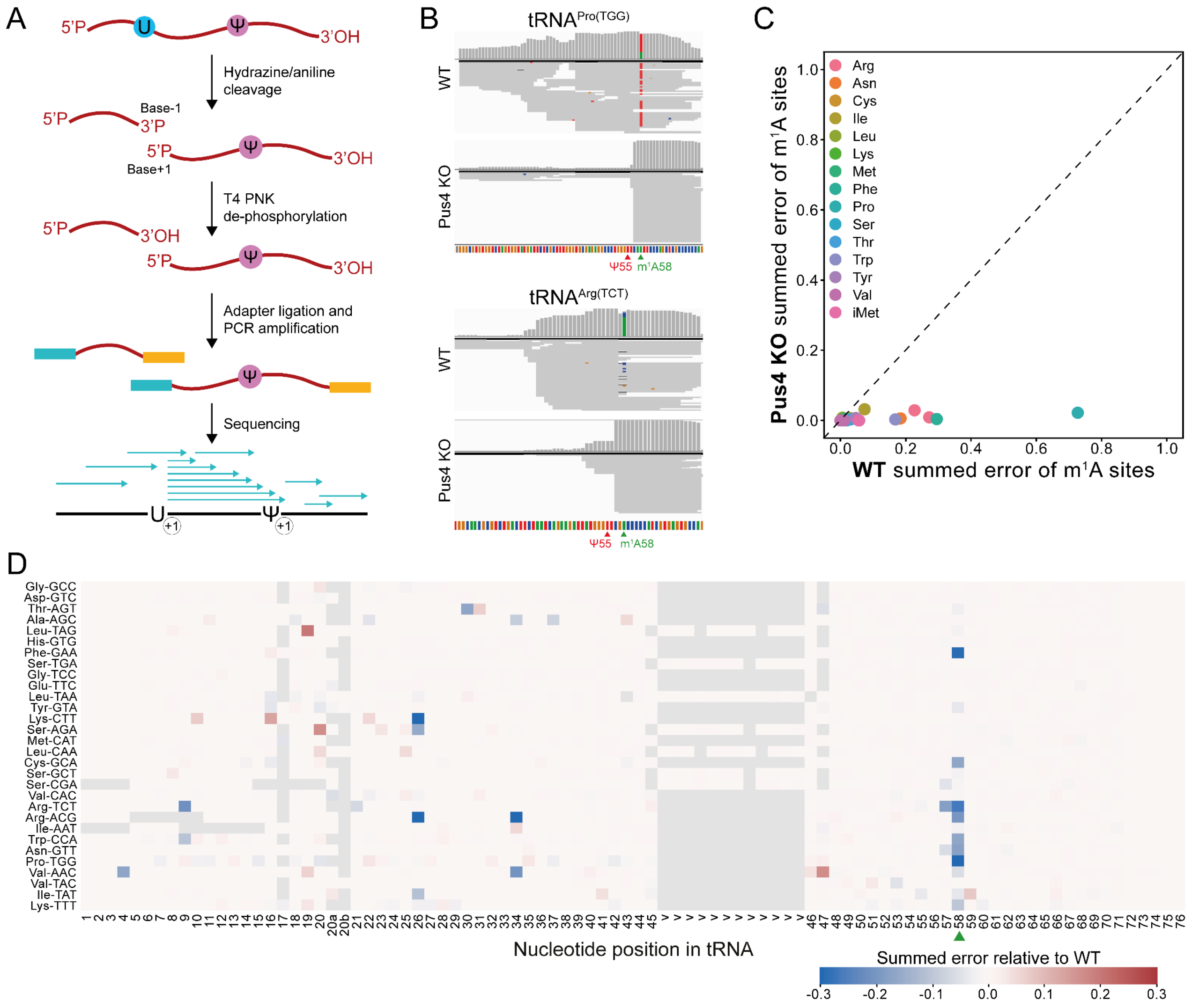

Supplementary Figure 2. tRNA modification changes upon exposure to environmental stress are highly replicable. Heatmap of summed basecalling error frequency of **(A)** heat stress biological replicate 1 and 2 and **(B)** oxidative stress biological replicate 1 (as in **Figure 5B**) and 2, relative to wild type (WT), for each nucleotide (x-axis), for each tRNA isoacceptor (y-axis, ordered from most to least abundant in descending order) (**Table S17**). The positions of specific RNA modifications in each tRNA are listed in **Table S18**. Nucleotides with a higher summed basecalling error frequency relative to WT are in red tones, and those with a lower summed basecalling error frequency are in blue tones.

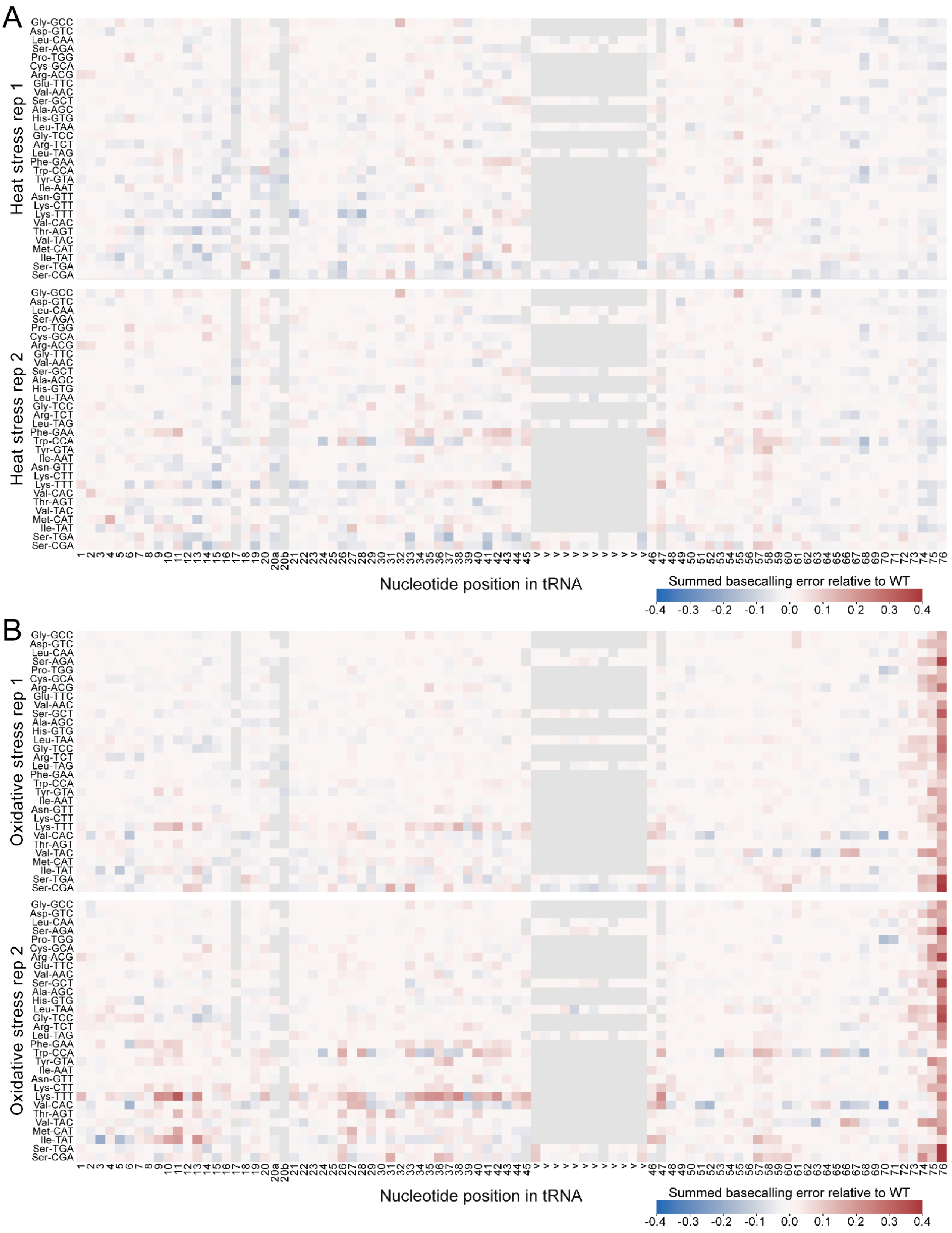

Supplementary Figure 3. IGV snapshots of *S. cerevisiae* tRNA$^{Ala(AGC)}$ and tRNA$^{Ala(TGC)}$. IGV tracks of 5' and 3' adapter-ligated tRNA$^{Ala(AGC)}$ and tRNA$^{Ala(UGC)}$ from WT and Pus4 KO *S. cerevisiae* strains and under heat stress and oxidative stress. In the upper coverage track, positions with a mismatch frequency greater than 0.2 are colored, whereas those showing mismatch frequencies lower than 0.2 are gray. The individual per-read data under the coverage track is not thresholded. For each condition (WT, Pus4 KO, heat stress, and oxidative stress), the upper panel corresponds to biological replicate 1, and the lower to biological replicate 2.

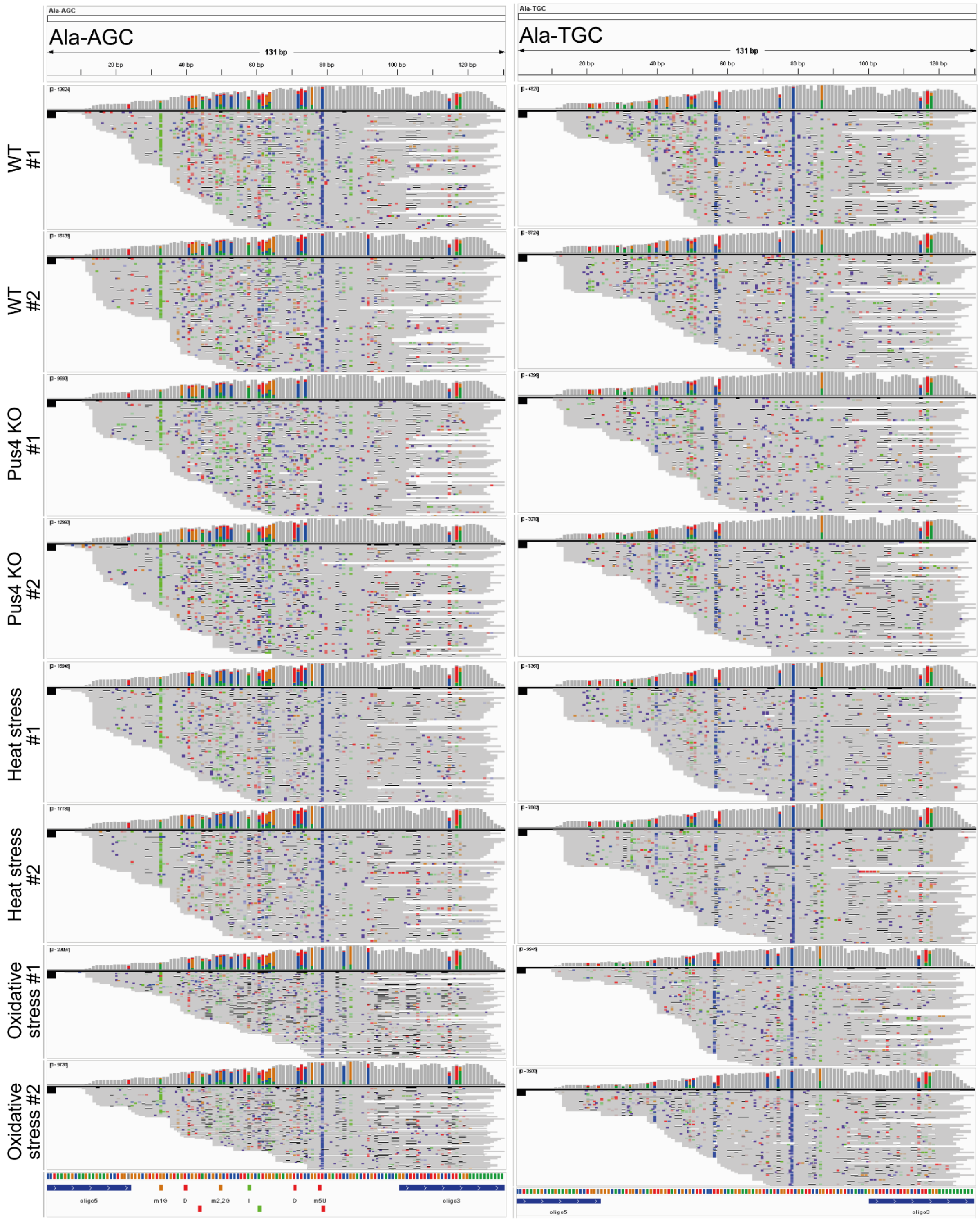

Supplementary Figure 4. IGV snapshots of *S. cerevisiae* tRNA$^{Arg(ACG)}$ and tRNA$^{Arg(CCG)}$. IGV tracks of 5' and 3' adapter ligated tRNA$^{Arg(ACG)}$ and tRNA$^{Arg(CCG)}$ from WT and Pus4 KO *S. cerevisiae* strains, and under heat stress and oxidative stress. In the upper coverage track, positions with a mismatch frequency greater than 0.2 are colored, whereas those showing mismatch frequencies lower than 0.2 are gray. The individual per-read data under the coverage track is not thresholded. For each condition (WT, Pus4 KO, heat stress, and oxidative stress), the upper panel corresponds to biological replicate 1, and the lower to biological replicate 2.

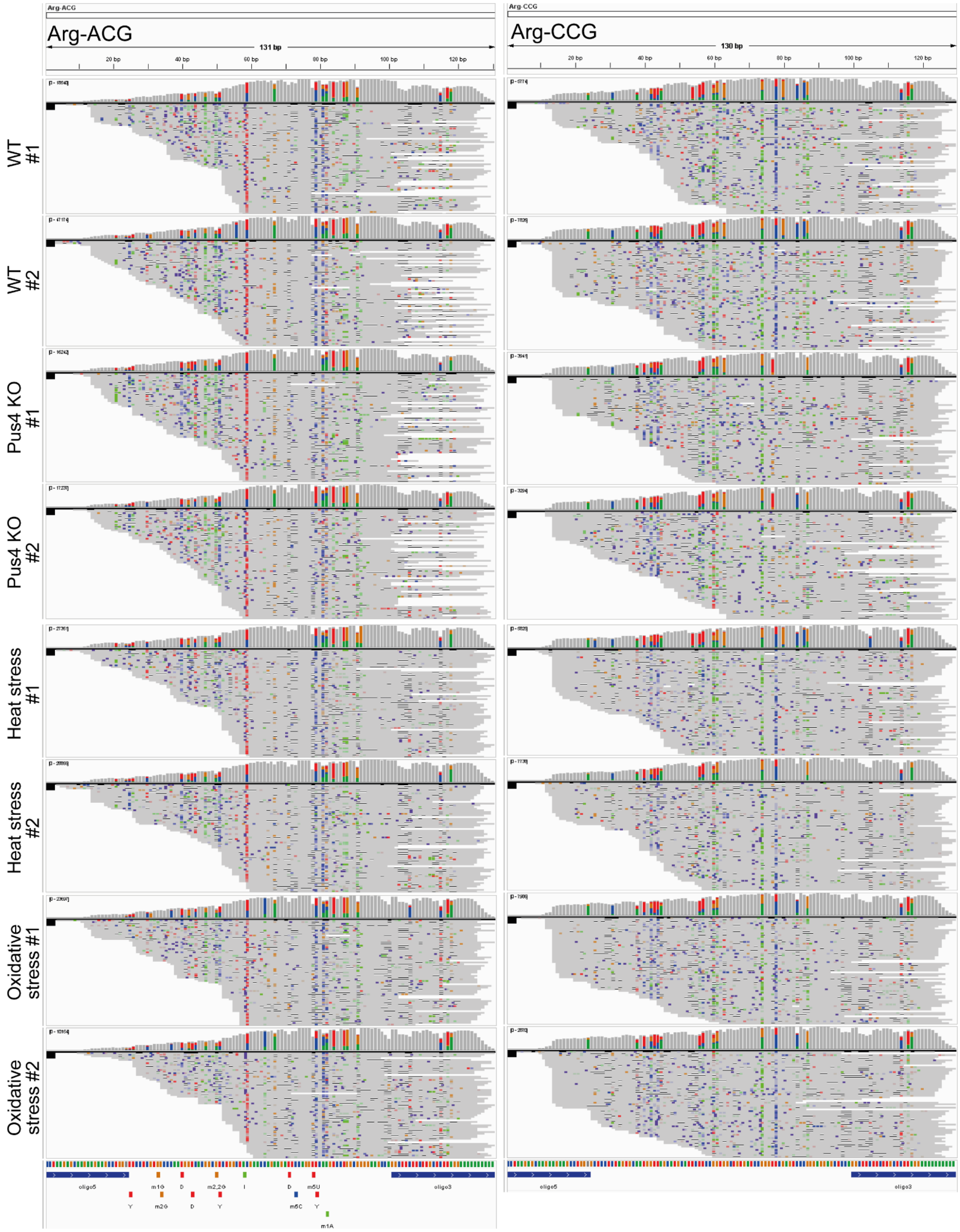

Supplementary Figure 5. IGV snapshots of *S. cerevisiae* tRNA$^{Arg(CCT)}$ and tRNA$^{Arg(TCT)}$. IGV tracks of 5' and 3' adapter ligated tRNA$^{Arg(CCT)}$ and tRNA$^{Arg(TCT)}$ from WT and Pus4 KO *S. cerevisiae* strains, and under heat stress and oxidative stress. In the upper coverage track, positions with a mismatch frequency greater than 0.2 are colored, whereas those showing mismatch frequencies lower than 0.2 are gray. The individual per-read data under the coverage track is not thresholded. For each condition (WT, Pus4 KO, heat stress, and oxidative stress), the upper panel corresponds to biological replicate 1, and the lower to biological replicate 2.

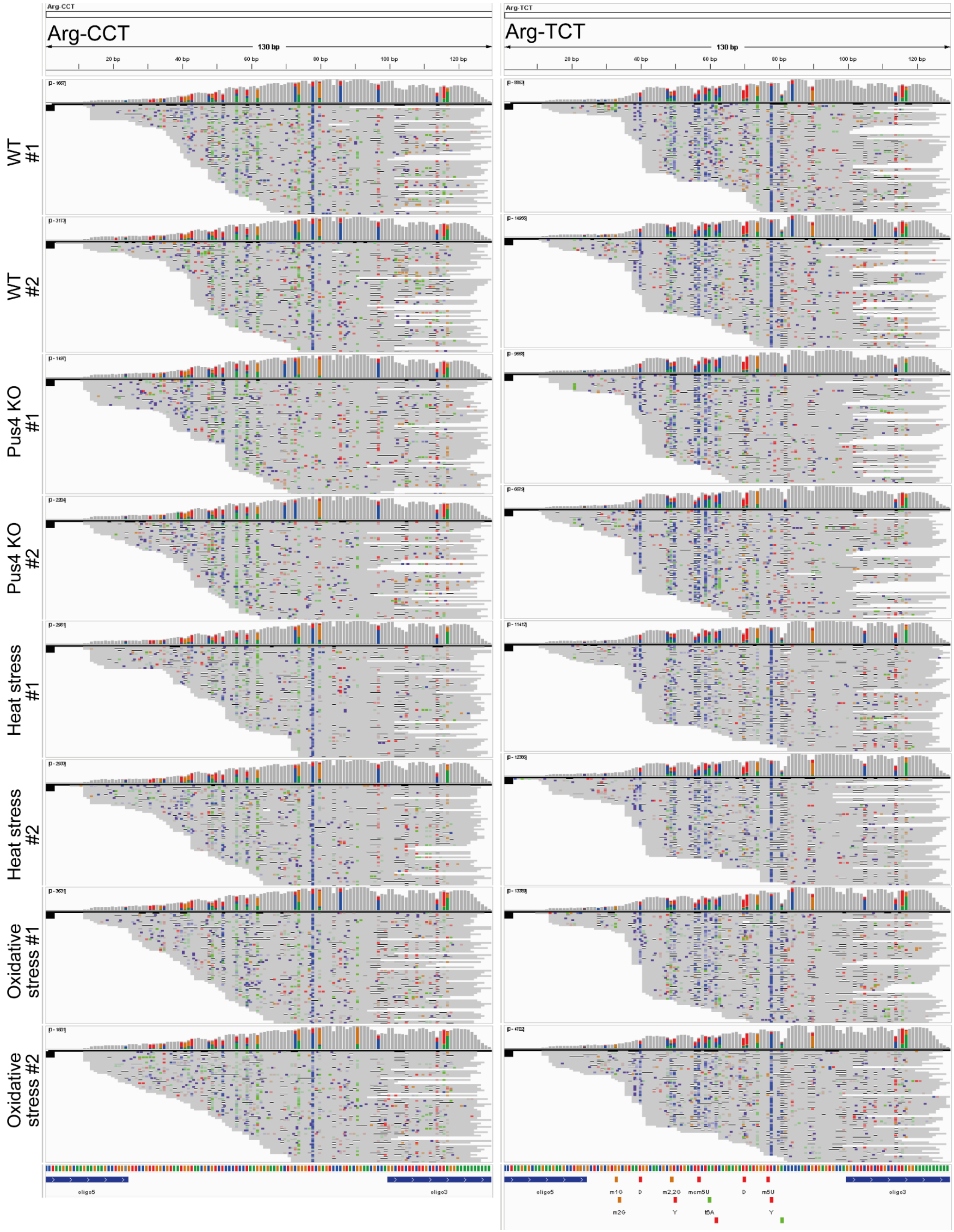

Supplementary Figure 6. IGV snapshots of *S. cerevisiae* tRNA$^{Asn(GTT)}$ and tRNA$^{Asp(GTC)}$. IGV tracks of 5' and 3' adapter-ligated tRNA$^{Asn(GTT)}$ and tRNA$^{Asp(GTC)}$ from WT and Pus4 KO *S. cerevisiae* strains and under heat stress and oxidative stress. In the upper coverage track, positions with a mismatch frequency greater than 0.2 are colored, whereas those showing mismatch frequencies lower than 0.2 are gray. The individual per-read data under the coverage track is not thresholded. For each condition (WT, Pus4 KO, heat stress, and oxidative stress), the upper panel corresponds to biological replicate 1, and the lower to biological replicate 2.

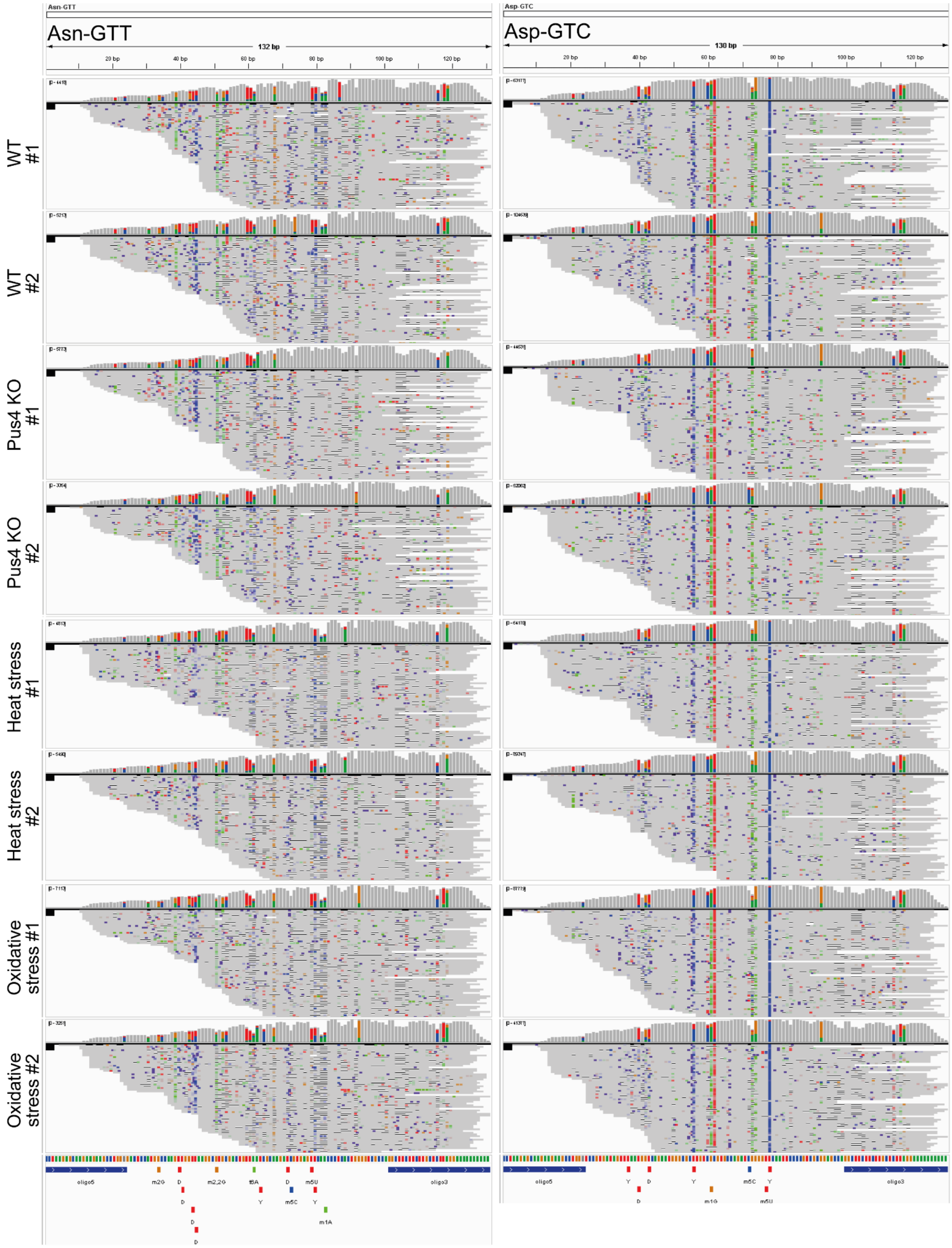

Supplementary Figure 7. IGV snapshots of *S. cerevisiae* tRNA$^{Cys(GCA)}$ and tRNA$^{Gln(GTC)}$. IGV tracks of 5' and 3' adapter-ligated tRNA$^{Cys(GCA)}$ and tRNA$^{Gln(GTC)}$ from WT and Pus4 KO *S. cerevisiae* strains and under heat stress and oxidative stress. In the upper coverage track, positions with a mismatch frequency greater than 0.2 are colored, whereas those showing mismatch frequencies lower than 0.2 are gray. The individual per-read data under the coverage track is not thresholded. For each condition (WT, Pus4 KO, heat stress, and oxidative stress), the upper panel corresponds to biological replicate 1, and the lower to biological replicate 2.

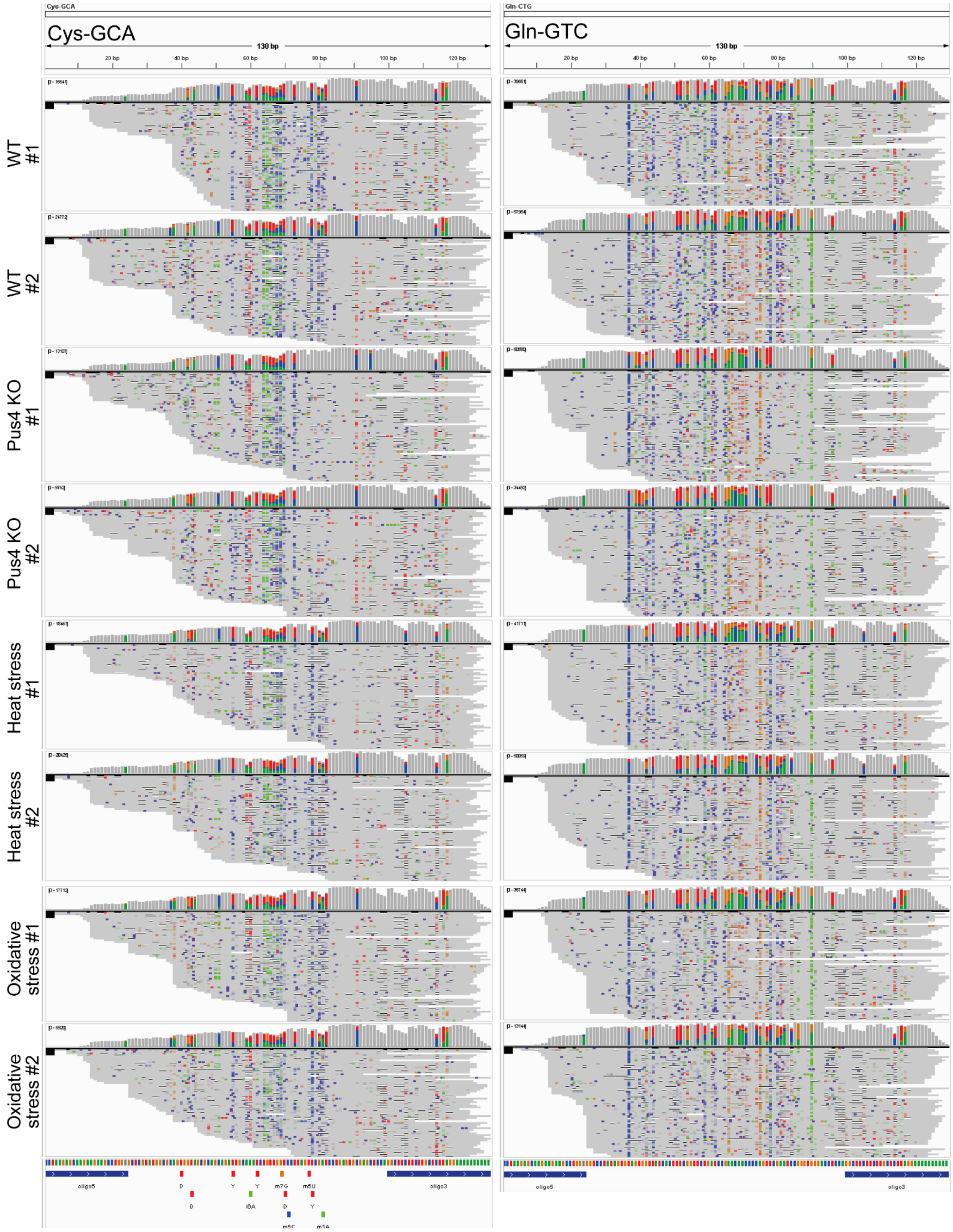

Supplementary Figure 8. IGV snapshots of *S. cerevisiae* tRNA$^{Gln(TTG)}$ and tRNA$^{Glu(CTC)}$. IGV tracks of 5' and 3' adapter-ligated tRNA$^{Gln(TTG)}$ and tRNA$^{Glu(CTC)}$ from WT and Pus4 KO *S. cerevisiae* strains and under heat stress and oxidative stress. In the upper coverage track, positions with a mismatch frequency greater than 0.2 are colored, whereas those showing mismatch frequencies lower than 0.2 are gray. The individual per-read data under the coverage track is not thresholded. For each condition (WT, Pus4 KO, heat stress, and oxidative stress), the upper panel corresponds to biological replicate 1, and the lower to biological replicate 2.

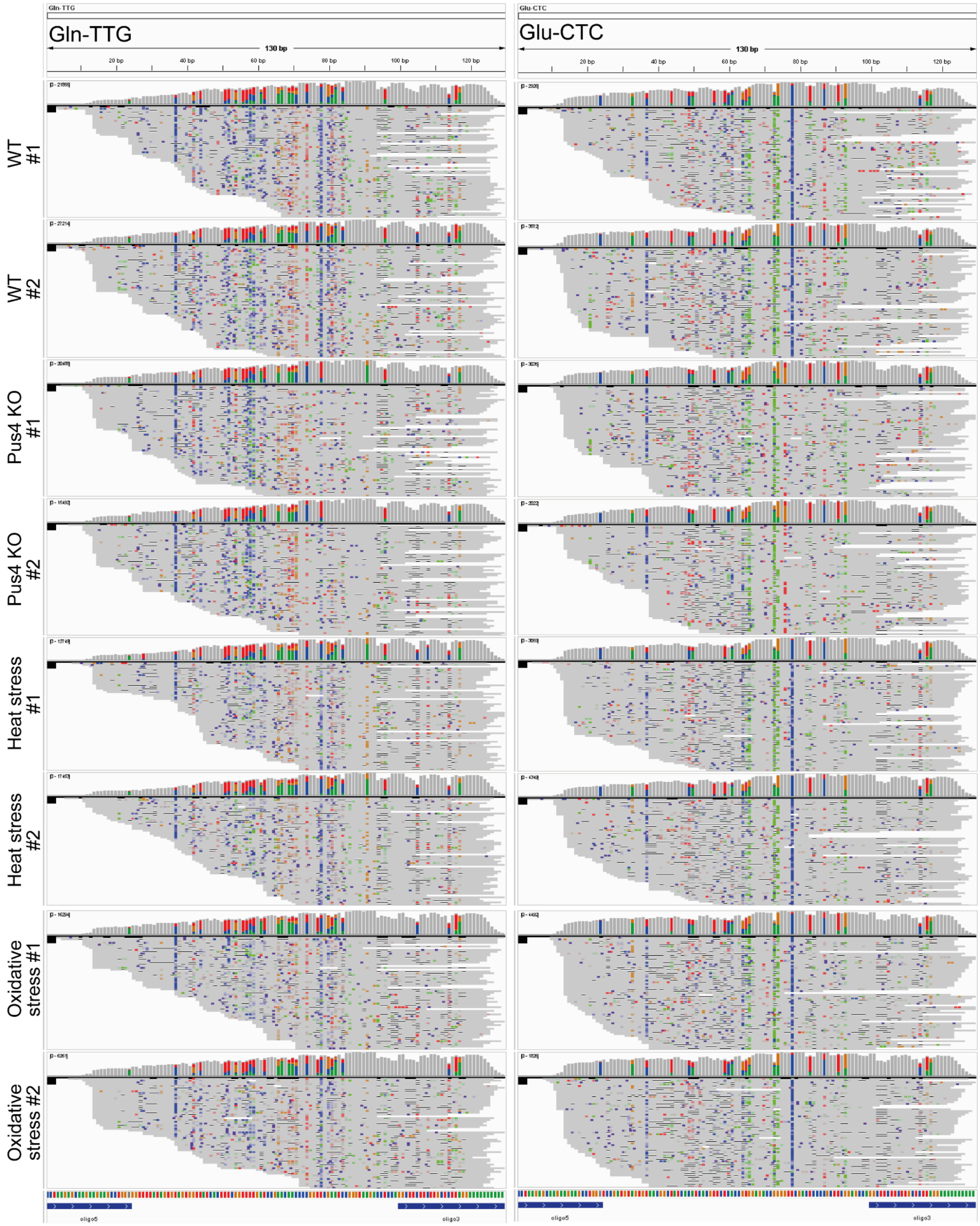

Supplementary Figure 9. IGV snapshots of *S. cerevisiae* tRNA$^{Glu(TTC)}$ and tRNA$^{Gly(CCC)}$. IGV tracks of 5' and 3' adapter-ligated tRNA$^{Glu(TTC)}$ and tRNA$^{Gly(CCC)}$ from WT and Pus4 KO *S. cerevisiae* strains and under heat stress and oxidative stress. In the upper coverage track, positions with a mismatch frequency greater than 0.2 are colored, whereas those showing mismatch frequencies lower than 0.2 are gray. The individual per-read data under the coverage track is not thresholded. For each condition (WT, Pus4 KO, heat stress, and oxidative stress), the upper panel corresponds to biological replicate 1, and the lower to biological replicate 2.

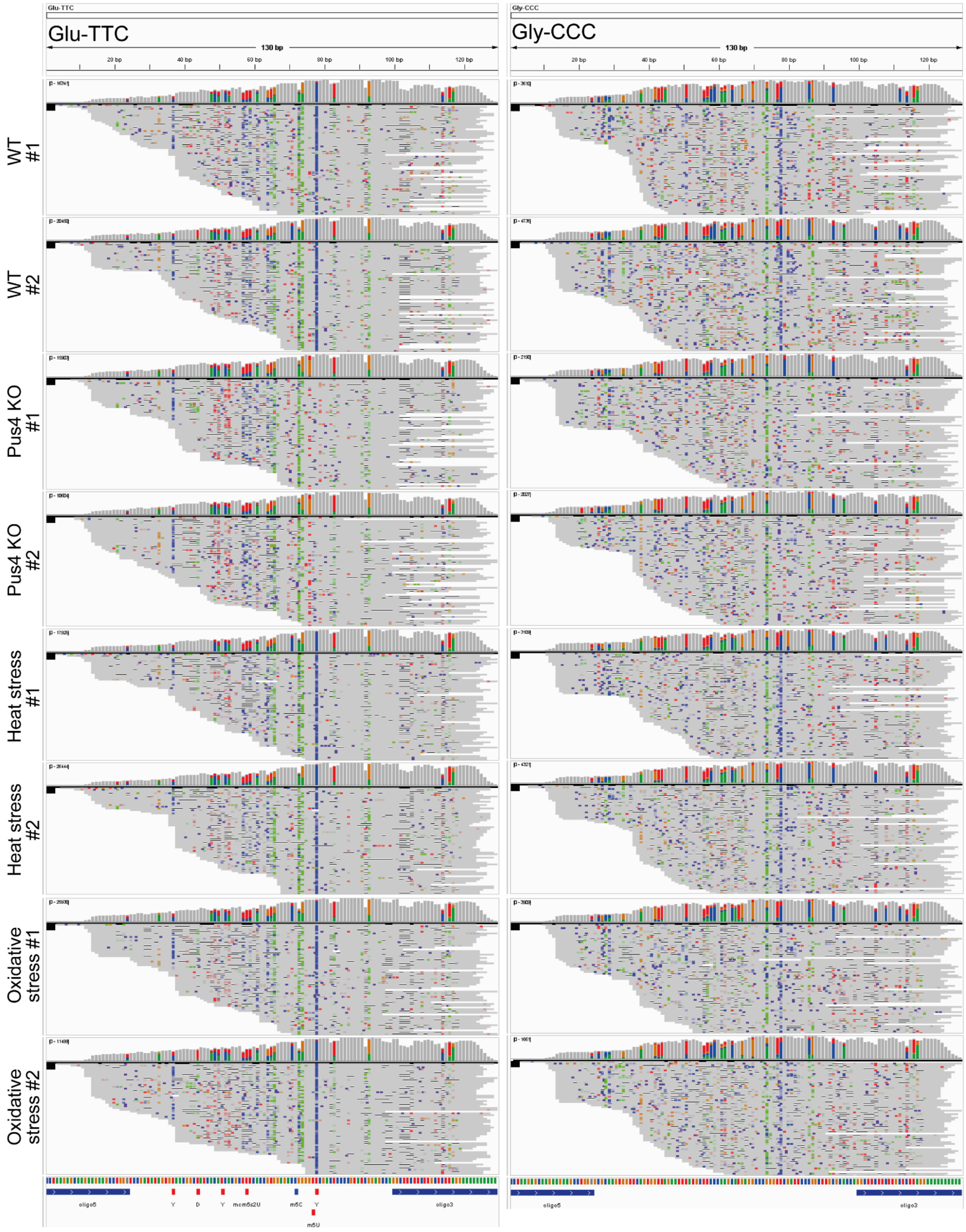

Supplementary Figure 10. IGV snapshots of *S. cerevisiae* tRNA$^{Gly(GCC)}$ and tRNA$^{Gly(TCC)}$. IGV tracks of 5' and 3' adapter-ligated tRNA$^{Gly(GCC)}$ and tRNA$^{Gly(TCC)}$ from WT and Pus4 KO *S. cerevisiae* strains and under heat stress and oxidative stress. In the upper coverage track, positions with a mismatch frequency greater than 0.2 are colored, whereas those showing mismatch frequencies lower than 0.2 are gray. The individual per-read data under the coverage track is not thresholded. For each condition (WT, Pus4 KO, heat stress, and oxidative stress), the upper panel corresponds to biological replicate 1, and the lower to biological replicate 2.

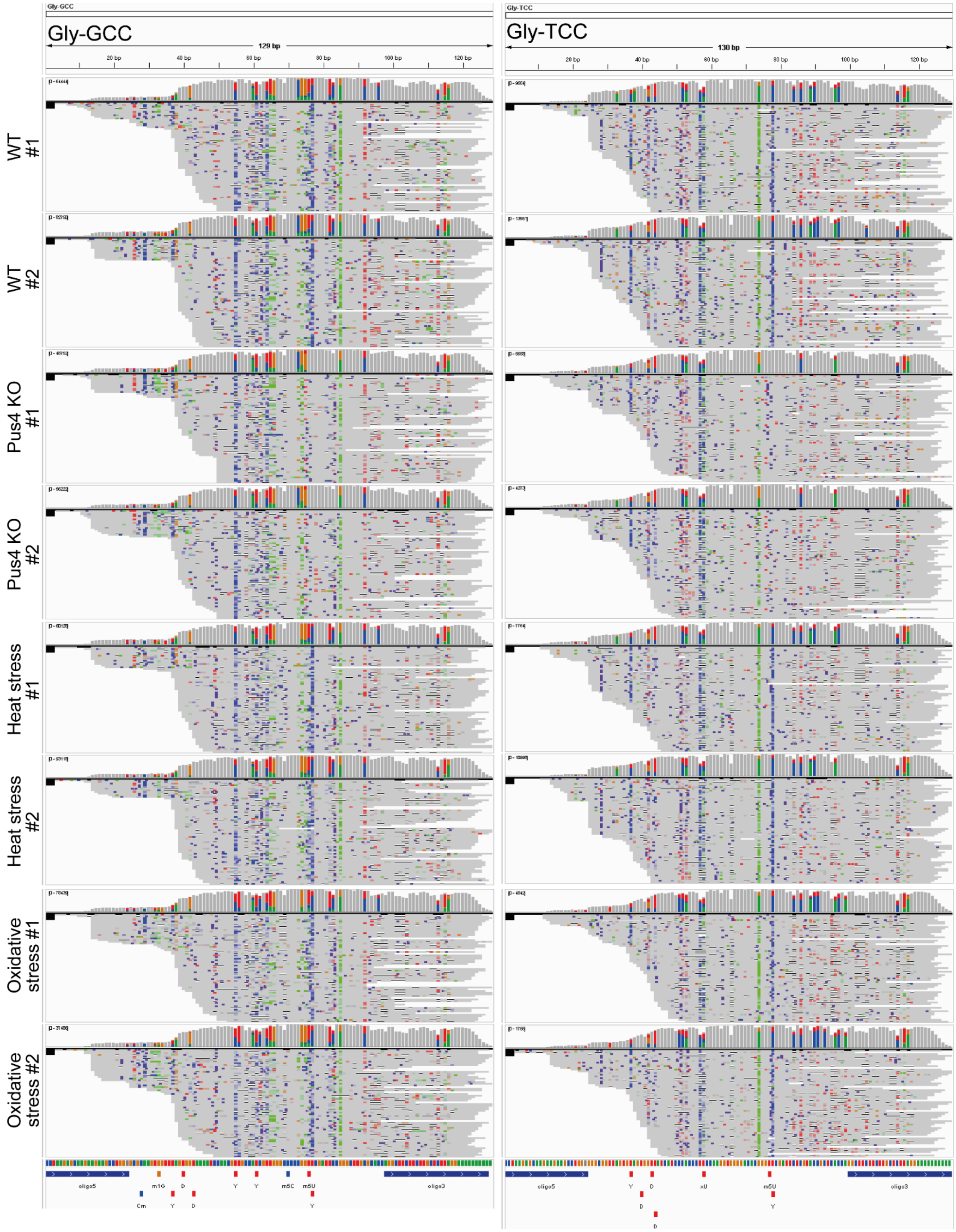

Supplementary Figure 11. IGV snapshots of *S. cerevisiae* tRNA$^{\text{His(GTG)}}$ and tRNA$^{\text{Ile(AAT)}}$. IGV tracks of 5' and 3' adapter-ligated **t**RNA$^{\text{His(GTG)}}$ and tRNA$^{\text{Ile(AAT)}}$ from WT and Pus4 KO *S. cerevisiae* strains and under heat stress and oxidative stress. In the upper coverage track, positions with a mismatch frequency greater than 0.2 are colored, whereas those showing mismatch frequencies lower than 0.2 are gray. The individual per-read data under the coverage track is not thresholded. For each condition (WT, Pus4 KO, heat stress, and oxidative stress), the upper panel corresponds to biological replicate 1, and the lower to biological replicate 2.

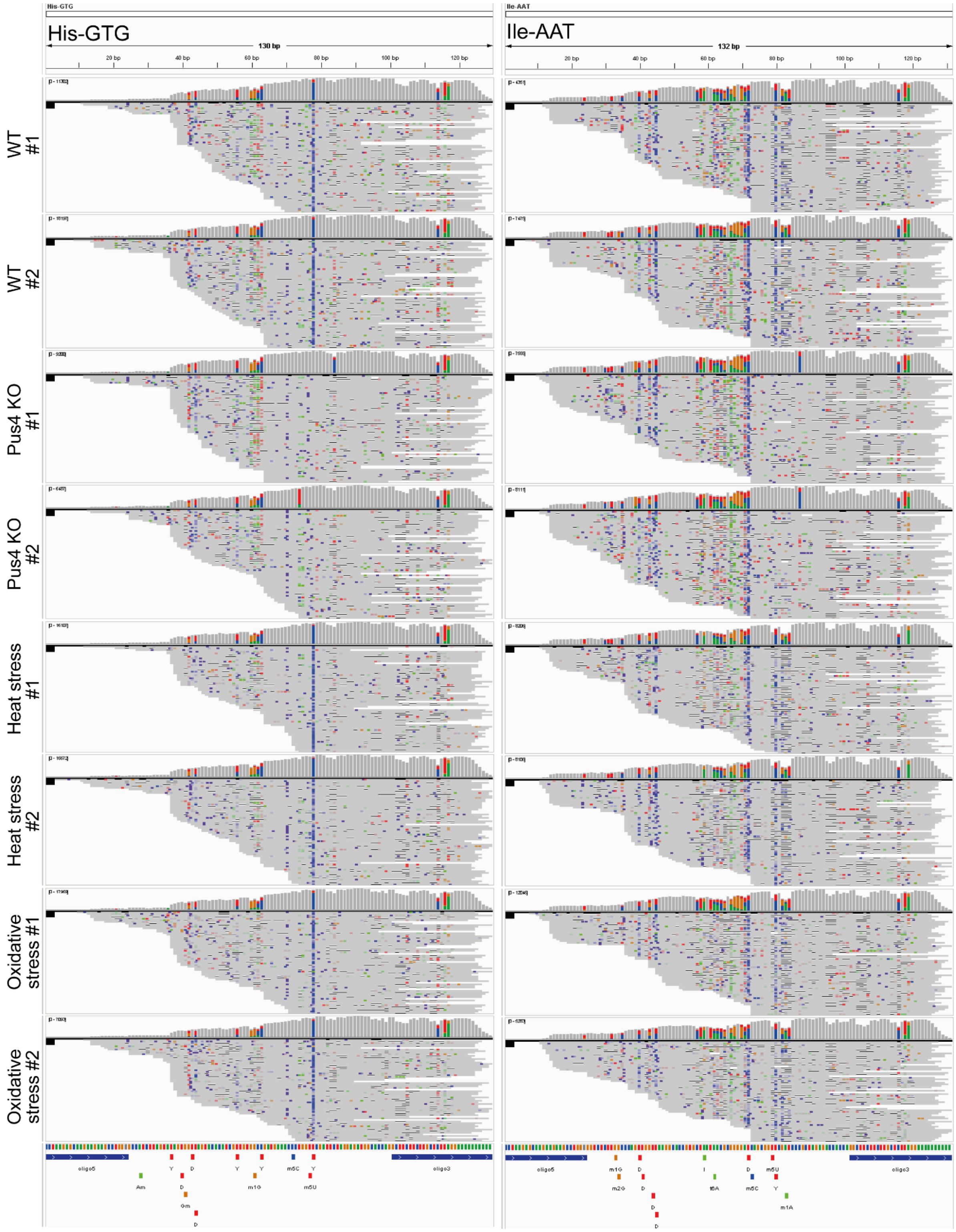

Supplementary Figure 12. IGV snapshots of *S. cerevisiae* tRNA$^{Ile(TAT)}$ and tRNA$^{iMet(CAT)}$. IGV tracks of 5’ and 3’ adapter-ligated tRNA$^{Ile(TAT)}$ and tRNA$^{iMet(CAT)}$ from WT and Pus4 KO *S. cerevisiae* strains and under heat stress and oxidative stress. In the upper coverage track, positions with a mismatch frequency greater than 0.2 are colored, whereas those showing mismatch frequencies lower than 0.2 are gray. The individual per-read data under the coverage track is not thresholded. For each condition (WT, Pus4 KO, heat stress, and oxidative stress), the upper panel corresponds to biological replicate 1, and the lower to biological replicate 2.

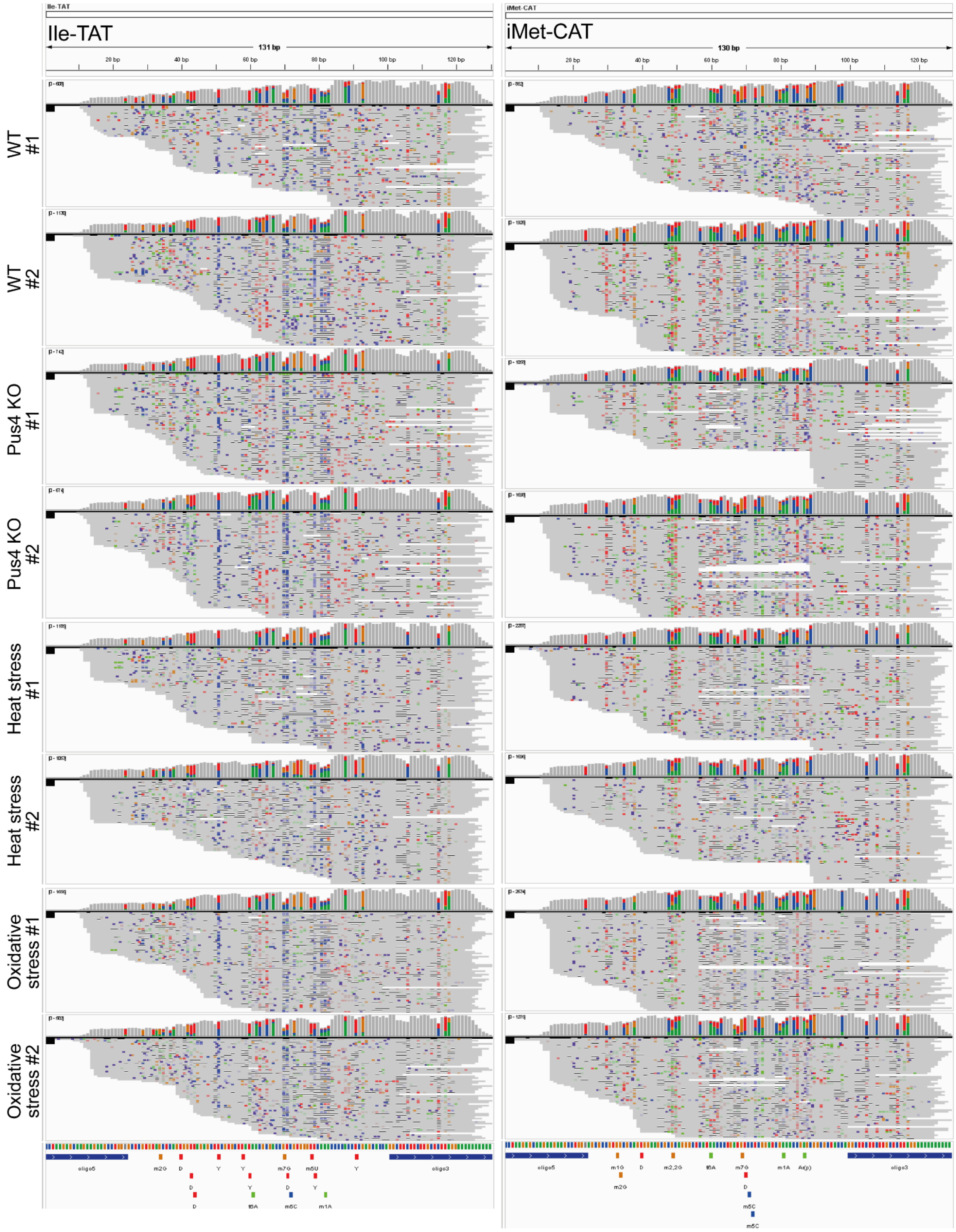

Supplementary Figure 13. IGV snapshots of *S. cerevisiae* tRNA$^{Leu(CAA)}$ and tRNA$^{Leu(GAG)}$. IGV tracks of 5' and 3' adapter-ligated tRNA$^{Leu(CAA)}$ and tRNA$^{Leu(GAG)}$ from WT and Pus4 KO *S. cerevisiae* strains and under heat stress and oxidative stress. In the upper coverage track, positions with a mismatch frequency greater than 0.2 are colored, whereas those showing mismatch frequencies lower than 0.2 are gray. The individual per-read data under the coverage track is not thresholded. For each condition (WT, Pus4 KO, heat stress, and oxidative stress), the upper panel corresponds to biological replicate 1, and the lower to biological replicate 2.

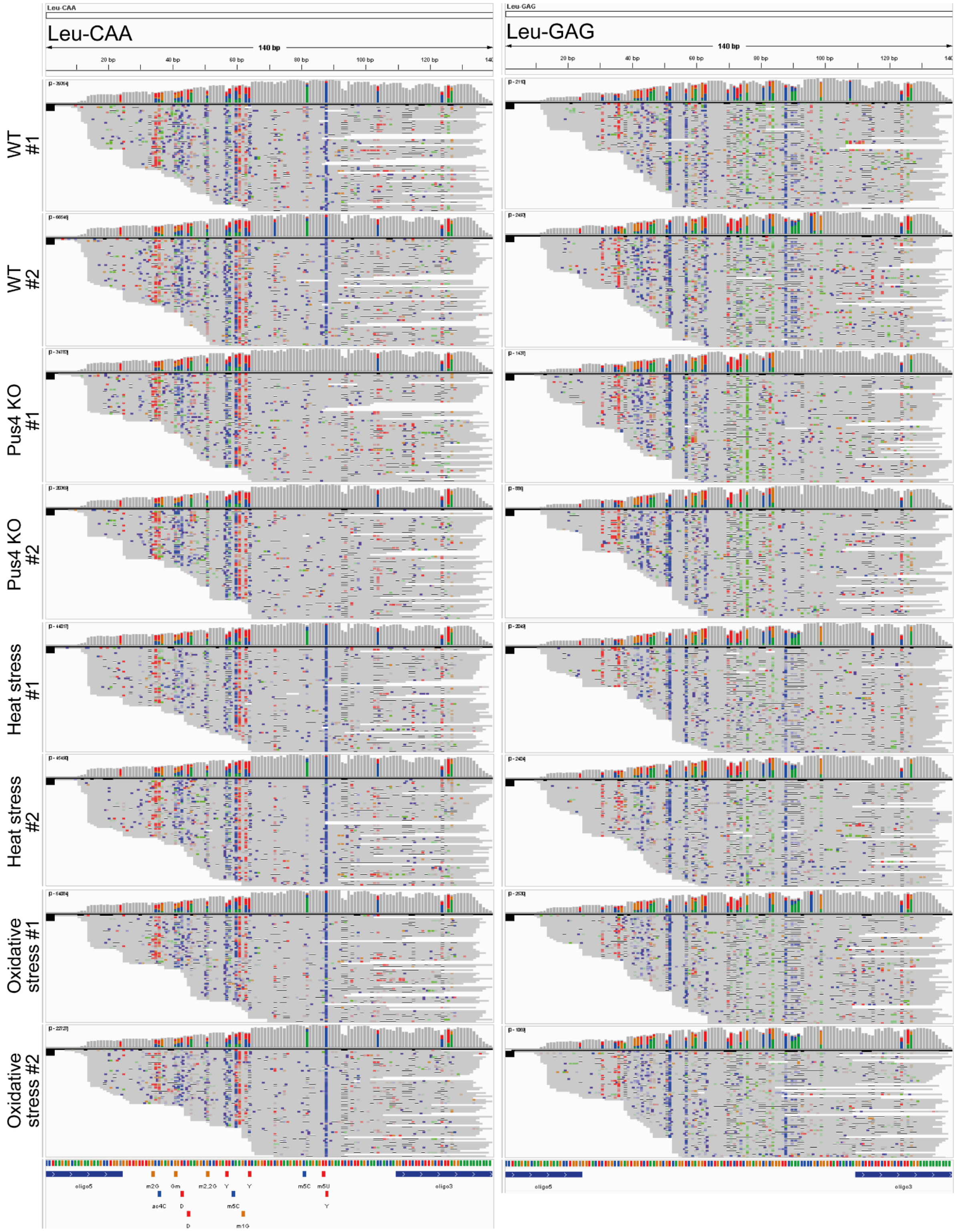

Supplementary Figure 14. IGV snapshots of *S. cerevisiae* tRNA$^{Leu(TAA)}$ and tRNA$^{Leu(TAG)}$. IGV tracks of 5' and 3' adapter-ligated tRNA$^{Leu(TAA)}$ and tRNA$^{Leu(TAG)}$ from WT and Pus4 KO *S. cerevisiae* strains and under heat stress and oxidative stress. In the upper coverage track, positions with a mismatch frequency greater than 0.2 are colored, whereas those showing mismatch frequencies lower than 0.2 are gray. The individual per-read data under the coverage track is not thresholded. For each condition (WT, Pus4 KO, heat stress, and oxidative stress), the upper panel corresponds to biological replicate 1, and the lower to biological replicate 2.

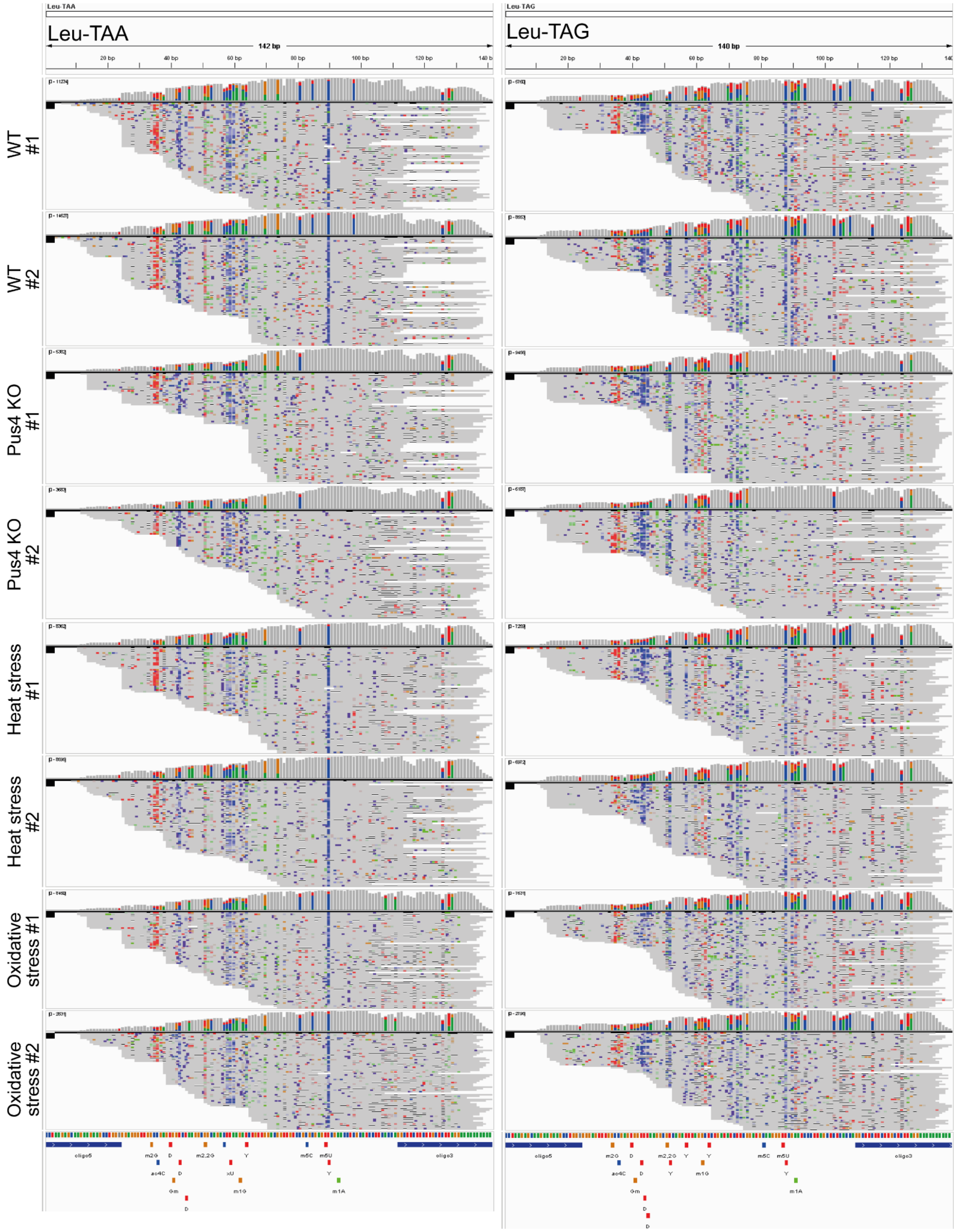

Supplementary Figure 15. IGV snapshots of *S. cerevisiae* tRNA$^{Lys(CTT)}$ and tRNA$^{Lys(TTT)}$. IGV tracks of 5' and 3' adapter-ligated tRNA$^{Lys(CTT)}$ and tRNA$^{Lys(TTT)}$ from WT and Pus4 KO *S. cerevisiae* strains and under heat stress and oxidative stress. In the upper coverage track, positions with a mismatch frequency greater than 0.2 are colored, whereas those showing mismatch frequencies lower than 0.2 are gray. The individual per-read data under the coverage track is not thresholded. For each condition (WT, Pus4 KO, heat stress, and oxidative stress), the upper panel corresponds to biological replicate 1, and the lower to biological replicate 2.

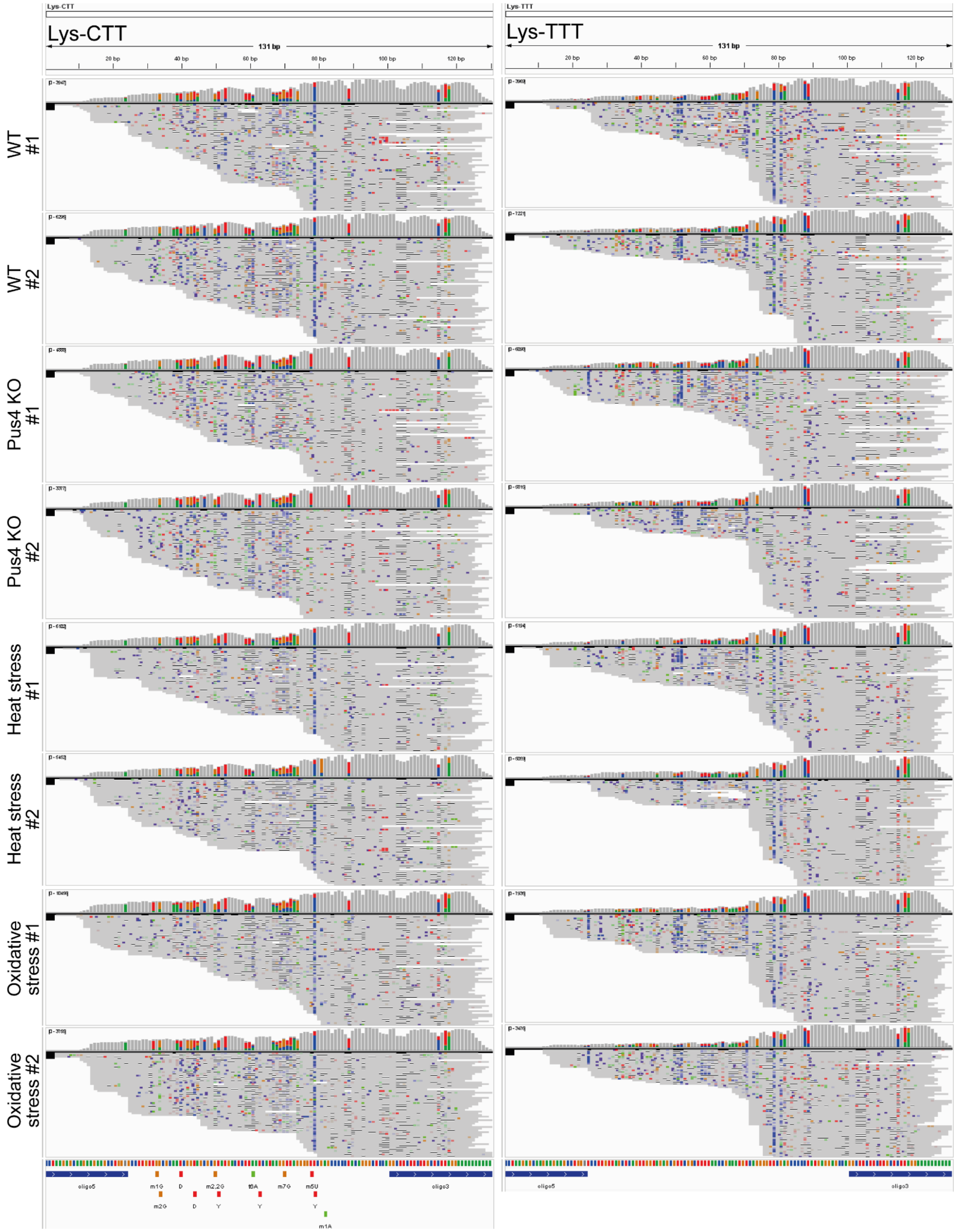

Supplementary Figure 16. IGV snapshots of *S. cerevisiae* tRNA$^{Met(CAT)}$ and tRNA$^{Phe(GAA)}$. IGV tracks of 5' and 3' adapter-ligated tRNA$^{Met(CAT)}$ and tRNA$^{Phe(GAA)}$ from WT and Pus4 KO *S. cerevisiae* strains and under heat stress and oxidative stress. In the upper coverage track, positions with a mismatch frequency greater than 0.2 are colored, whereas those showing mismatch frequencies lower than 0.2 are gray. The individual per-read data under the coverage track is not thresholded. For each condition (WT, Pus4 KO, heat stress, and oxidative stress), the upper panel corresponds to biological replicate 1, and the lower to biological replicate 2.

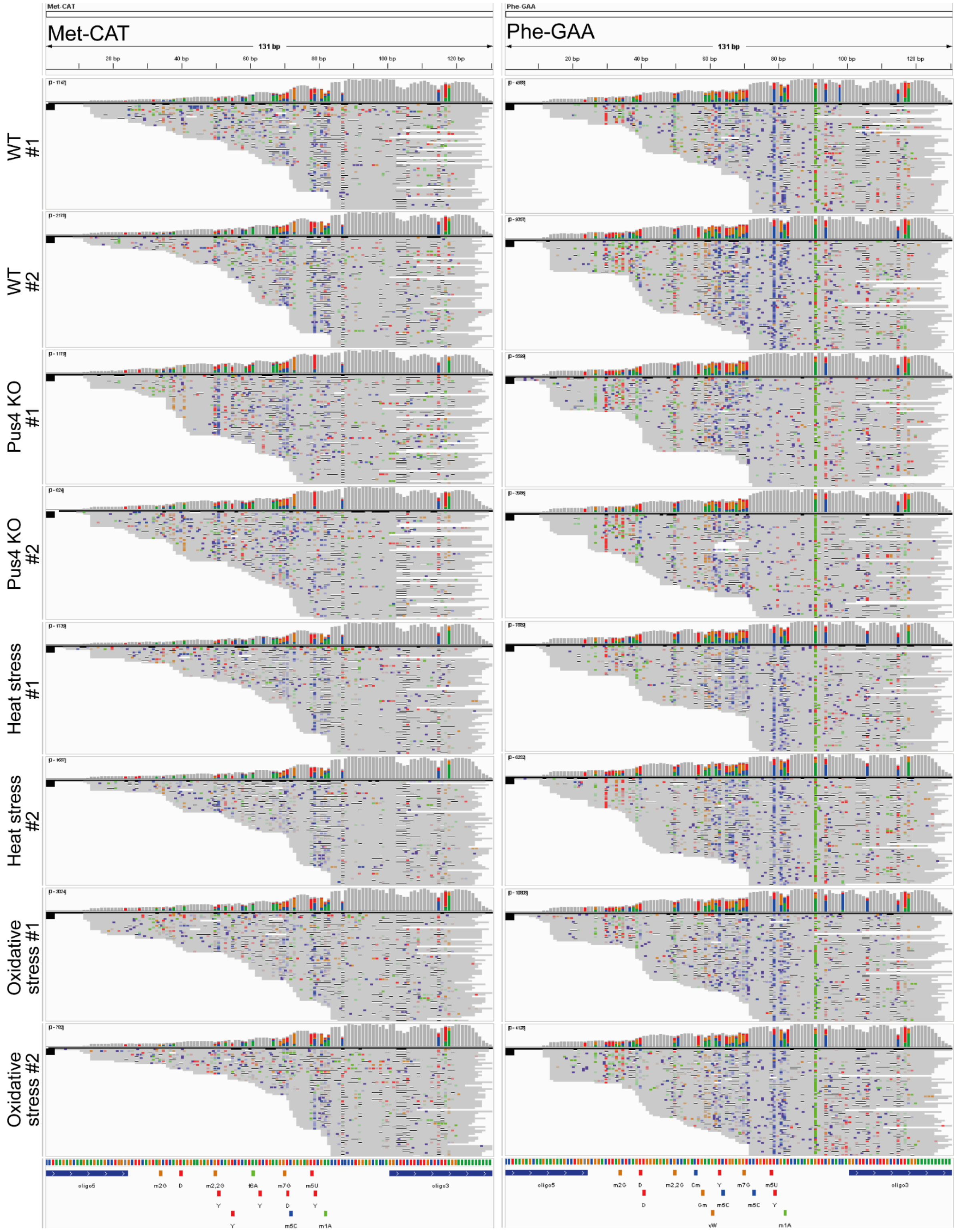

Supplementary Figure 17. IGV snapshots of *S. cerevisiae* tRNA$^{Pro(AGG)}$ and tRNA$^{Pro(TGG)}$. IGV tracks of 5' and 3' adapter-ligated tRNA$^{Pro(AGG)}$ and tRNA$^{Pro(TGG)}$ from WT and Pus4 KO *S. cerevisiae* strains and under heat stress and oxidative stress. In the upper coverage track, positions with a mismatch frequency greater than 0.2 are colored, whereas those showing mismatch frequencies lower than 0.2 are gray. The individual per-read data under the coverage track is not thresholded. For each condition (WT, Pus4 KO, heat stress, and oxidative stress), the upper panel corresponds to biological replicate 1, and the lower to biological replicate 2.

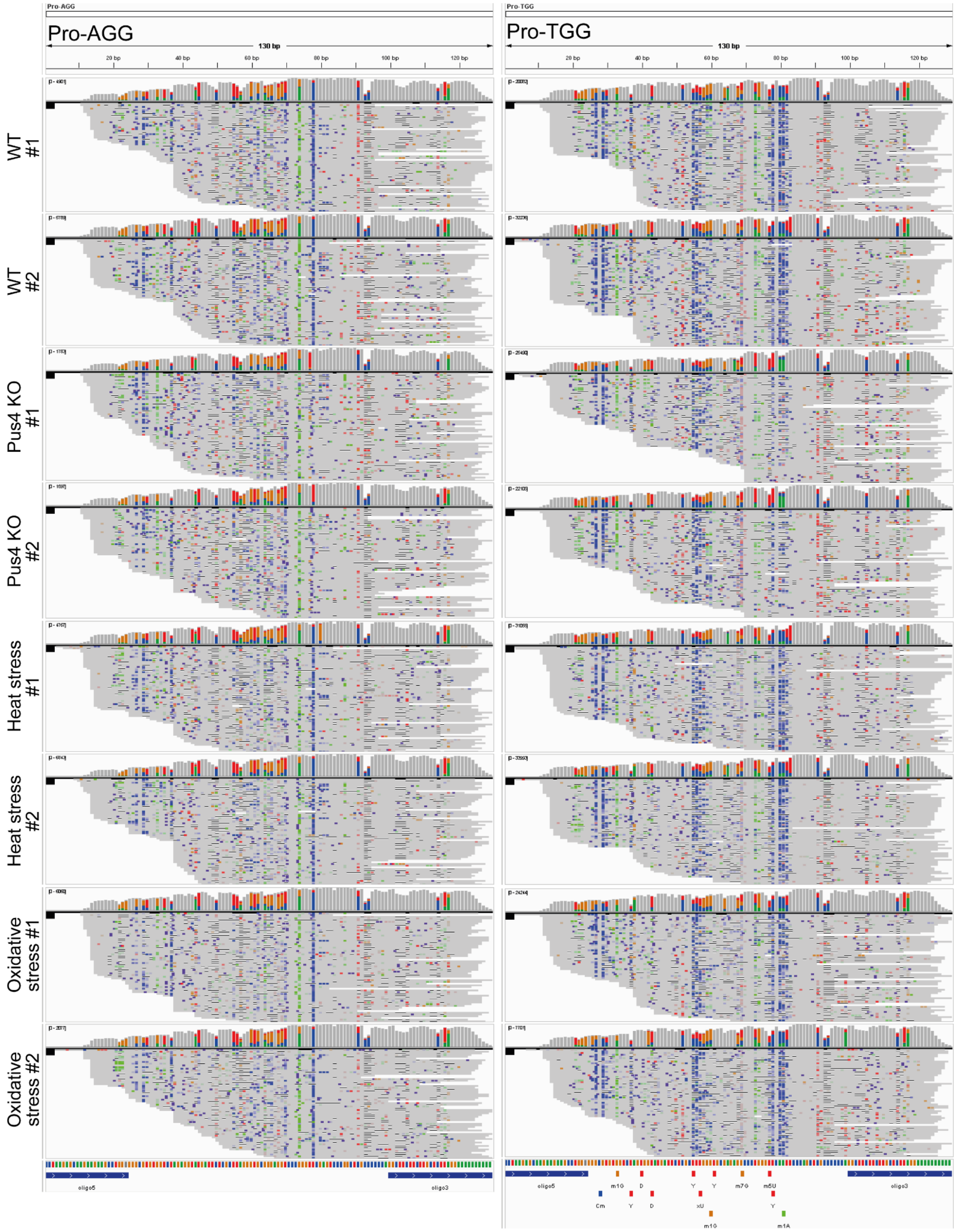

Supplementary Figure 18. IGV snapshots of *S. cerevisiae* tRNA$^{Ser(AGA)}$ and tRNA$^{Ser(CGA)}$. IGV tracks of 5' and 3' adapter-ligated tRNA$^{Ser(AGA)}$ and tRNA$^{Ser(CGA)}$ from WT and Pus4 KO *S. cerevisiae* strains and under heat stress and oxidative stress. In the upper coverage track, positions with a mismatch frequency greater than 0.2 are colored, whereas those showing mismatch frequencies lower than 0.2 are gray. The individual per-read data under the coverage track is not thresholded. For each condition (WT, Pus4 KO, heat stress, and oxidative stress), the upper panel corresponds to biological replicate 1, and the lower to biological replicate 2.

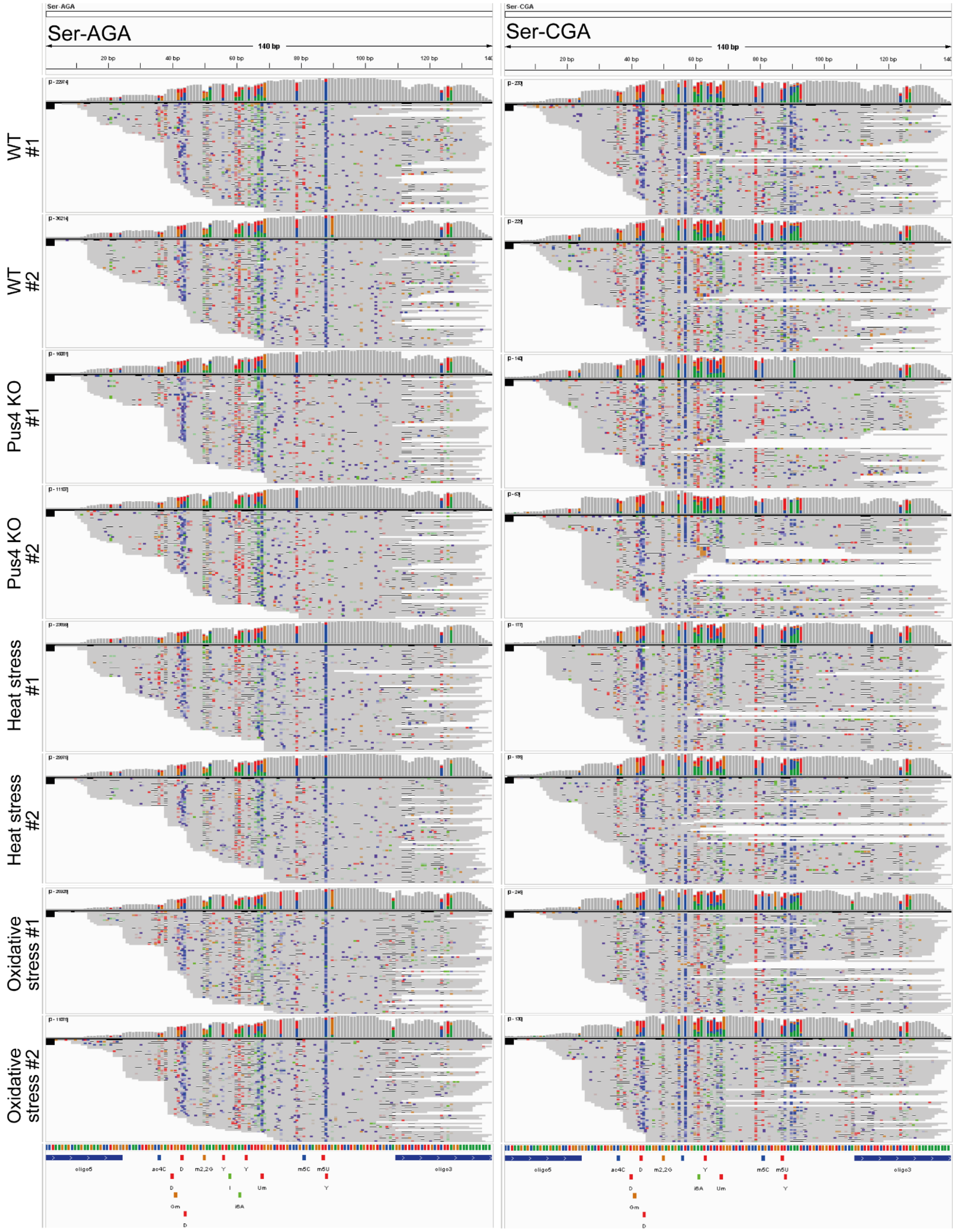

Supplementary Figure 19. IGV snapshots of *S. cerevisiae* tRNA$^{Ser(GCT)}$ and tRNA$^{Ser(TGA)}$. IGV tracks of 5' and 3' adapter-ligated tRNA$^{Ser(GCT)}$ and tRNA$^{Ser(TGA)}$ from WT and Pus4 KO *S. cerevisiae* strains and under heat stress and oxidative stress. In the upper coverage track, positions with a mismatch frequency greater than 0.2 are colored, whereas those showing mismatch frequencies lower than 0.2 are gray. The individual per-read data under the coverage track is not thresholded. For each condition (WT, Pus4 KO, heat stress, and oxidative stress), the upper panel corresponds to biological replicate 1, and the lower to biological replicate 2.

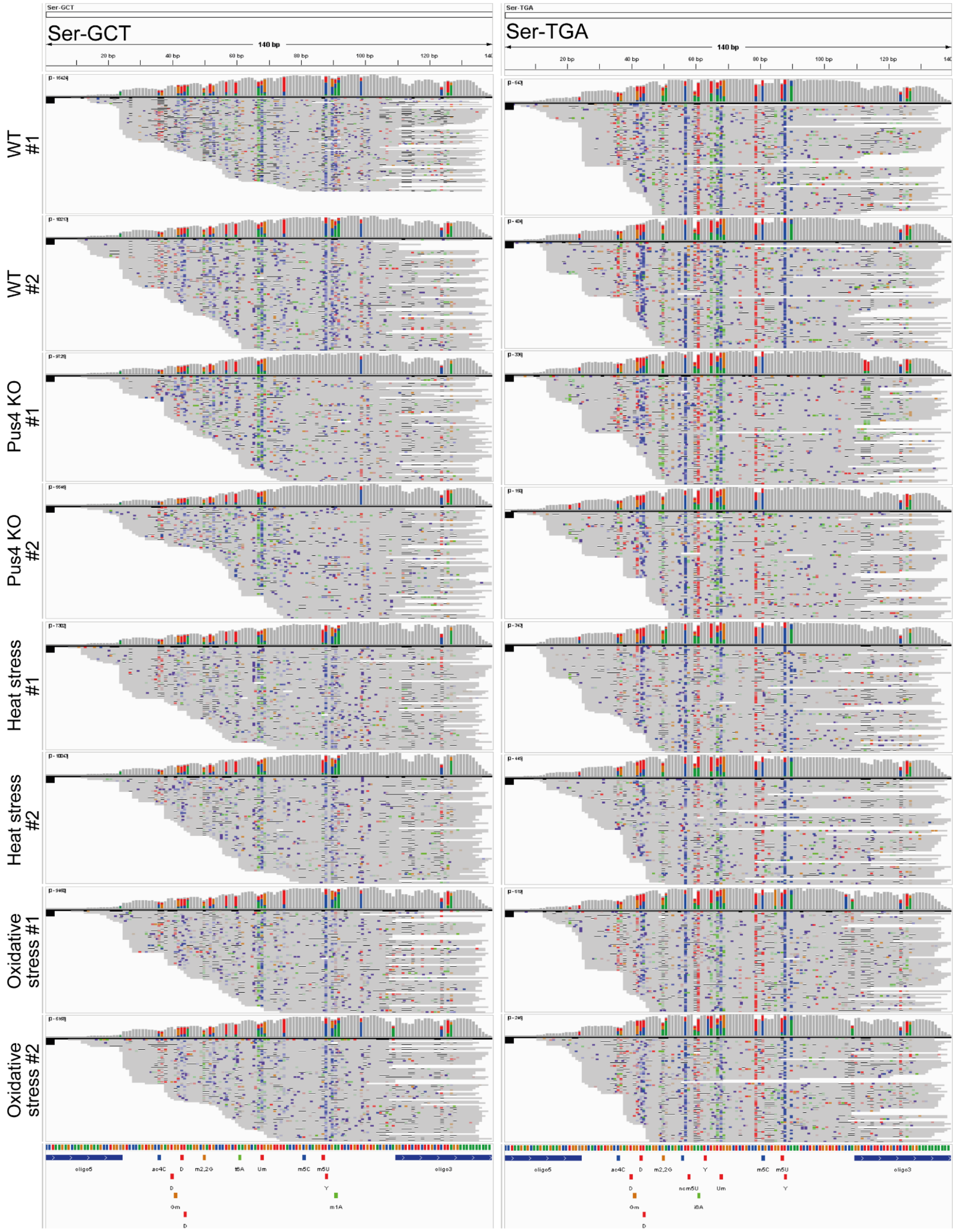

Supplementary Figure 20. IGV snapshots of *S. cerevisiae* tRNA$^{Thr(AGT)}$ and tRNA$^{Thr(CGT)}$. IGV tracks of 5' and 3' adapter-ligated tRNA$^{Thr(AGT)}$ and tRNA$^{The(CGT)}$ from WT and Pus4 KO *S. cerevisiae* strains and under heat stress and oxidative stress. In the upper coverage track, positions with a mismatch frequency greater than 0.2 are colored, whereas those showing mismatch frequencies lower than 0.2 are gray. The individual per-read data under the coverage track is not thresholded. For each condition (WT, Pus4 KO, heat stress, and oxidative stress), the upper panel corresponds to biological replicate 1, and the lower to biological replicate 2.

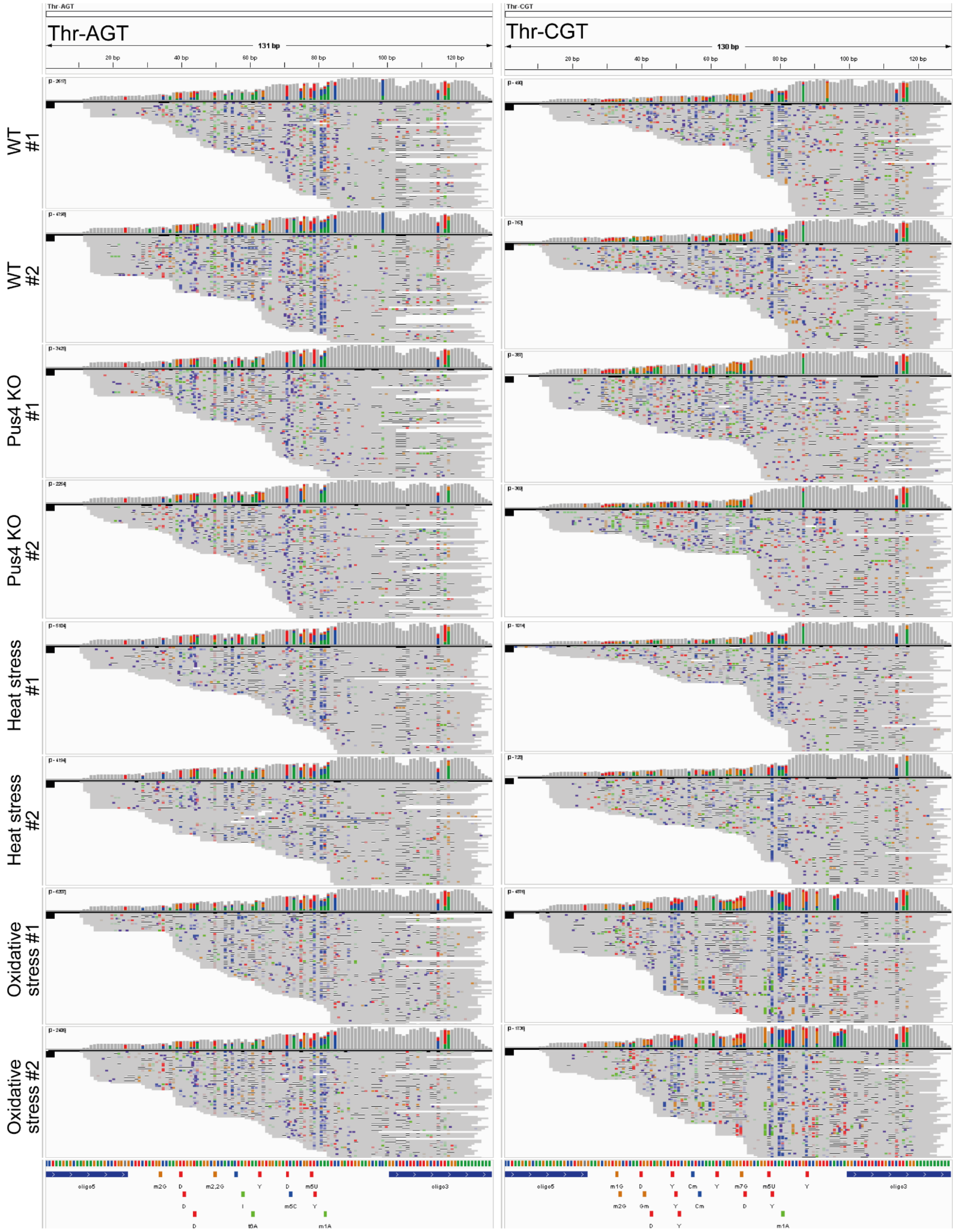

Supplementary Figure 21. IGV snapshots of *S. cerevisiae* tRNA$^{Thr(TGT)}$ and tRNA$^{Thr(CCA)}$. IGV tracks of 5' and 3' adapter-ligated tRNA$^{Thr(TGT)}$ and tRNA$^{Thr(CCA)}$ from WT and Pus4 KO *S. cerevisiae* strains and under heat stress and oxidative stress. In the upper coverage track, positions with a mismatch frequency greater than 0.2 are colored, whereas those showing mismatch frequencies lower than 0.2 are gray. The individual per-read data under the coverage track is not thresholded. For each condition (WT, Pus4 KO, heat stress, and oxidative stress), the upper panel corresponds to biological replicate 1, and the lower to biological replicate 2.

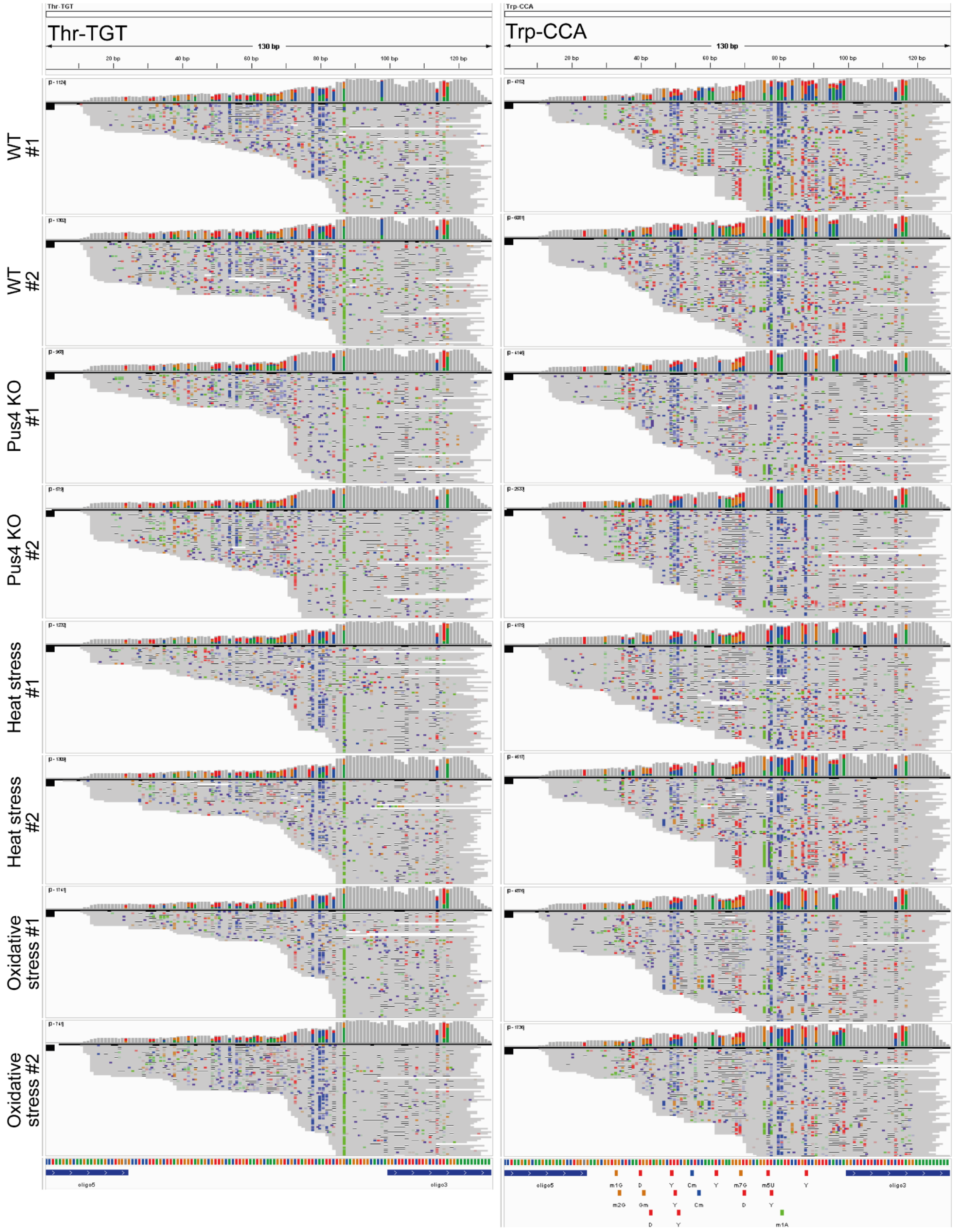

Supplementary Figure 22. IGV snapshots of *S. cerevisiae* tRNA$^{Tyr(GTA)}$ and tRNA$^{Val(AAC)}$. IGV tracks of 5' and 3' adapter-ligated tRNA$^{Tyr(GTA)}$ and tRNA$^{Val(AAC)}$ from WT and Pus4 KO *S. cerevisiae* strains and under heat stress and oxidative stress. In the upper coverage track, positions with a mismatch frequency greater than 0.2 are colored, whereas those showing mismatch frequencies lower than 0.2 are gray. The individual per-read data under the coverage track is not thresholded. For each condition (WT, Pus4 KO, heat stress, and oxidative stress), the upper panel corresponds to biological replicate 1, and the lower to biological replicate 2.

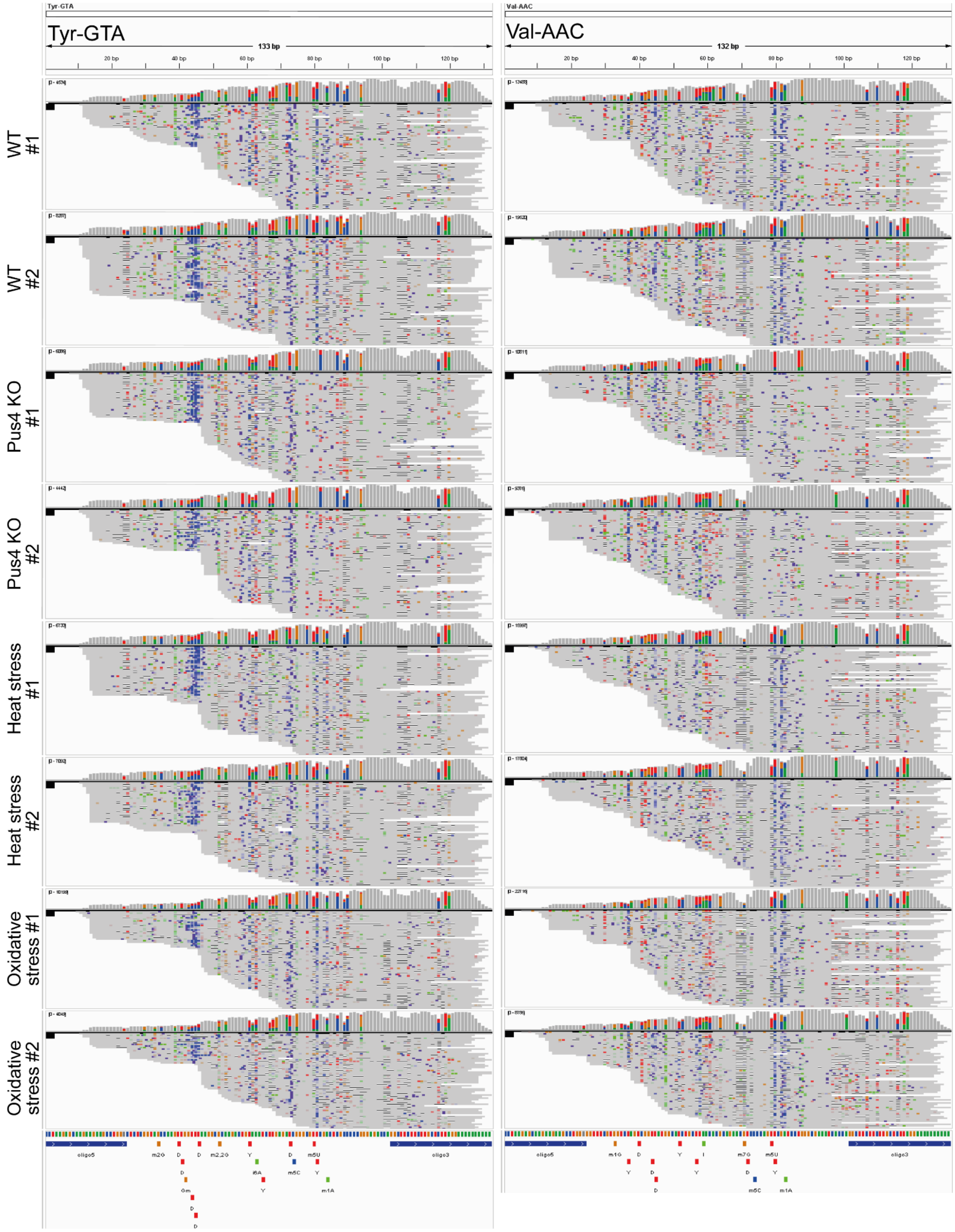

Supplementary Figure 23. IGV snapshots of *S. cerevisiae* tRNA$^{Val(CAC)}$ and tRNA$^{Val(TAC)}$. IGV tracks of 5' and 3' adapter-ligated tRNA$^{Val(CAC)}$ and tRNA$^{Val(TAC)}$ from WT and Pus4 KO *S. cerevisiae* strains and under heat stress and oxidative stress. In the upper coverage track, positions with a mismatch frequency greater than 0.2 are colored, whereas those showing mismatch frequencies lower than 0.2 are gray. The individual per-read data under the coverage track is not thresholded. For each condition (WT, Pus4 KO, heat stress, and oxidative stress), the upper panel corresponds to biological replicate 1, and the lower to biological replicate 2.

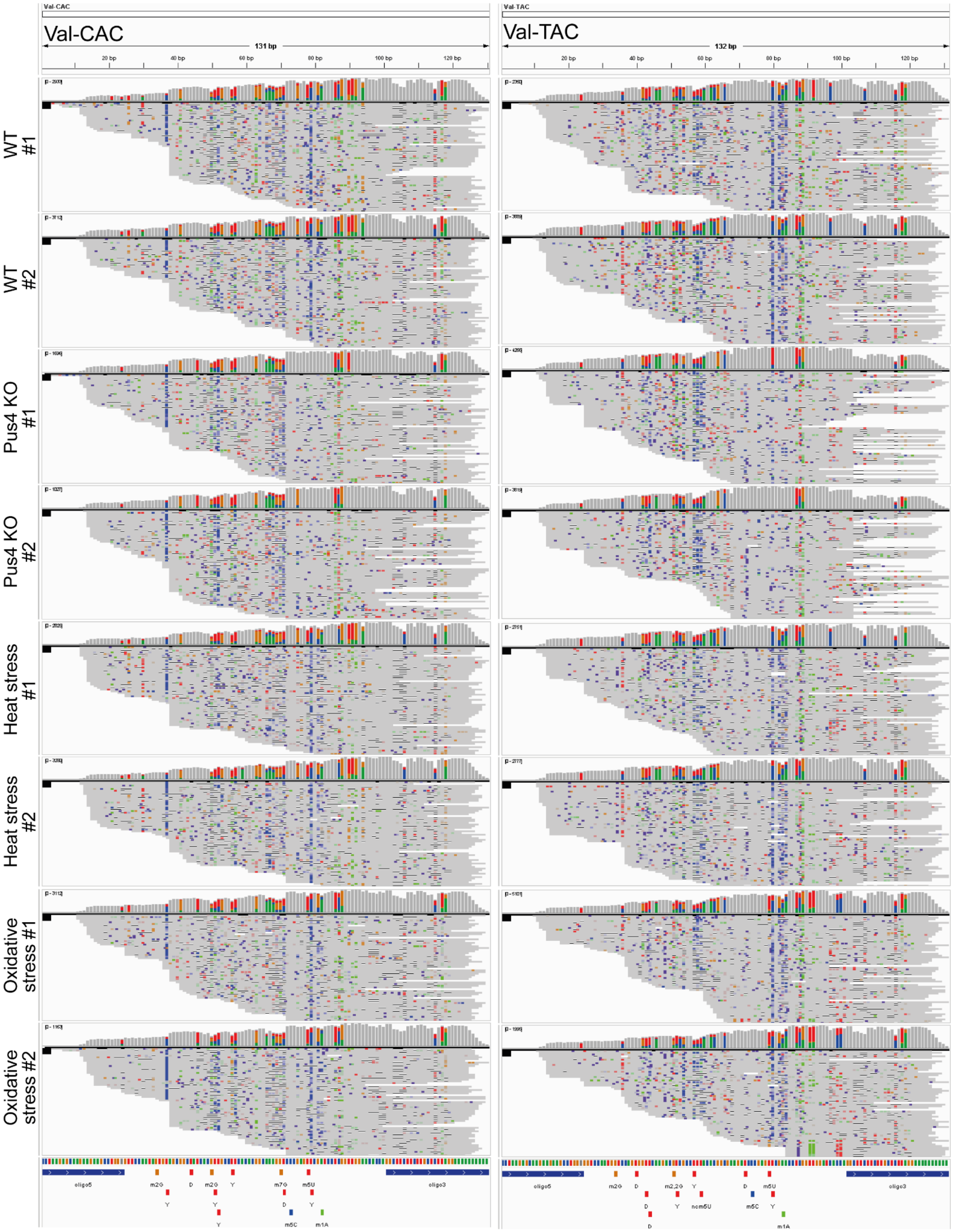

Supplementary Figure 24. Quality and purity of yeast RNA samples used in this study. (A) TapeStation profiles of wild-type (WT), Pus4 knockout (KO), and stressed *S. cerevisiae* tRNA-enriched 17-200 nt RNA fractions in triplicate after RNA extraction and **(B)** deacylation. **(C)** Images of gels used to purify and extract tRNAs for LC-MS/MS. Commercial *S. cerevisiae* $tRNA^{Phe}$ and total tRNA were used as markers, and samples were interspaced by an empty to avoid cross-contamination. tRNAs (~70-110 nt) correspond to the lowest row of the sample, rRNA 5s (~121 nt) to the middle, and rRNA 5.8s (~158 nt) to the highest. **(D)** TapeStation profiles of the tRNAs excised from the gels in (C).

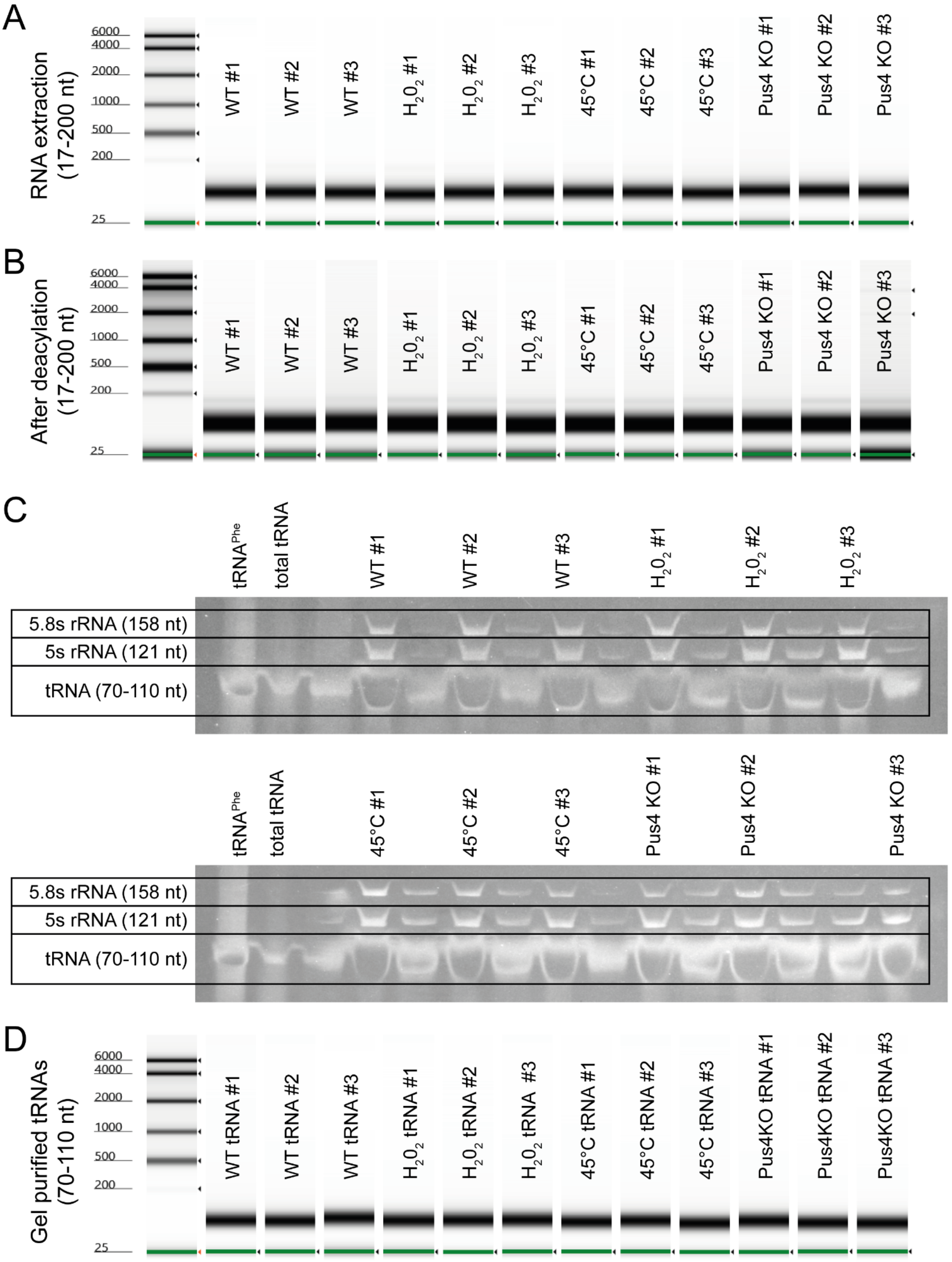

Supplementary Figure 25. Purity of in vitro transcribed tRNAs used in this study. TBE-Urea gel of in vitro transcribed (IVT) tRNAs and commercial *S. cerevisiae* $tRNA^{Phe}$ showing the relative lengths and purity. The experiment was repeated independently twice with similar results.

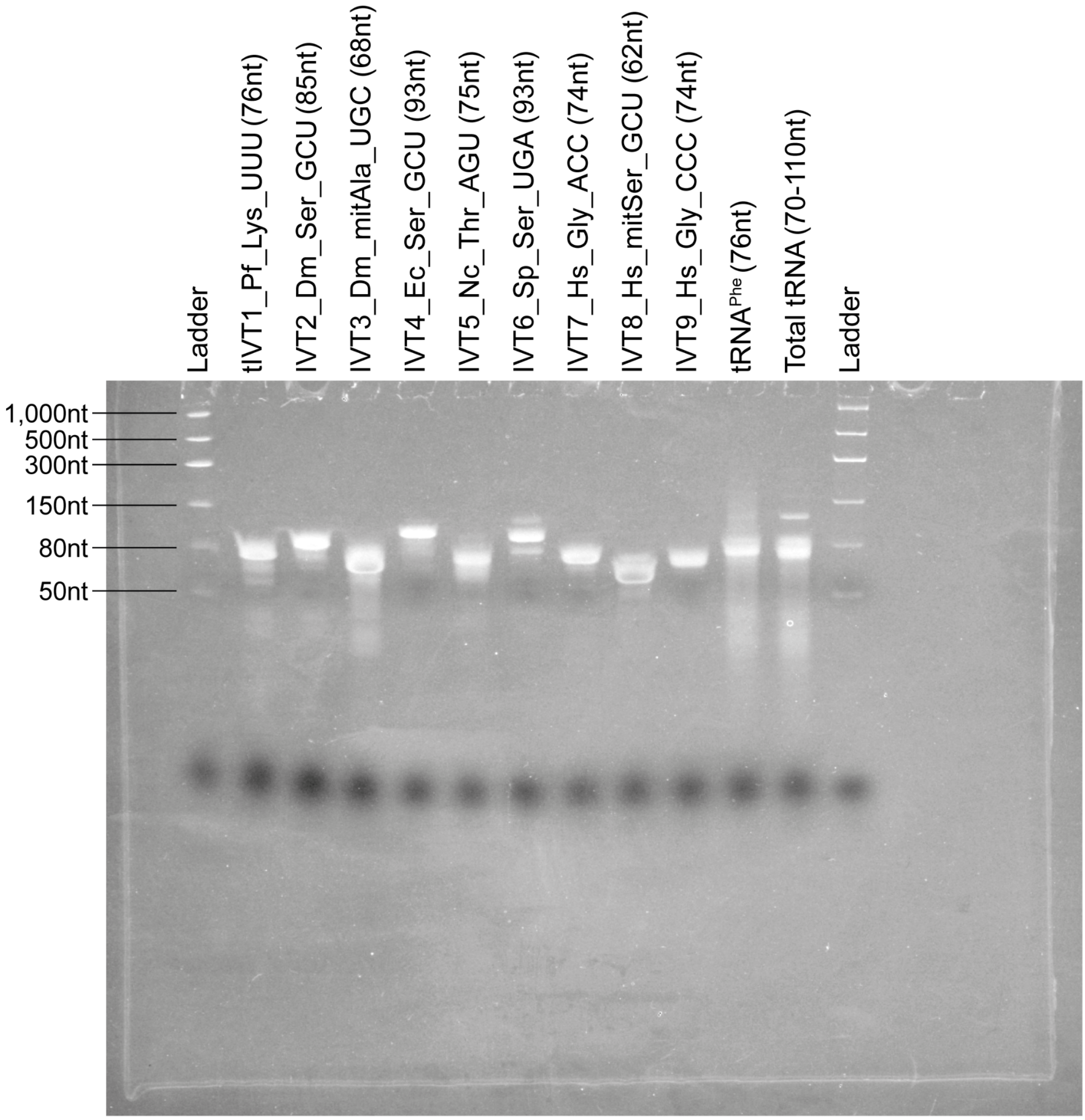

Supplement: Supplementary file 1 — Supplementary Figs. 1–25. [file 41587_2023_1743_MOESM1_ESM.pdf]
