# Supplementary material for: Quantitative analysis of tRNA abundance and modifications by nanopore RNA sequencing
Source: Nat Biotechnol. 2023 Apr 6;42(1):72–86. doi: 10.1038/s41587-023-01743-6 (PMC10791586; doi:10.1038/s41587-023-01743-6)
Supplement: Supplementary file 2 — Reporting Summary [file 41587_2023_1743_MOESM2_ESM.pdf]

Corresponding author(s): Eva Maria Novoa
Last updated by author(s): Feb 28, 2023

Reporting Summary

Nature Portfolio wishes to improve the reproducibility of the work that we publish. This form provides structure for consistency and transparency in reporting. For further information on Nature Portfolio policies, see our Editorial Policies and the Editorial Policy Checklist.

Statistics

For all statistical analyses, confirm that the following items are present in the figure legend, table legend, main text, or Methods section.

n/a	Confirmed	
☐	☒	The exact sample size (*n*) for each experimental group/condition, given as a discrete number and unit of measurement
☐	☒	A statement on whether measurements were taken from distinct samples or whether the same sample was measured repeatedly
☐	☒	The statistical test(s) used AND whether they are one- or two-sided<br>*Only common tests should be described solely by name; describe more complex techniques in the Methods section.*
☐	☒	A description of all covariates tested
☐	☒	A description of any assumptions or corrections, such as tests of normality and adjustment for multiple comparisons
☐	☒	A full description of the statistical parameters including central tendency (e.g. means) or other basic estimates (e.g. regression coefficient) AND variation (e.g. standard deviation) or associated estimates of uncertainty (e.g. confidence intervals)
☐	☒	For null hypothesis testing, the test statistic (e.g. *F*, *t*, *r*) with confidence intervals, effect sizes, degrees of freedom and *P* value noted<br>*Give P values as exact values whenever suitable.*
☒	☐	For Bayesian analysis, information on the choice of priors and Markov chain Monte Carlo settings
☒	☐	For hierarchical and complex designs, identification of the appropriate level for tests and full reporting of outcomes
☐	☒	Estimates of effect sizes (e.g. Cohen's *d*, Pearson's *r*), indicating how they were calculated

Our web collection on statistics for biologists contains articles on many of the points above.

Software and code

Policy information about availability of computer code

Data collection	MinKNOW software version 21.06.0
Data analysis	Simulations were run using MinKNOW software version 21.06.0<br>The reference FASTA, alignment and modification bed files, custom MinKNOW configurations, and all code used to analyze the runs are publicly available on GitHub (https://github.com/novoalab/Nano-tRNAseq) [154].<br>The NanoRMS script is available at https://github.com/novoalab/nanoRMS [98].<br>Reads were basecalled using Guppy basecaller v3.6.1 in high-accuracy (hac) mode.<br>Basecalled reads were mapped using minimap2 v2.17-r941 or BWA v0.7.17-r1188.<br>Differentially expressed tRNAs were inferred using DESeq2 [151].<br>Volcano plots were generated using the EnhancedVolcano package [152].

For manuscripts utilizing custom algorithms or software that are central to the research but not yet described in published literature, software must be made available to editors and reviewers. We strongly encourage code deposition in a community repository (e.g. GitHub). See the Nature Portfolio guidelines for submitting code & software for further information.

Data

Policy information about availability of data

All manuscripts must include a data availability statement. This statement should provide the following information, where applicable:

- Accession codes, unique identifiers, or web links for publicly available datasets
- A description of any restrictions on data availability
- For clinical datasets or third party data, please ensure that the statement adheres to our policy

Base-called FAST5 nanopore data FAST5 files have been deposited in the European National Archive (ENA) under accession PRJEB55684. From this data, both basecalled FAST5 and/or FASTQ files can be acquired [153]. FASTQ from HydraPsiSeq data [121] has also been deposited in ENA under accession PRJEB55684 [153]. A description of all the runs used in this work is included in Table S7 and Table S27. The list of tRNA modifications present in S. cerevisiae tRNAs was obtained from MODOMICS (https://iimcb.genesilico.pl/modomics/sequences/), and were retrieved on the 21st of Sep 2021. tRNA expression estimates from Illumina-based S. cerevisiae tRNA sequencing were obtained from following sources: mim-tRNAseq (GEO: GSE152621), tRNA-HydroSeq (Supplementary Material of the publication) and ARM-seq (Supplementary Table 2 of the publication).

Human research participants

Policy information about studies involving human research participants and Sex and Gender in Research.

Reporting on sex and gender	N/A
Population characteristics	N/A
Recruitment	N/A
Ethics oversight	N/A

Note that full information on the approval of the study protocol must also be provided in the manuscript.

Field-specific reporting

Please select the one below that is the best fit for your research. If you are not sure, read the appropriate sections before making your selection.

☒ Life sciences ☐ Behavioural & social sciences ☐ Ecological, evolutionary & environmental sciences

For a reference copy of the document with all sections, see nature.com/documents/nr-reporting-summary-flat.pdf

Life sciences study design

All studies must disclose on these points even when the disclosure is negative.

Sample size	N/A
Data exclusions	None
Replication	2 or 3 biological replicates, as indicated for each figure, details found in Supplementary Table 2 of the publication
Randomization	N/A
Blinding	N/A

Reporting for specific materials, systems and methods

We require information from authors about some types of materials, experimental systems and methods used in many studies. Here, indicate whether each material, system or method listed is relevant to your study. If you are not sure if a list item applies to your research, read the appropriate section before selecting a response.

Materials & experimental systems

n/a	Involved in the study
☒	☐ Antibodies
☐	☒ Eukaryotic cell lines
☒	☐ Palaeontology and archaeology
☒	☐ Animals and other organisms
☒	☐ Clinical data
☒	☐ Dual use research of concern

Methods

n/a	Involved in the study
☒	☐ ChIP-seq
☒	☐ Flow cytometry
☒	☐ MRI-based neuroimaging

Eukaryotic cell lines

Policy information about cell lines and Sex and Gender in Research

Cell line source(s)	Saccharomyces cerevisiae parental strain (BY4741), Pus1 knockout strain (BY4741 MATa pus1::KAN), Pus4 knockout strain (BY4741 MATa pus4::KAN), and Pus7 knockout strain (BY4741 MATa pus7::KAN) were obtained from the Yeast Knockout Collection (Dharmacon)
Authentication	Cells were authenticated by Dharmacon prior to purchase
Mycoplasma contamination	Cells were negative for mycoplasma contamination
Commonly misidentified lines (See ICLAC register)	N/A
